# Surface hydrophobicity of slippery zones in the pitchers of two *Nepenthes* species and a hybrid

**DOI:** 10.1038/srep19907

**Published:** 2016-01-27

**Authors:** Lixin Wang, Qiang Zhou

**Affiliations:** 1 School of Mechanical Engineering, Hebei University of Science and Technology, Shijiazhuang 050018, China; 2 College of Engineering, China Agricultural University, Beijing 100083, China

## Abstract

To investigate the hydrophobicity of slippery zones, static contact angle measurement and microstructure observation of slippery surfaces from two *Nepenthes* species and a hybrid were conducted. Marginally different static contact angles were observed, as the smallest (133.83°) and greatest (143.63°) values were recorded for the *N. alata* and *N. miranda* respectively, and the median value (140.40°) was presented for the *N. khasiana*. The slippery zones under investigation exhibited rather similar surface morphologies, but different structural dimensions. These findings probably suggest that the geometrical dimensions of surface architecture exert primary effects on differences in the hydrophobicity of the slippery zone. Based on the Wenzel and Cassie-Baxter equations, models were proposed to analyze the manner in which geometrical dimensions affect the hydrophobicity of the slippery surfaces. The results of our analysis demonstrated that the different structural dimensions of lunate cells and wax platelets make the slippery zones present different real area of the rough surface and thereby generate somewhat distinguishable hydrophobicity. The results support a supplementary interpretation of surface hydrophobicity in plant leaves, and provide a theoretical foundation for developing bioinspired materials with hydrophobic properties and self-cleaning abilities.

Carnivorous plants of the genus *Nepenthes* depend on highly evolved organs called pitchers situated at the tips of their conspicuous leaves^1^,^2^ to efficiently capture and digest predominant arthropods^3^,^4^,^5^. This process can provide almost the entire nutrients supply to the *Nepenthes* plants that grow in infertile habitats^6^,^7^. Considering the wide divergence in macro-morphologies and surface architecture that perform various functions, the *Nepenthes* pitchers have been typically distinguished by several functional parts: a leaf-shaped lid, a collar-formed peristome, a transitional part, a slippery zone and a digestive region^8^,^9^. Evolutionarily optimized, these different parts fulfill the functions of attracting and trapping insects, retaining and digesting prey, and absorbing nutrients^8^. In recent years, both the morphological structure and the predation function of the pitchers have gradually become the focus of numerous studies, attempting to not only explore their prey efficiency and anti-attachment mechanism^8^,^10^, but also establish biological models for the biomimetic development of insect slippery trapping plate^11^,^12^ and other bio-inspired materials with excellent anti-adhesive properties^13^,^14^,^15^.

Within most *Nepenthes* plants, the slippery zone bears numerous downward-oriented lunate cells and a layer of irregular epicuticular wax coverings^12^,^16^,^17^,^18^,^19^. These extraordinary structures present rather unique slippage property to most arthropods, thereby perform the role of trapping and retaining prey^20^,^21^,^22^. Microstructure studies have confirmed that the relatively dense wax coverage is well distinguished by two superimposed layers, namely the upper layer and the lower layer, which differ in ultrastructure, composition and mechanical properties^16^,^18^,^19^,^23^,^24^. Studies involving behavioral observations and force measurements revealed that the platelet-shaped wax coverings via the contamination effect on insect adhesive pads and/or the reduction contact area between the slippery surface and the pads to restrict insect attachment ability^10^,^11^,^18^,^24^. Recent studies have stressed that the wax coverings are mechanically stable enough to resist insect attachment, and the roughness of the microscopic surface is sufficient to prevent the smooth flexible insect pads from attaching to the slippery surface^24^. In addition, with aspect to morphological characteristics and distribution density, lunate cells also contribute considerably to the anti-attachment function by reducing the real contact area^10^,^25^. To maintain these superior anti-attachment properties, it is essential to preserve the architecture of the slippery surface from being contaminated by dust and other contaminants. From a macroscopic view, the slippery surface of *Nepenthes* pitchers in natural habitats appears glaucous and considerably clean, indicating that the slippery surface probably possesses both self-cleaning ability and hydrophobicity. Previous investigations reported the wettability of different regions in *Nepenthes alata* pitchers, revealing that the slippery zone bears a rather high static contact angle and considerably low free surface energy^23^. However, this study did not present the reasons why the slippery surface possesses considerable hydrophobicity.

In the present study, the principal objective of our investigation involved the static contact angle measurement and the three-dimensional microstructure examination, is to explore the hydrophobicity of the slippery surfaces of three types of *Nepenthes* pitchers, also propose a scientific explanation for the hydrophobic mechanism.

## Results

### Hydrophobicity of various slippery surfaces

**Figure 1:**
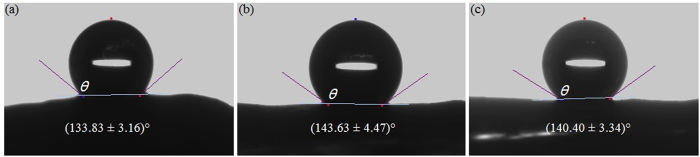
Static contact angles of distilled water on slippery surfaces of various *Nepenthes* species and hybrid. (**a**) *N. alata*, (**b**) *N. Miranda* (a hybrid), (**c**) *N. khasiana*. θ, static contact angle; number of measurements *n* = 16.

**Figure 2:**
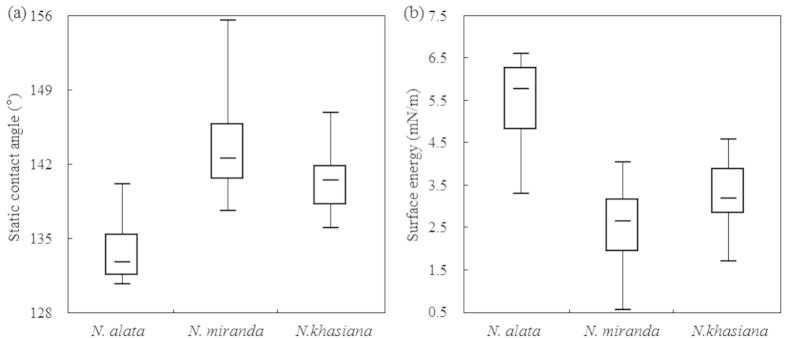
Box and whisker plot presenting the static contact angles (**a**) and surface energy (**b**) of slippery surfaces from various *Nepenthes* species. Horizontal lines represent the median, boxes denote the two inner quartiles, and whiskers represent the maximal and minimal values of the static contact angle and the surface energy.

To quantify the wettability of slippery surfaces in *Nepenthes* pitchers, we measured the static contact angle with distilled water droplets, and calculated the corresponding surface energy. The static contact angles recorded for the studied *Nepenthes* species and hybrid under investigation, as well as other *Nepenthes* species only presented in the supplementary information, approximately ranged from 128° to 156° (Fig. 1 and S1–S3), indicating that the slippery surfaces of these selected *Nepenthes* pitchers possess hydrophobic property. Statistical results (Fig. 1 and S3) suggested that the slippery surface of *N. alata* pitcher bears the lowest value of the static contact angle (mean ± SD: 133.83 ± 3.16°), whereas that of *N. miranda* and *N. khasiana* exhibit the highest (143.63 ± 4.47°) and the median values (140.40 ± 3.34°) respectively. For the slippery surface of a given *Nepenthes* species, the static contact angle values are usually within a relatively small range (Fig. 2a and S4). Furthermore, for the *Nepenthes* species and hybrid under investigation, the static contact angles recorded were somewhat different (Fig. 2a and S4, *t*-test, P < 0.001), suggesting that the slippery surfaces of the investigated *Nepenthes* species possess slightly disparate wettability.

Since the surface energy of these slippery zones was automatically calculated from their static contact angle values, these results exhibited an extremely divergent tendency (Fig. 2b and S4). Slippery surfaces of the *N. alat*a pitchers possessed the highest value of surface energy (5.41 ± 1.11 mN/m), whereas that of *N. miranda* presented the lowest value (2.51 ± 0.92 mN/m) and that of *N. khasiana* exhibited the median values (3.28 ± 0.91 mN/m). For the selected *Nepenthes* species and hybrid, the surface energy of the slippery zones exhibited different values (Fig. 2b, *t*-test, P < 0.001, S4).

### Surface morphology and structure of the slippery zones

**Figure 3:**
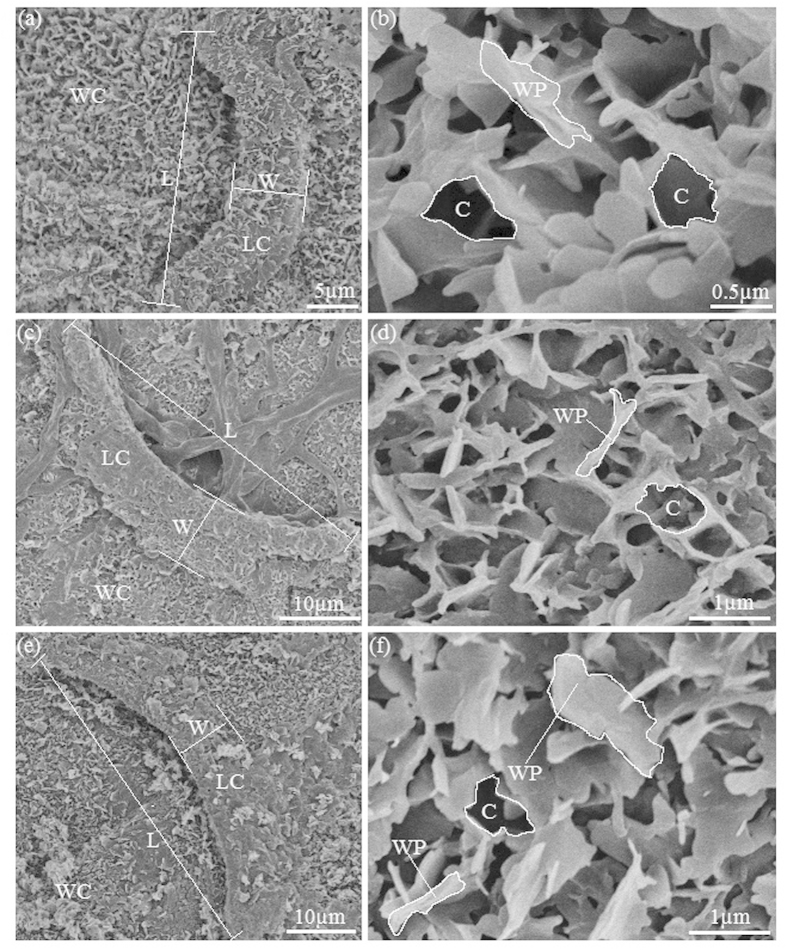
SEM micrographs showing the morphology of slippery surfaces in various *Nepenthes* species and hybrid. (**a,c,e**) Images showing micro-morphology of lunate cell and its surroundings in slippery zones of *N. alata*, *N. miranda* and *N. khasiana*, respectively. (**b,d,f**) High magnification micrographs showing platelet-shaped wax crystals of slippery surfaces in *N. alata*, *N. miranda* and *N. khasiana*, respectively. WC, wax coverings; LC, lunate cell; WP, wax platelet; C, cavity formed by the adjacent wax platelets; L, length of the lunate cell; W, width of the lunate cell.

The slippery zones of the *Nepenthes* species and hybrid under investigation contribute from one third to a half of the entire pitcher length, and the inner surface of these slippery zones presented a glaucous and spotless macro-morphology. As described in previous studies^10^,^12^,^16^,^19^,^22^,^26^, scanning electron microscope (SEM) images of the slippery surfaces of the *Nepenthes* plants under investigation, revealed that they were covered by relatively dense and continuous epicuticular wax coverings, with an ambulance of scattered prominent lunate cells (Fig. 3). The wax coverings emerged as discernible platelet-formed wax crystals with an irregular pattern, arranging extremely dense on the slippery surfaces, most of which overlapped each other, in such a manner that the generated cavities were hardly distinguishable (Fig. 3). A large number of lunate cells with relatively great geometrical dimensions were singly distributed over the slippery surfaces. Each lunate cell had both ends bent toward the pitcher bottom corresponds to an exclusive and enlarged overlapping guard cell, generating a crescent-shaped profile with an asymmetrical convex surface.

The platelet-formed wax crystals exhibited highly similar arrangements and shapes on different parts of the slippery surfaces in the *Nepenthes* species and hybrid under investigation. Geometrical dimensions of the wax platelets showed slight variations among the *Nepenthes* species and hybrid, as values for the apparent length and thickness ranged from 0.8–1.1 μm and 80–100 nm, respectively. The cavities formed by adjacent wax platelets presented an irregular appearance and varied noticeably among the three *Nepenthes* plants under investigation. Lunate cells from these *Nepenthes* species possessed high similarity in morphology, but presented apparent differences in size and distribution density. Since the geometrical dimensions of wax crystals and lunate cells in many *Nepenthes* species have been statistically analysed in previous studies^10^,^18^,^21^,^25^,^26^, we analysed and reported only those structural parameters related to surface hydrophobicity in the present study, as shown in Table 1 and supplementary information S5.

**Figure 4:**
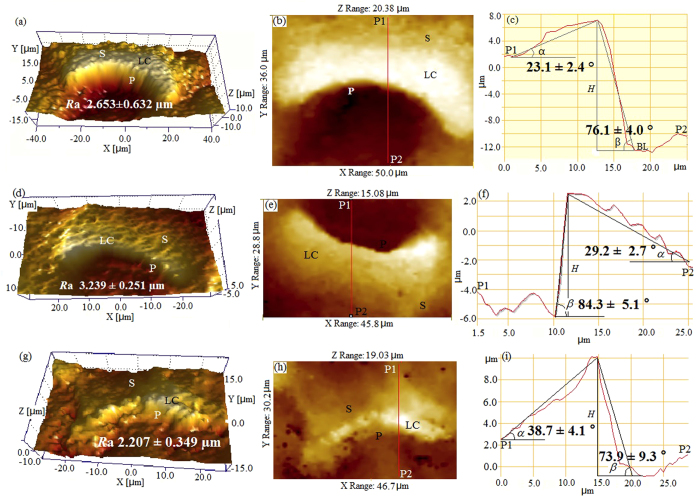
Scanning white light interferometer (SWLI) images showing the 3D structure of the lunate cells and their surroundings in the selected *Nepenthes* species. *N. alata* (**a–c**), *N. miranda* (**d–f**) and *N. khasiana* (**g–i**). (**a,d,g**) Specific 3D images of the lunate cells, showing rather uneven surface morphology. (**b,e,h**) 2D micrographs showing the profiles of the lunate cells. (**c,f,i**) Height variations in the lunate cells along the red line shown in (**b,e,h**), respectively. LC, lunate cell; P, precipice generated by the lunate cell from upside to underside in a downward orientation; S, slope generated by the lunate cell from upside to underside in a upward orientation; *R*a, arithmetic average roughness; P1, starting point of the red line; P2, terminal point of the red line; α, inclined angle of the slope; β, inclined angle of the precipice; *H*, height of the lunate cell.

**Figure 5:**
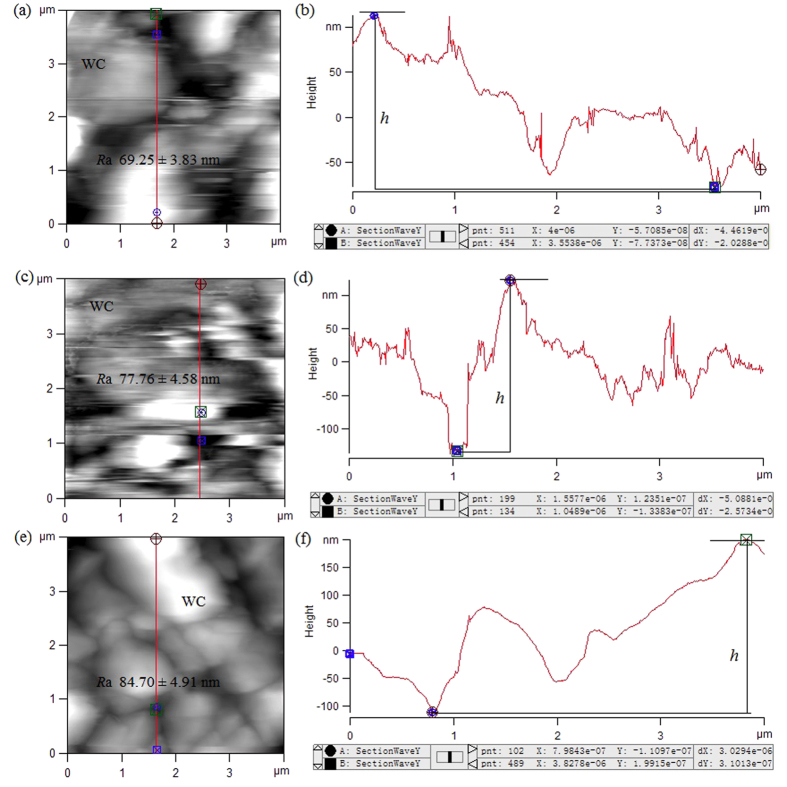
Atomic force microscope (AFM) examinations showing the slippery surfaces (excluding lunate cells) of the selected *Nepenthes* species. *N. alata* (**a,b**), *N. miranda* (**c,d**) and *N. khasiana* (**e,f**). (**a,c,e**) 2D images showing the profiles of the wax coverings in the slippery surfaces. (**b,d,f**) Height variations in the platelet-shaped wax coverings along the red line shown in (**a,c,e**), respectively. WC, wax coverings of the slippery surface; *R*a, arithmetic average roughness; *h*, height of the platelet-shaped wax coverings.

To acquire morphological and structural information of the slippery surfaces in a vertical orientation, such as height and surface roughness, we conducted scanning white-light interferometer (SWLI) observations and atomic force microscopic (AFM) measurements. In the *Nepenthes* species and hybrid under investigation, the overall slippery surfaces were undulating, and exhibited rather uneven morphologies (Fig. 4). The lunate cells protruded from the slippery surfaces (about 8–15 μm higher) and exhibited noticeable variations in elevation, shifting slowly from underside to upside in an upward direction and sharply from upside to underside in a downward direction, thereby forming ‘slope’ and ‘precipice’ respectively (Fig. 4a,b,d,e,g,h), and generating the slope and precipice angles (Fig. 4c,f,i). Geometrical dimensions and their statistical analysis of the lunate cells in a vertical orientation are presented in Table 1. As relatively large values in height were recorded for these lunate cells (Table 1), they contributed significantly to the degree of surface roughness of the slippery zones. When examining the slippery surfaces over a large expanse (containing several lunate cells), the surface roughness presented rather high values (*R*a 2.207–3.239 μm, Fig. 4a,d,g). Within a small scanning area (4 × 4 μm^2^, excluding the lunate cell), AFM images revealed that the wax coverings possess a relatively smooth surface, and present rather small values of surface roughness (*R*a 69.25–84.70 nm, Fig. 5a,c,e). The height data for the platelet-shaped wax coverings were statistically derived according to the height variations in the slippery surfaces (Fig. 5b,d,f), and are exhibited in Table 1 and supplementary information S5.

## Discussion

Our exhibited results concerning the static contact angle quantitatively described the hydrophobicity of the slippery surfaces from two *Nepenthes* species and a hybrid; we also presented the micro-morphology and surface architecture of these slippery zones. Based on these results, in the following section, we propose models to theoretically analyze the influence of surface structures on the slippery zone’s hydrophobicity.

### Comparison of the slippery surface’s hydrophobicity

During insect capturing, the slippery zones of most *Nepenthes* species depend on their particular surface structures to conspicuously restrict insect’s attachment ability, thereby performing the functions of insect trapping and prey retention^9^,^16^,^17^,^19^. The slippery zones of *Nepenthes* plants growing in natural habitats are frequently subjected to contamination by dust and rainwater. It is therefore crucial to possess self-cleaning abilities, which enables the surface morphology to render superior slippage function continually for the efficient capture of prey. In the *Nepenthes* plants under investigation, relatively high values of static contact angles (128–156°, Fig. 1 and S1–S4) and rather low surface energy (0.54–7.46 mN/m, Fig. 2b and S1–S4) were recorded for all slippery surfaces, suggesting that these slippery zones present significant surface hydrophobicity. A previous study presented similar results for the slippery surface of *N. alata* pitchers, the static contact angle reported was approximately 160° and the surface energy was about 4 mN/m^23^. These results probably suggest that the slippery surfaces of various *Nepenthes* species and hybrids exhibit hydrophobic property. However, slight differences in values recorded for both the static contact angle and the surface energy in our study potentially suggest that the slippery surfaces from these *Nepenthes* plants possess marginally distinguishable hydrophobic property.

### Effects of surface structure on hydrophobicity

In the past decade, interest in the surface structures and hydrophobicity of various plant leaves has increased rapidly, attempting to use these biostructures as templates to create materials with self-cleaning capability and other desirable properties. The leaf surface of lotus plants, which bears hierarchical structures on a micro-nano scale, yields a rather high static contact angle and has been considered as the typical bio-template to create biomimetic materials^14^,^27^,^28^. Chemical composition and surface topography are two crucial factors that determine the surface hydrophobicity^29^, and almost the entire primary surface of plant leaves is covered by a mixture of hydrophobic compounds referred to as wax crystals^30^,^31^. Since water contact angles on smooth hydrophobic surfaces are generally lower than 120°, the surface micro/nanostructure and their generated roughness is primarily responsible for hydrophobicity at much greater contact angles^32^.

To describe the effects of roughness on hydrophobicity, Wenzel first hypothesized that the existence of a rough surface makes the real contact area between solid and liquid is much higher than their geometrical contact area, leading to the improvement in hydrophobicity or hydrophilicity^33^,^34^. Furthermore, according to this hypothesis, an equation was proposed to quantitatively express the effect of surface roughness on contact angle, as follows:


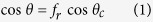


where 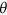 and 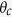 respectively represent the contact angle on rough and smooth surfaces, and 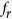 represents the roughness factor defined as the ratio between the real rough surface area and the geometrical surface area or projected area of the rough surface. The essential condition of this equation is that there is adequate contact between the liquid and solid, without air between the two substances. In fact, however, when the material surface exhibits hydrophobicity (contact angle higher than 90°), the air is usually entrapped between the water droplet and the surface topography. Therefore, Cassie and Baxter^35^ modified Wenzel’s model to propose the composite liquid-gas-solid interface, and an extended equation to calculate the contact angle on the composite interface, as follows:


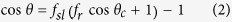


where 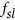 is the fractional flat geometrical area of the solid-liquid under the liquid droplet, and 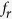 is the roughness factor of the solid-liquid interface. This equation is generally employed to analyse the contact angle of almost all the composite liquid-solid interfaces, and predicts the entrapped air between the liquid droplet and the solid surface serves a crucial role in surface hydrophobicity.

According to the foregoing equations, the surface topography and its generated roughness performs a crucial role in affecting the apparent contact angle of material surface, as enhancing or decreasing the contact angle when the material surface possesses hydrophobicity or hydrophilicity, respectively. In the slippery surfaces of *Nepenthe* plants, the roughness induced by the presence of both lunate cells and wax crystals causes the apparent contact angle (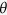) to be greater than the smooth contact angle (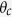). Moreover, the wax coverings with structural dimensions in the nano-micro range are responsible for the first generation of surface roughness, and the lunate cells with the micrometer ranged dimensions contribute to the second generation of surface roughness. This dual-scale surface topography is very similar to the hierarchical architecture of the lotus leaf, possibly conferring a similar function for the surface hydrophobicity.

### Hydrophobicity induced by micron-sized lunate cells

**Figure 6:**
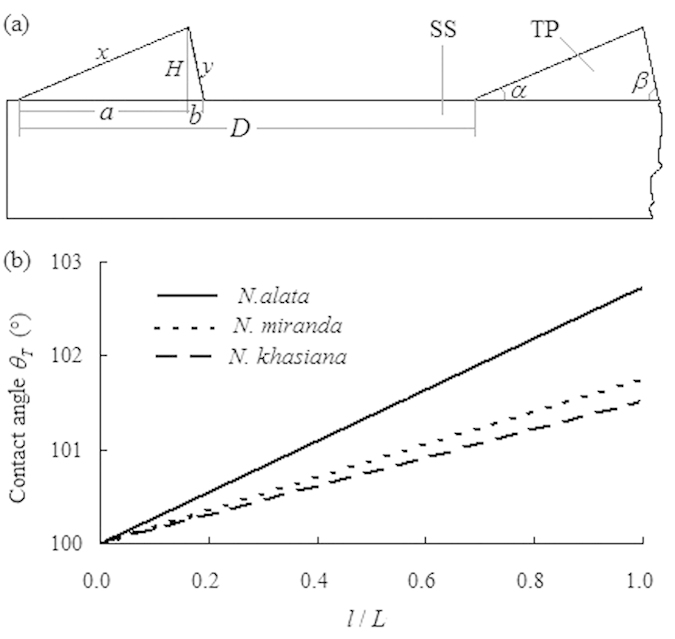
Simplified model of the slippery surface, as a flat plane with equally distributed triangular prisms (**a**); and theoretical calculation of the contact angle of a water droplet on the slippery surface based on the Wenzel equation (**b**). SS, simplified slippery surface; TP, triangular prism, namely the simplified lunate cell; 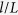, the ratio between the length of simplified lunate cell (triangular prism) and the width of the slippery surface.

In our static contact angle measurements, the volume of a water droplet was approximately 3–5 μL, generating the static contact angle ranges of 128–156° (including the static contact angle values presented in supplementary information S1–S4), indicating that the contact radius of the water droplet on the slippery surfaces is approximately 0.364–0.836 mm, thus covering quite a large number (approx. 65–525) of lunate cells. Moreover, the lunate cells with structural dimensions of ~10 μm contribute significantly to the surface roughness of slippery zones, possibly serving an important role in conferring hydrophobicity. In order to analyze this possibility succinctly, we simplify the lunate cell as a triangular prism, retaining its slope and precipice angles. Consequently, the slippery surface is considered as a flat smooth plane with equally distributed triangular prisms of same dimensions, as presented in Fig. 6a.

In this simplified model, following the Wenzel equation (Eq. 1), the equation regarding the theoretical contact angle 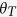 of a water droplet on the slippery surfaces can be deduced (Supplementary information S6 showing the derivation) as follows:


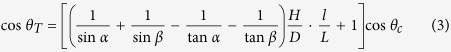


where 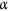 and 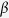 are the slope and precipice angles respectively, formed by the lunate cell’s particular structure (Fig. 4); 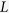 is the width of the simplified slippery surface; 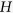 and 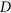 are the height and interval distance of the adjacent lunate cells (Table 1) respectively; and 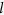 is the length of the simplified lunate cell (triangular prism), where 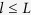. In previous investigations, the true contact angle (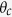) for water droplets on smooth wax coverings of plants has been reported as 100°^ ^36,37. Based on the mean values of the structural dimensions (Table 1 and S5) and the true contact angle (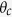), the contact angle (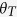) can be theoretically calculated. The calculation results (Fig. 6b) present the maximal values of (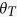) as approximate 102.7° in *N. alata*, 101.8° in *N. miranda*, and 101.5° in *N. khasiana*, exhibiting marginal differences that could be exclusively attributed to the different structural parameters of the lunate cells from the two *Nepenthes* species and a hybrid under investigation. These theoretically calculated values are considerably lower than their actual measured results, probably suggesting that the lunate cells are not the only features that confer properties of superior hydrophobicity to the slippery surfaces.

### Hydrophobicity induced by nano-scale wax coverings

**Figure 7:**
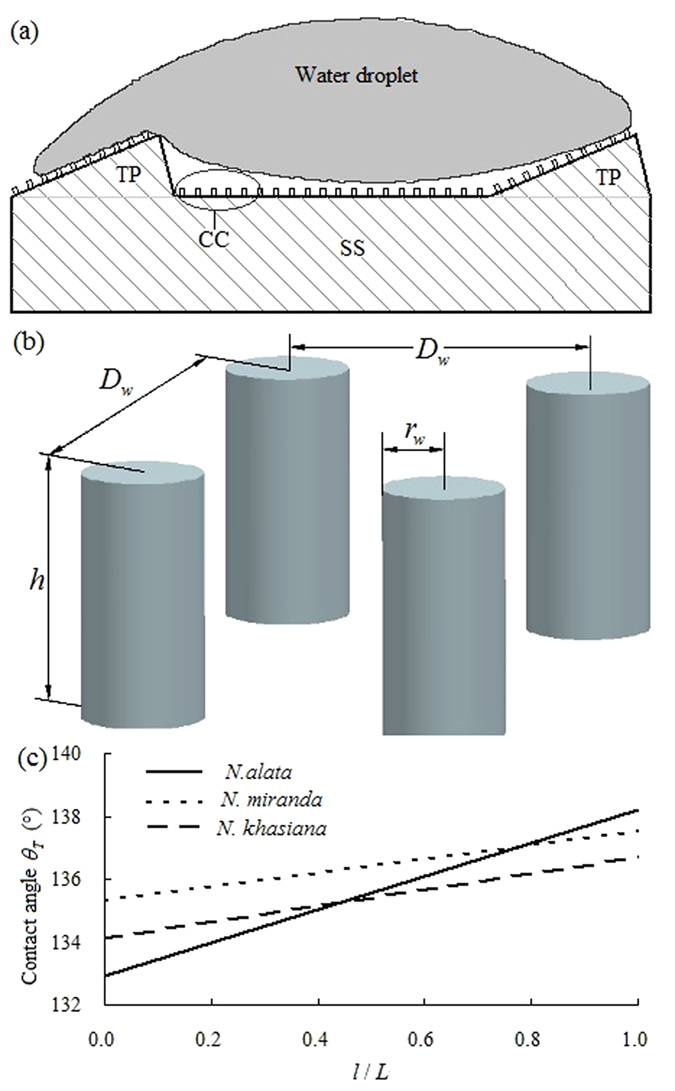
Schematic depiction of the simplified slippery surface, as a flat plane covered by equally distributed triangular prisms and an array of convex cylinders (**a**), the array of convex cylinders showing structural parameters (**b**), and the theoretical calculation of the contact angle of a water droplet on the simplified slippery surface based on the Cassie-Baxter equation (**c**). SS, simplified slippery surface; TP, triangular prism, namely the simplified lunate cell; CC, convex cylinder, namely the simplified platelet-formed wax crystal; 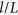, the ratio between the length of the simplified lunate cell (triangular prism) and the width of the slippery surface.

On slippery surfaces, wax coverings consisting of platelet-shaped crystals with nano-scale structural parameters are interactively distributed (Fig. 3b,d,f), forming numerous cavities which probably entrap air when a water droplet makes contact with the wax surface. According to a previous theory^35^, the existence of entrapped air can greatly improve the surface hydrophobicity. To succinctly analyze the influence of nano-scale wax coverings on the hydrophobicity of the slippery surface, as well as based on the structural parameters recorded with AFM and SEM equipments, the wax coverings and their generated cavities were simplified as convex cylinders with a particular interval distance. Therefore, the slippery surface was considered as a flat smooth plane with equal-distance distributed triangular prisms and an array of convex cylinders with a particular interval distance (Fig. 7a).

In this simplification, the wax platelets not only significantly improve the real rough surface area, but also depend on the spaces generated by the nano-scale convex cylinders to give the possibility of entrapping air. The real rough surface area results form two aspects basically: the triangular prisms (lunate cells) and the convex cylinders (platelet-shaped wax coverings); therefore, we can deduce the roughness factor 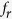 (Supplementary information S7 showing the derivation), as follows:


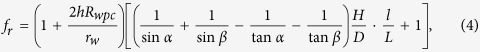


where 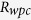 is the ratio between the area of platelet-shaped epicuticular wax coverings and the area of the entire slippery surface, 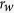 and 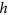 represent the radius and the height of the simplified platelet-formed wax crystal (convex cylinder, Fig. 7b), respectively. Furthermore, the projection of the single platelet-profiled wax crystal is succinctly considered as a circular region; therefore, based on the exhibited area values of the wax platelets (Table 1 and S5), we obtained the 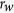. Combined with the Cassie-Baxter equation (Eq. 2), the relationship between theoretical contact angle 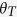 and the structural parameters of the slippery surfaces can be deduced, as follows:


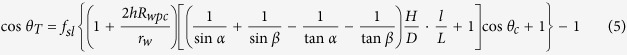


where, 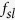 represents the ratio between the area of the platelet-shaped epicuticular wax coverings and the area of the entire slippery surface, namely 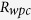. Based on the mean values of the structural dimensions (Table 1 and S5) of the slippery surfaces, the contact angle (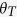) can be theoretically evaluated. The results of this evaluation (Fig. 7c) reveal the contact angle values of *N. alata* as 132.9–138.2°, whereas those of *N. miranda* and *N. khasiana* as 135.3–137.5° and 134.1–136.7° respectively, presenting somewhat detectable differences among the *Nepenthes* plants under study.

In comparison to the evaluation results, the simplified model, in which the surface is barely covered by triangular prisms, these calculated contact angle values are noticeably greater, attributing to the conspicuous enhancement of the roughness factor (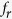) provided by the array of convex cylinders (simplified wax coverings). These findings suggest that the lunate cell is not the only factor that confers strong hydrophobicity to the slippery surfaces present, and the platelet-shaped wax coverings potentially serve a rather important function. From another aspect, the theoretically acquired results present marginally smaller values than the experimentally measured static contact angles of the slippery zones from the two *Nepenthes* species and a hybrid (Fig. 1). In fact, because the selected *Nepenthes* pitchers were grown in a nursery, they were continually subjected to a variety of insults, such as dust contamination or physical damage inflicted by captured insects, which attributed to the damage of the slippery zone’s surface topography in some extent, thereby the theoretical results should be moderately greater than the measured results. The precision required in acquiring the dimensions of the surface architecture, especially those of the nano-ranged wax crystals, is presumably responsible for the presented deviation between the theoretical and measured results of the contact angle. Besides the existed instruments used in our studies, more advanced apparatus and analytical procedures are propitious to obtain highly accurate parameters, thereby enabling a better confirmation of our proposed models.

The theoretical calculations based on the proposed models possibly provide a scientific explanation to the surface hydrophobicity of the slippery zones in *Nepenthes* pitchers, and present a reasonable interpretation to the marginal differences in static contact angle of a water droplet on those slippery surfaces. According to our analysis, geometrical parameters of the triangular prisms and the array of convex cylinders lead to the divergent static contact angle values. In the *Nepenthes* species and hybrid under investigation, platelet-shaped wax coverings and lunate cells exhibited discrepant structural dimensions (Table 1 and S5), thereby leading to marginal differences in surface hydrophobicity of the three *Nepenthes*’ slippery zones. However, our investigations and proposed theoretical models are preliminary; future studies should focus on dynamic contact angle measurement, which should explain how the anisotropy of the slippery surface mainly results from the orientation-growing lunate cells, affects the hydrophobicity. The expected results would presumably present a much more comprehensive and detailed understanding of the surface wettability of various slippery zones.

In summary, the surface hydrophobicity of the slippery zones from two *Nepenthes* species and a hybrid was investigated, including static contact angle measurement, microstructure observation and theoretical analysis. Results showed that the static contact angle values of the slippery surfaces range from approximately 128° to 156°, and present marginal differences among the three varieties of slippery zones. Based on the architecture and geometrical dimensions of the slippery surfaces, theoretical models were proposed to analyze the hydrophobic mechanism. Results of our analysis demonstrated that the platelet-shaped wax coverings and the lunate cells are the primary factors to confer strong hydrophobicity to the slippery surfaces, and that the discrepant structural dimensions contribute to the marginal differences in static contact angle values. Our acquired conclusions possibly support another interpretation of the surface hydrophobicity of plant leaves, and provide a theoretical reference for the development of biomimetic materials with hydrophobic properties and self-cleaning abilities.

## Materials and Methods

### *Nepenthes* pitchers

**Figure 8:**
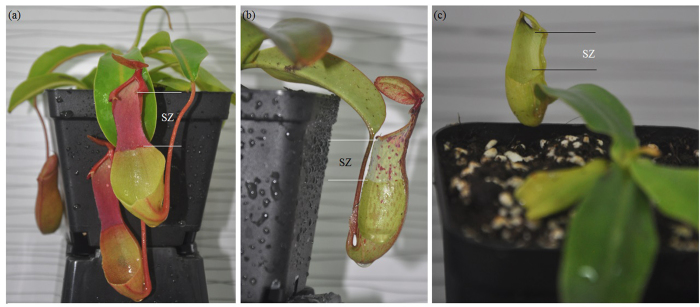
*Nepenthes* plants and their mature pitchers, used for static contact angle measurement and micromorphology observation. (**a**) *N. alata*, (**b**) *N. miranda* (a hybrid), (**c**) *N. khasiana*. SZ, slippery zone. (Taken and modified by Lixin Wang)

To investigate the hydrophobicity of the slippery surfaces, three varieties (two species and a hybrid) of *Nepenthes* plants (Fig. 8) were commercially acquired from a nursery (Hangzhou City, China) and cultivated in a small greenhouse under continuously controlled environmental conditions: temperature of 25–30 °C and relative humidity of 70–90%. Mature pitchers were clipped from the selected varieties and measured their length, as *N. alata*, 11.85 ± 0.57 cm, *n* = 4; *N. miranda* (a hybrid), 8.12 ± 0.36 cm, *n* = 4; and *N. khasiana*, 6.49 ± 0.34 cm, *n* = 4, mean length ± SD. For definitely presenting the range of the slippery zone, half of each pitcher was longitudinally cut with a pair of scissors, thereby allowing the slippery surface to be easily distinguished (Fig. 8). When growing, the pitchers were more or less exposed to contamination by various diverse particles, such as dust and wing scales of trapped insects. Therefore, each selected pitcher was rinsed several times with distilled water, to remove the contaminants without causing damage to the surface architecture of the slippery zones. The prepared slippery zones were used for static contact angle measurement and surface microstructure observation.

### Static contact angle measurement and surface energy estimation

Static contact angle measurement and surface energy estimation were conducted with an optical contact angle analyzer SL 150S (Solon Corp., USA). This equipment consists mainly of a high-speed (charge-couple device) CCD video system, a three-dimension manual micro-regulation unit and a micro-control module for syringes. The related software (CAST 2.0) performs static contact angle measurement and surface energy estimation according to standard evaluation methods and procedures. For measurement, several small pieces measuring 2 × 2 cm^2^ were cut from the central part of the rinsed slippery zones with a razor blade, and attached to a measuring platform using double-sided adhesive tape. Static contact angles of distilled water droplets on the slippery surfaces were measured with a needle-in sessile drop method, which the similar approach has been described in detail in previous literatures^23^,^38^,^39^. The volume of a distilled water droplet was approximate 3–5 μL. The static contact angles were evaluated by firstly using the circle-fitting estimation, followed by the Young-Laplace equation fitting calculation. For the slippery surfaces of each *Nepenthes* species and hybrid, 16 measurements were executed, and all the measurements were conducted under ambient conditions as follows: temperature of about 28 °C and relative humidity of about 45%. To minimize the shrinking effect induced by evaporation, the static contact angle measurements of the specimens were rapidly finished as soon as possible after being cut from the slippery zones.

When acquired the static contact angle value, surface energy of the slippery zones was calculated and presented automatically by the software CAST 2.0 belonged to the optical contact angle analyzer SL 150S. For this calculation, the Kwok method^40^ which universally applied for diverse surfaces was adopted.

### Surface architecture examinations

For obtaining structural information of the lunate cells and the wax coverings used to scientifically explain the hydrophobic mechanism, we examined the three-dimension architectures of the slippery surfaces in detail with a scanning electron microscope (SEM, Hitachi S-4800, Hitachi Corp., Japan), a atomic force microscope (AFM, MFP-3D Classic, Asylum Research, Oxford Instrument Corp., UK) and a scanning white-light interferometer (SWLI, Zygo NV-5000, Zygo Corp., USA).

Our SEM observation, followed similar methods of previous studies^10^,^25^,^41^, several pieces of approximately 1 × 1 cm^2^ were cut from the prepared slippery surfaces, and completely dried by natural evaporation in a dust-free environment. These dried samples were mounted on alloy blocks, sputter coated (Bal-Tec SCD005, Balzers, Switzerland) and observed. Geometrical dimensions of the surface architectures in a horizontal direction, including the area of a single wax platelet in the epicuticular wax coverings, the ratio between the area of platelet-shaped epicuticular wax coverings and the area of the entire slippery surface, as well as the length, width and interval distance of the lunate cells, were quantitatively analyzed from the saved images with the ImageJ software (ImageJ 1.38e/Java 1.5.0_09, National Institutes of Health, USA), and the acquisition process of some structural parameters is presented in supplementary information S8.

The SWLI examination was carried out to acquire geometrical information concerning the surface topography in a vertical direction. Fresh specimens (about 2 cm^2^) were cut from the processed slippery zones, attached to an aluminum block, and observed in the SWLI apparatus. The height information (~10 μm) of lunate cells was obtained via analyzing the saved images with the software (Scanning Probe Image Processor, Version 4.3.3.0, Image Metrology, Denmark) of the SWLI equipment. This software can definitively present the variation in height of the lunate cell’s profile along an established line, and the maximum height value during the variation can be derived.

For the AFM measurement, a high reflection coated triangular silicon nitride cantilever (Multi75E-G, Bugdetsensor, Nanoworld, Switzerland) was adopted to exhibit the topography of the wax coverings. Each specimen (about 2 cm^2^) was freshly cut from the prepared slippery zones and glued to a circular glass side, and completely submerged in ultrapure water to avoid structural shrinkage caused by evaporation. Scanning was conducted in tapping mode with an applied scanning force of about 13 nN and a constant scanning velocity (5 μm/s). Because the densely distributed lunate cells are characterised by relatively large values in height (~10 μm), the movement of the cantilever was frequently interrupted while scanning the wax coverings interspersed among the lunate cells. The scanning areas were therefore adjusted to within 4 × 4 μm^2^. After the AFM measurement, height data and surface roughness of the wax coverings were derived and presented.

## Additional Information

**How to cite this article**: Wang, L. and Zhou, Q. Surface hydrophobicity of slippery zones in the pitchers of two *Nepenthes* species and a hybrid. *Sci. Rep.* **6**, 19907; doi: 10.1038/srep19907 (2016).

## Supplementary Material

Supplementary Information

Supplementary Dataset S2

Supplementary Dataset S3

Supplementary Dataset S5

## Figures and Tables

**Table 1:** Structural parameters of wax coverings and lunate cells in slippery surfaces of two *Nepenthes* species and a hybrid.

*Nepenthes*	Epicuticular wax coverings			Length [*l*/μm]	Lunate cells			Density [/mm^2^]
	Height [*h*/nm]	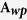 [μm^2^]	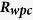 [%]		Width [*w*/μm]	Height [*H*/μm]	Distance [*D*/μm]	
*N. alata*	237.09 ± 11.78	0.102 ± 0.006	55.89 ± 2.42	44.38 ± 2.21	11.55 ± 0.60	20.41 ± 1.73	69.16 ± 2.99	234.7 ± 32.8
*N. miranda*	274.45 ± 12.18	0.194 ± 0.013	44.07 ± 1.67	51.36 ± 2.30	8.33 ± 0.36	12.04 ± 1.35	80.69 ± 3.21	155.5 ± 26.3
*N. khasiana*	301.03 ± 12.76	0.165 ± 0.009	51.37 ± 1.79	33.95 ± 1.75	9.79 ± 0.48	8.81 ± 0.79	64.19 ± 3.53	239.2 ± 46.2

Values are presented as mean ± SD. Number of measurements: in height and *R*_*wpc*_ of the epicuticular wax crystal n =

16; in A*wp* n = 20; in length, width and interval distance of the lunate cell n = 40; in lunate cell’s distribution density n

= 6; in lunate cell’s height n = 8. A_*wp*_, area of a single wax platelet in epicuticular wax coverings; *R*_*wpc*_, ratio between the area of the platelet-shaped epicuticular wax coverings and the area of the entire slippery surface.
