# Supplementary material for: Surface hydrophobicity of slippery zones in the pitchers of two Nepenthes species and a hybrid
Source: Sci Rep. 2016 Jan 27;6:19907. doi: 10.1038/srep19907 (PMC4728604; doi:10.1038/srep19907)
Supplement: Supplementary Information [file srep19907-s1.pdf]

Supplementary Information for

Surface hydrophobicity of slippery zones in the pitchers of two *Nepenthes* species and a hybrid

L. X. Wang [1*], Q. Zhou [2]

[1] School of Mechanical Engineering, Hebei University of Science and Technology, Shijiazhuang 050018, China

[2] College of Engineering, China Agricultural University, Beijing 100083, China

[*] Corresponding author, Email: ck_021@tom.com

S1. Images related to the measurement of static contact angle of the eight types of *Nepenthes* plants.

Note: *N. alata* 16 images, Page 2–7; *N. dyeriana* 16 images, Page 7–12; *N. fusca* 15 images, Page 13–17; *N. gracillima* 12 images, Page 18–21; *N. hookeriana* 12 images, Page 22–25; *N. khasiana* 16 images, Page 23–31; *N. mirabilis* 16 images, Page 31–36; N. miranda 16 images, Page 36–41

***N. alata* (16 images)**

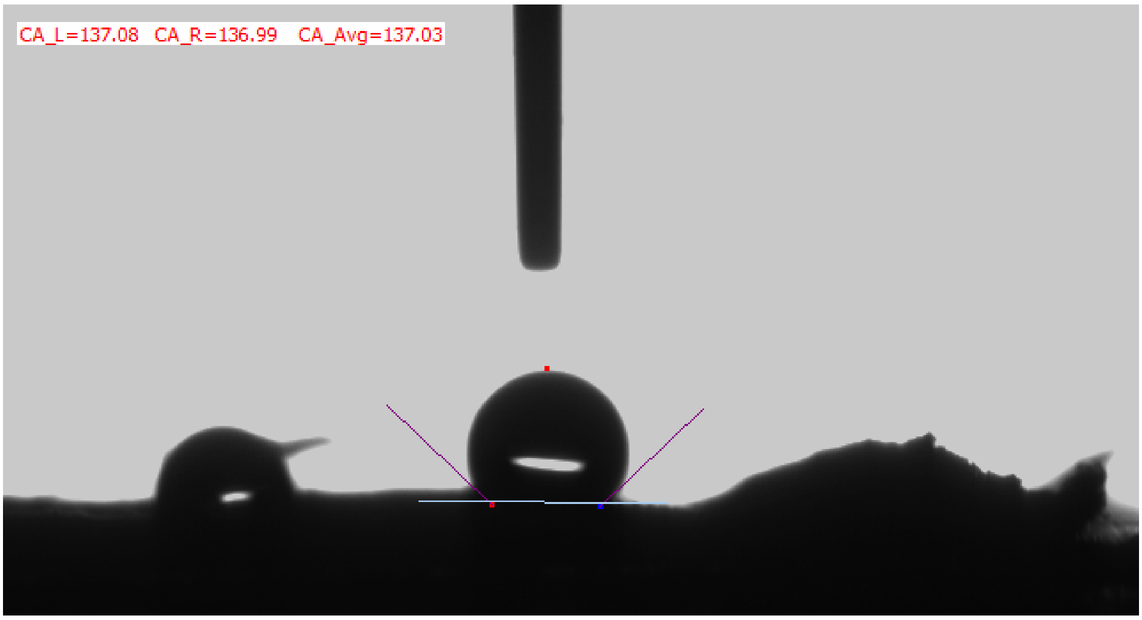

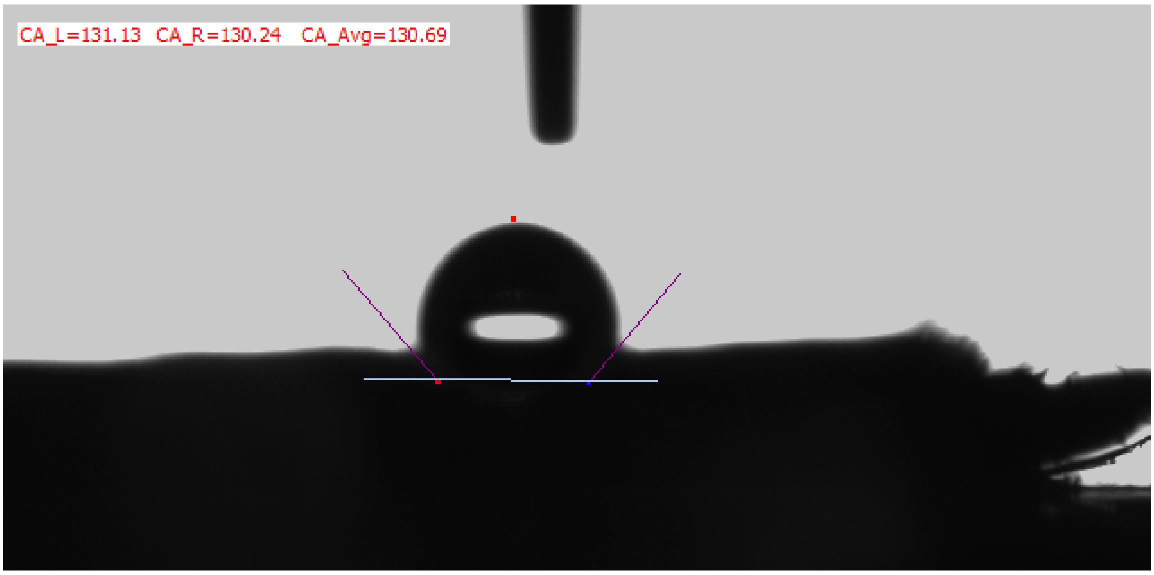

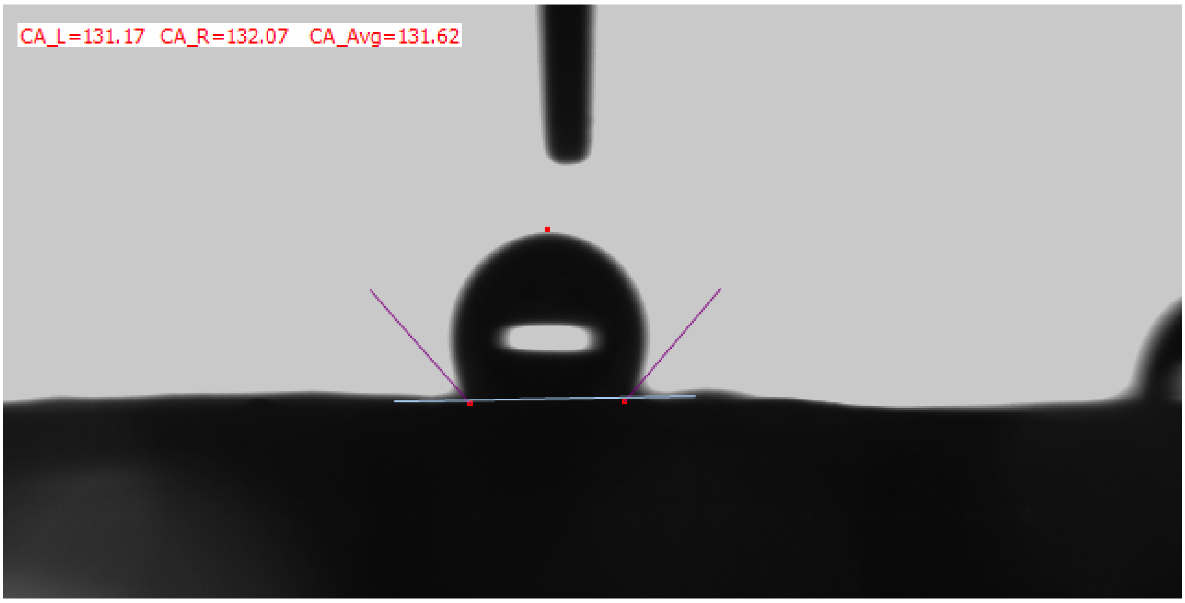
CA_L=131.17 CA_R=132.07 CA_Avg=131.62

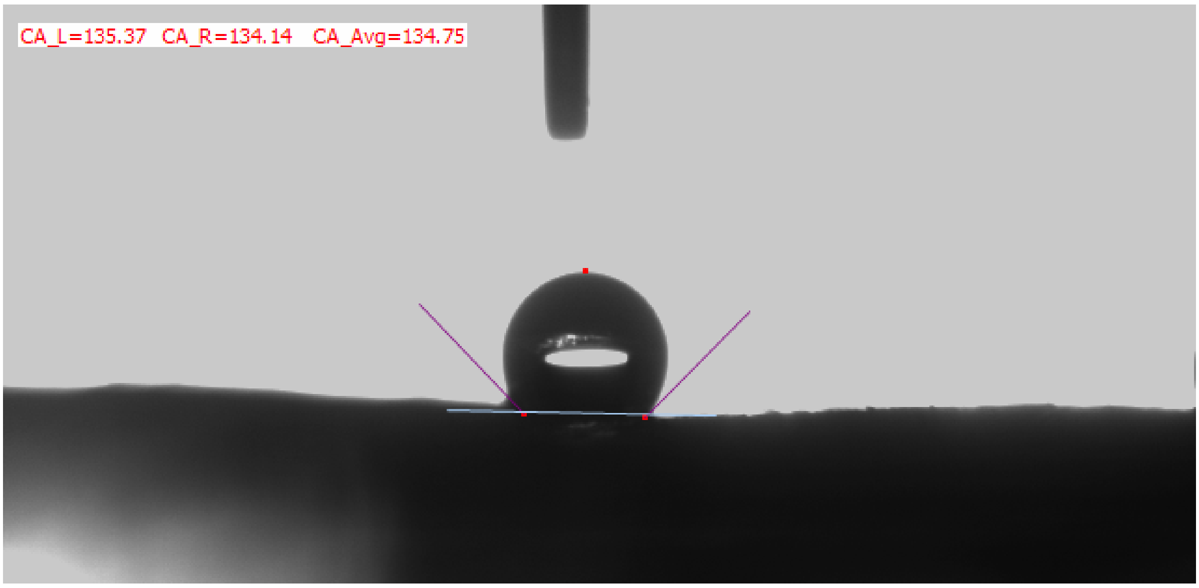
CA_L=135.37 CA_R=134.14 CA_Avg=134.75

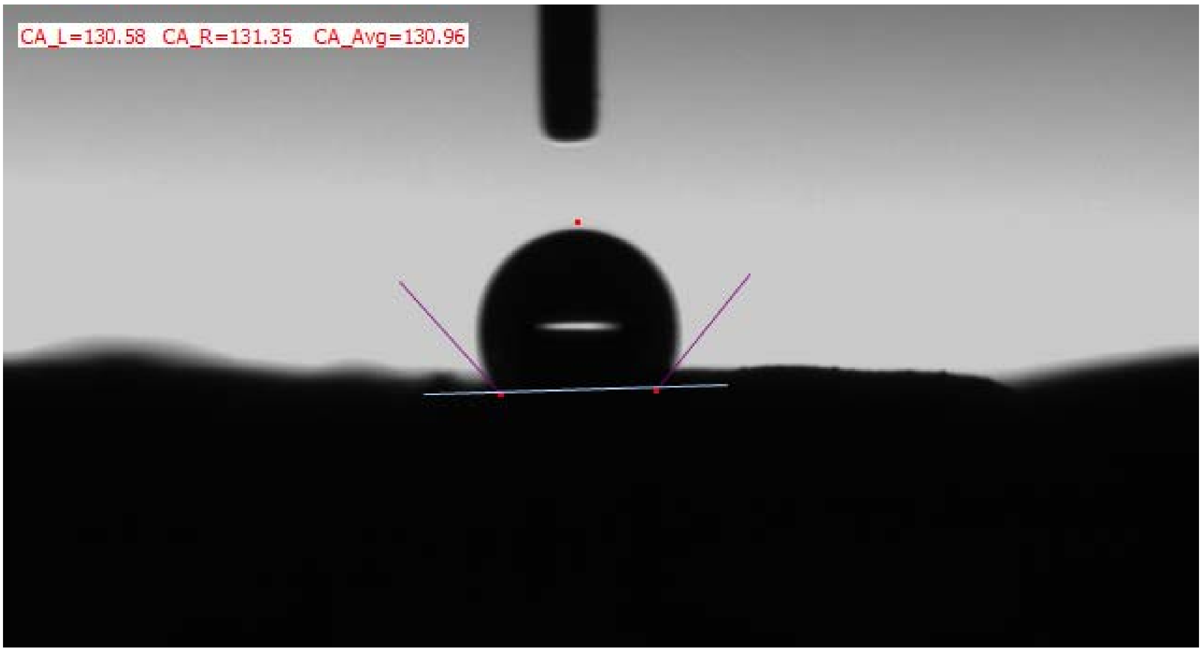
CA_L=130.58 CA_R=131.35 CA_Avg=130.96

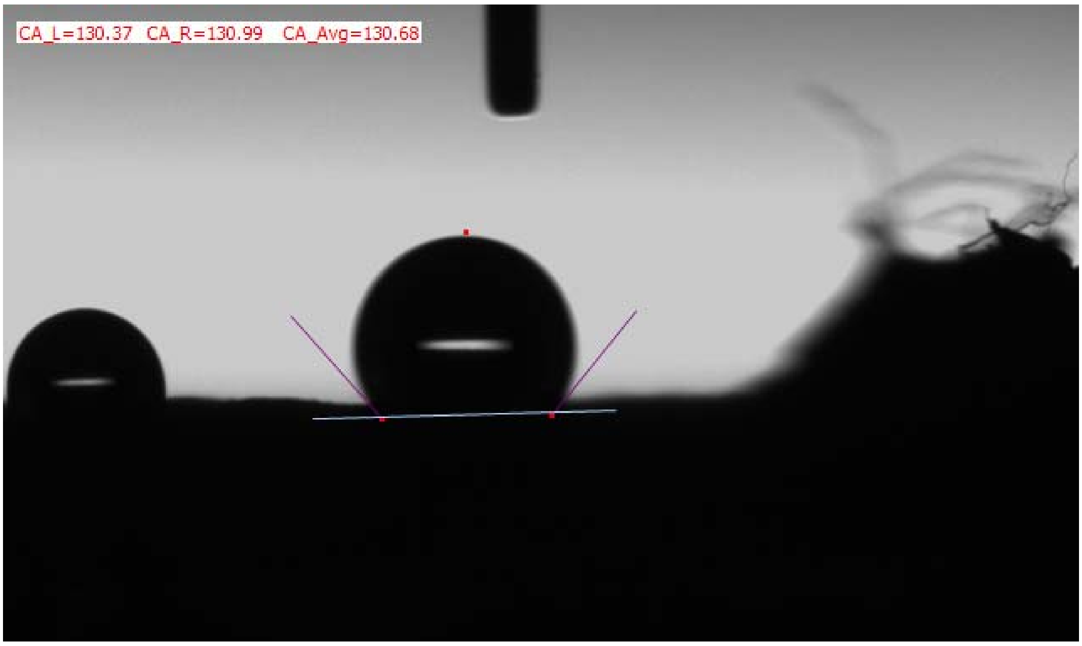
CA_L=130.37 CA_R=130.99 CA_Avg=130.68

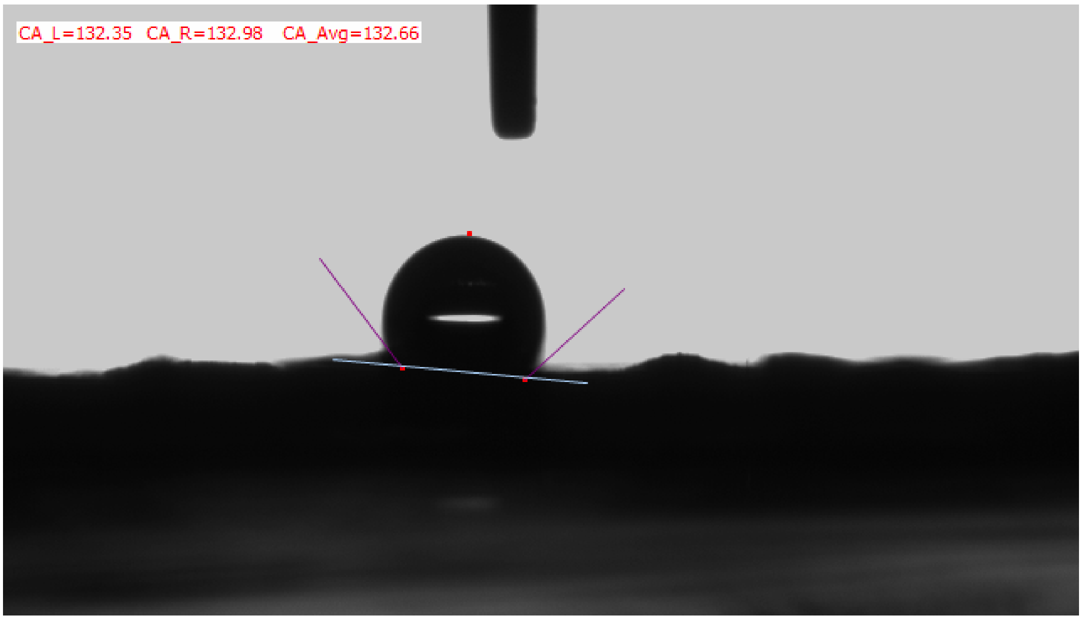
CA_L=132.35 CA_R=132.98 CA_Avg=132.66

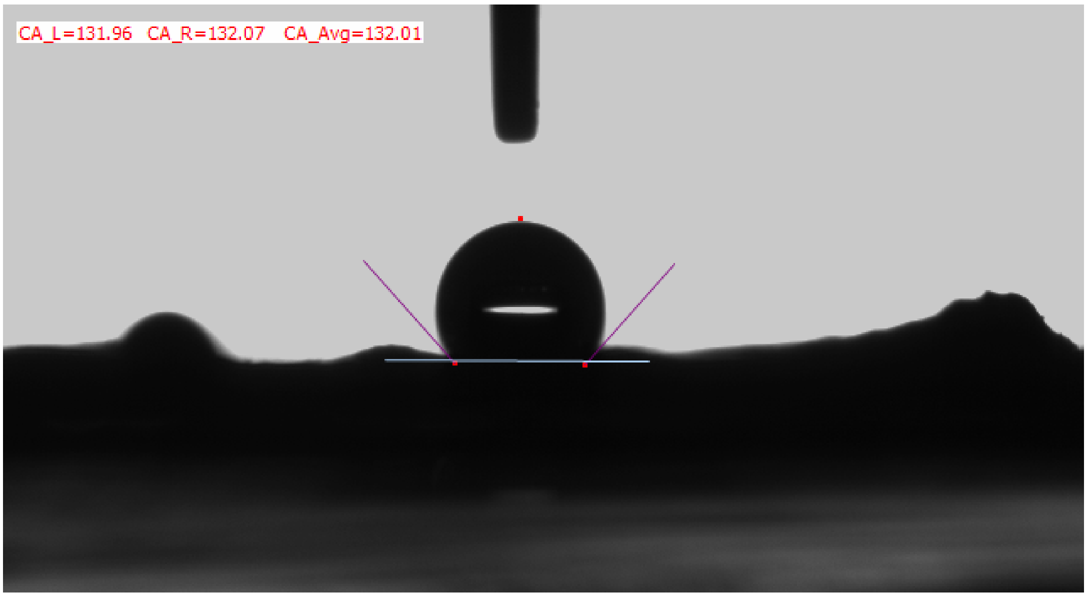
CA_L=131.96 CA_R=132.07 CA_Avg=132.01

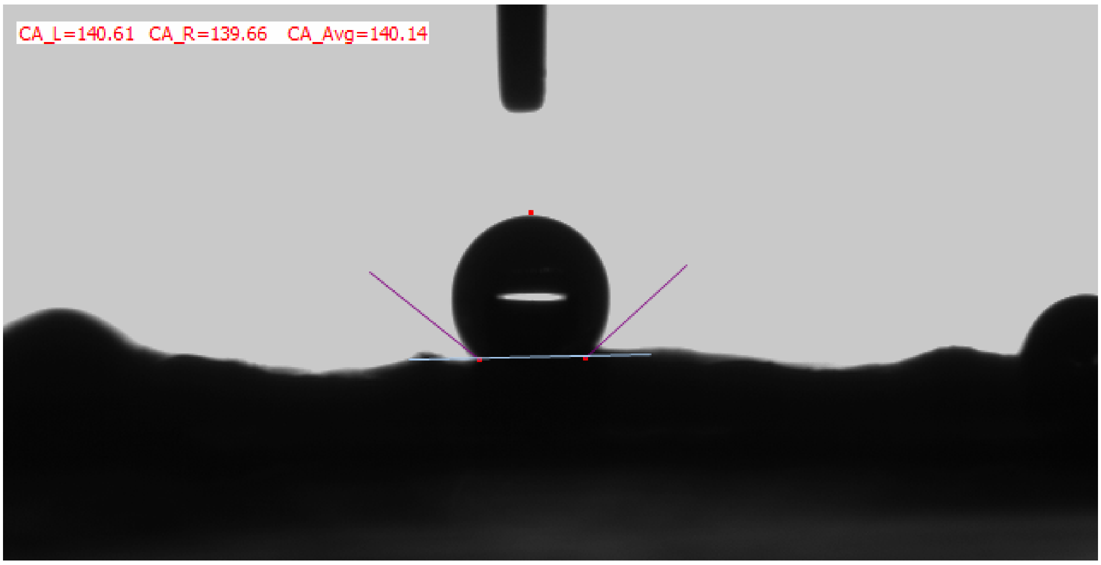
CA_L=140.61 CA_R=139.66 CA_Avg=140.14

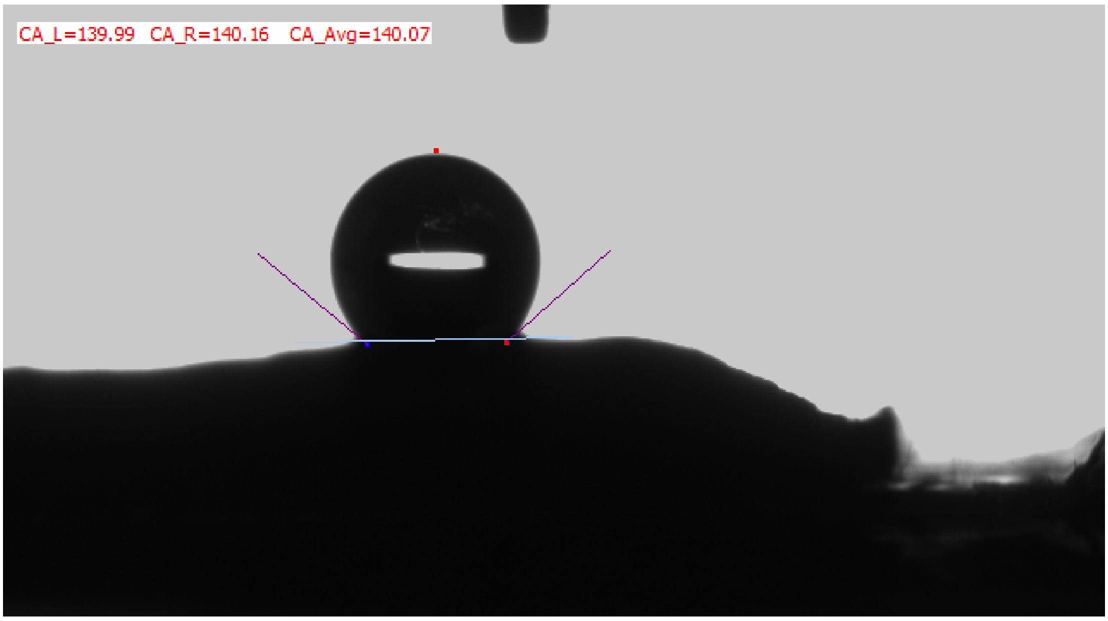
CA_L=139.99 CA_R=140.16 CA_Avg=140.07

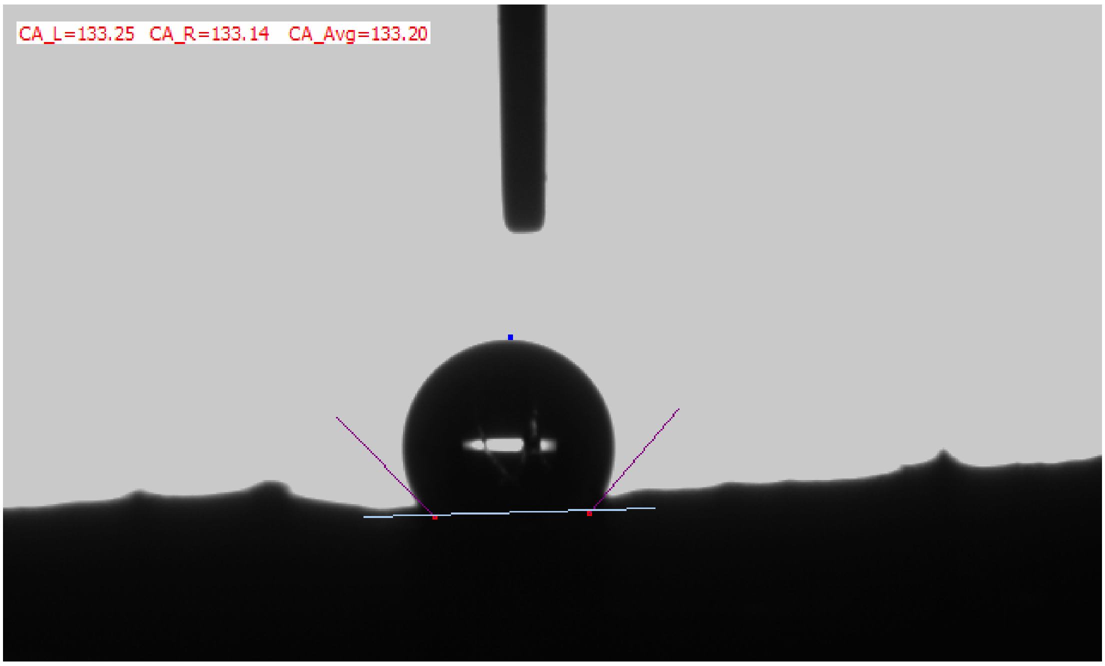
CA_L=133.25 CA_R=133.14 CA_Avg=133.20

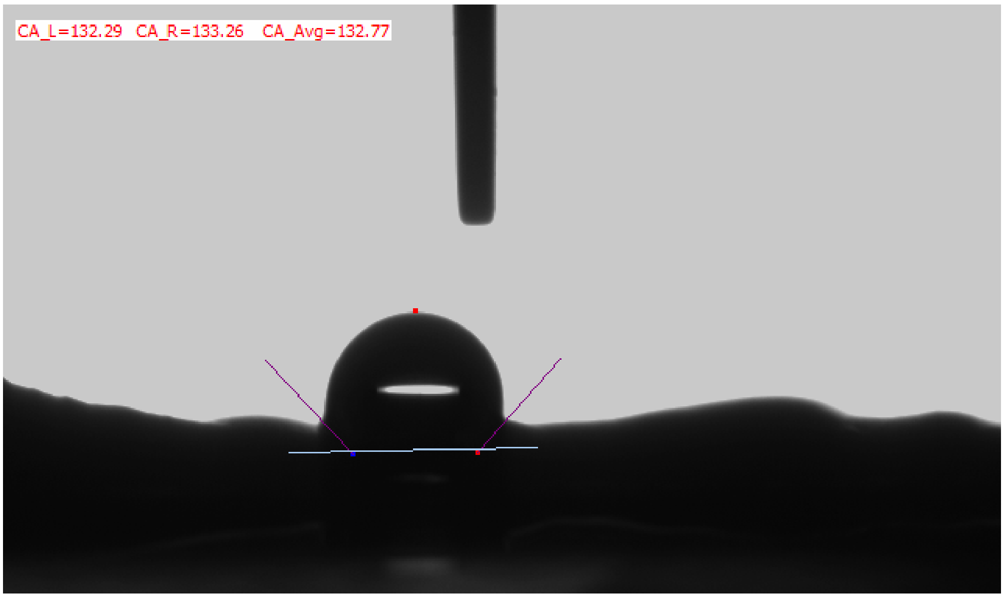
CA_L=132.29 CA_R=133.26 CA_Avg=132.77

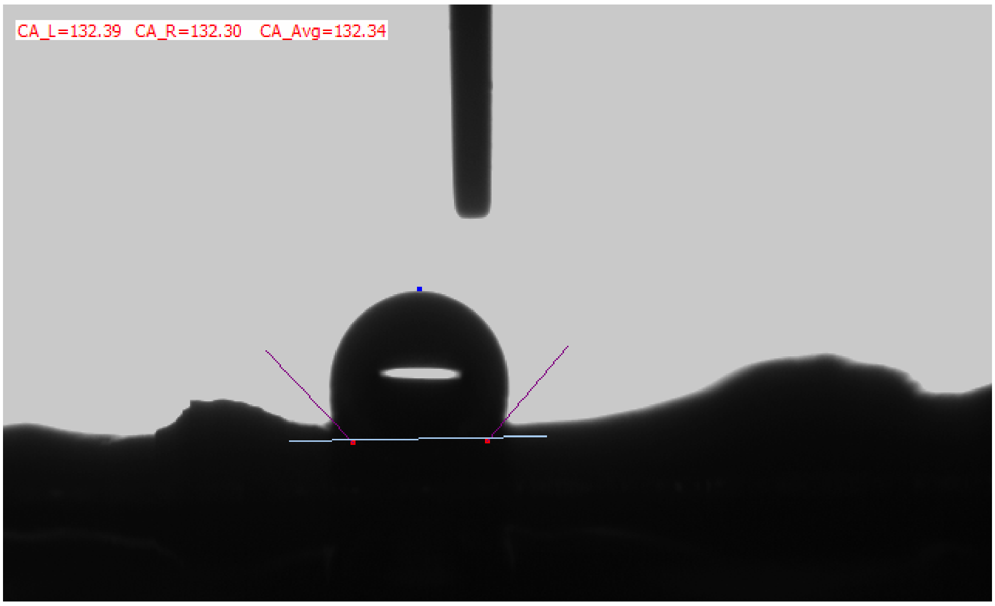
CA_L=132.39 CA_R=132.30 CA_Avg=132.34

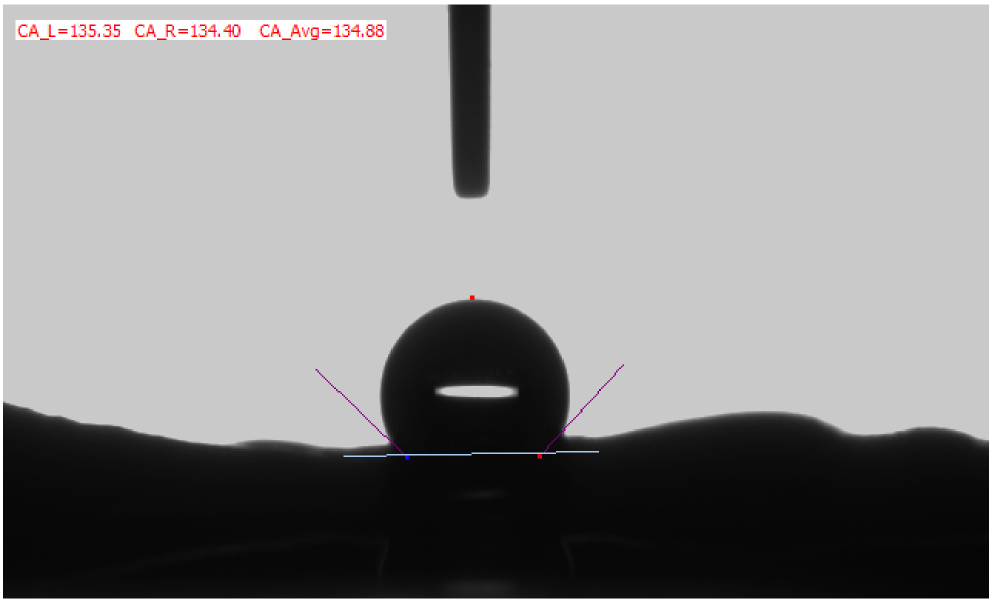
CA_L=135.35 CA_R=134.40 CA_Avg=134.88

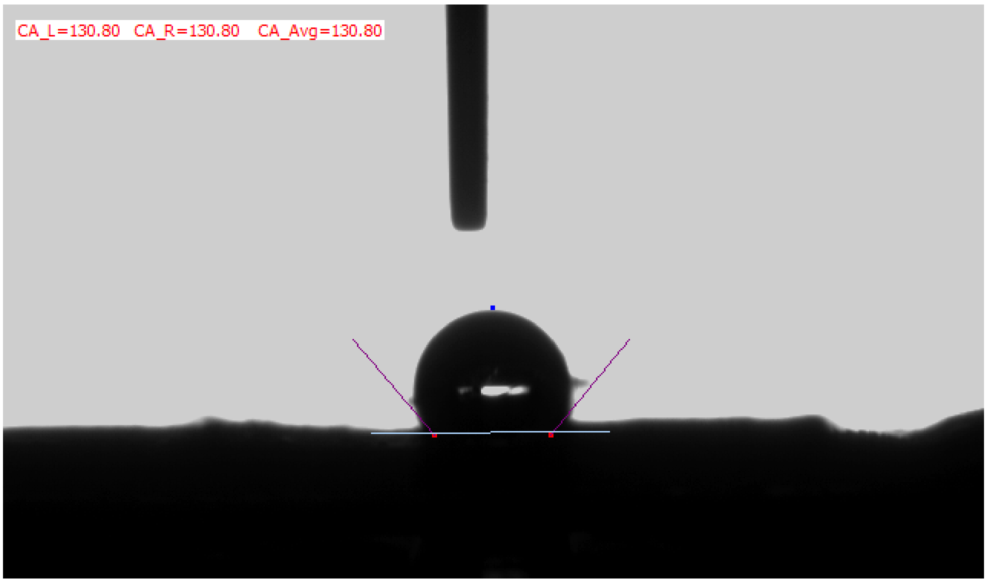

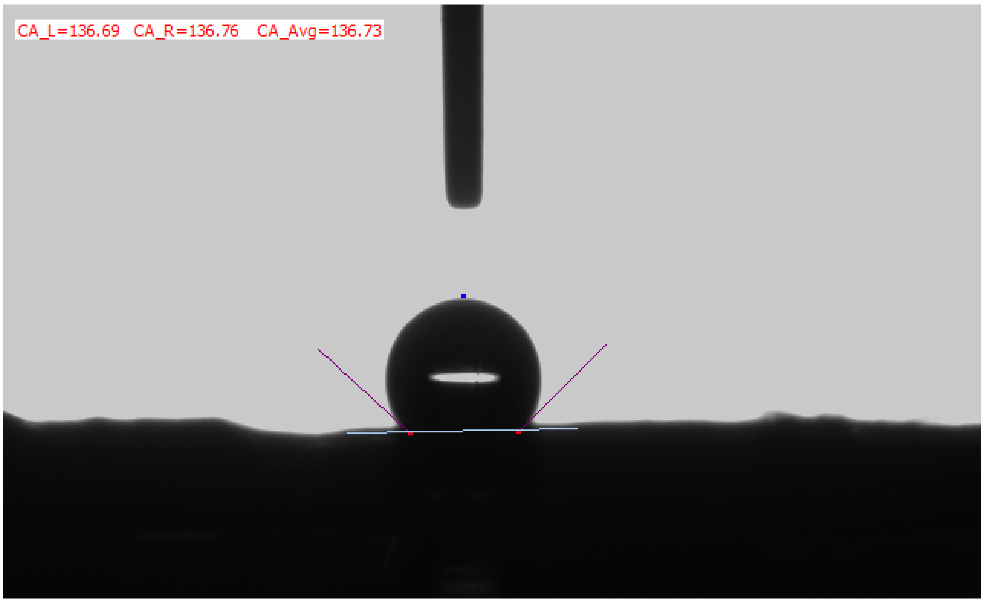

N. dyeriana **(16 images)**

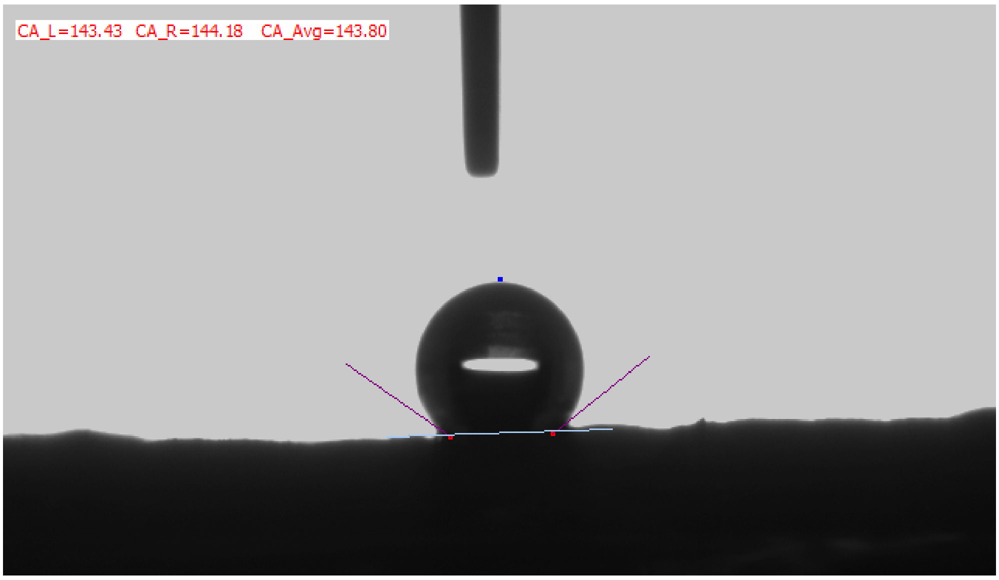

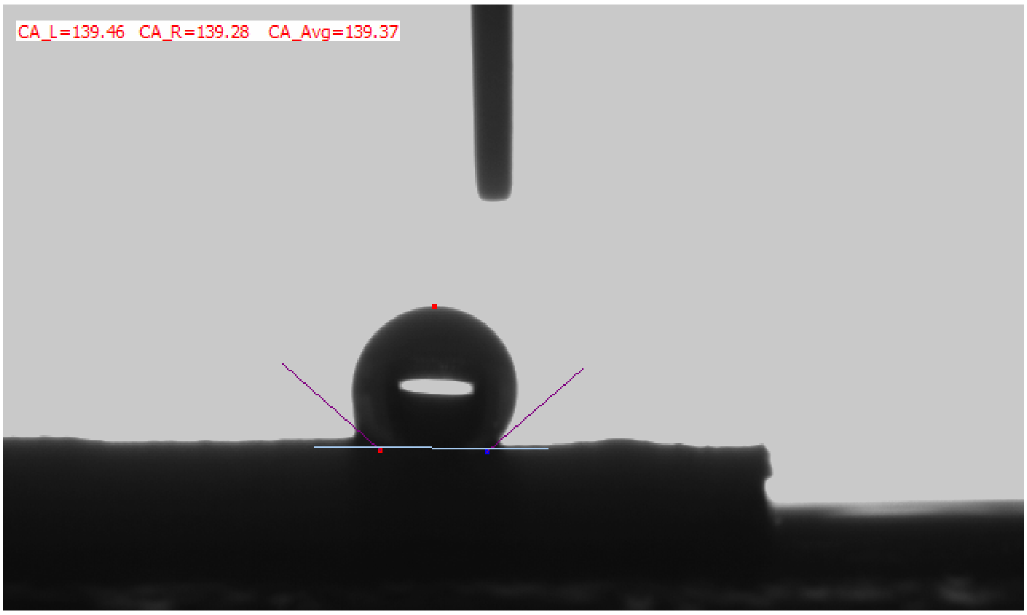
CA_L=139.46 CA_R=139.28 CA_Avg=139.37
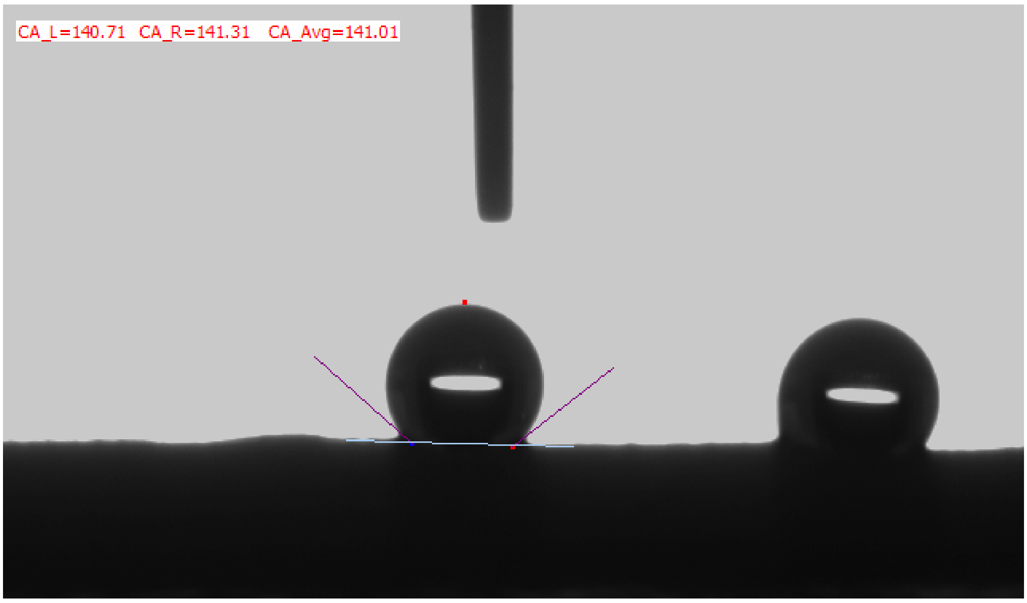
CA_L=140.71 CA_R=141.31 CA_Avg=141.01
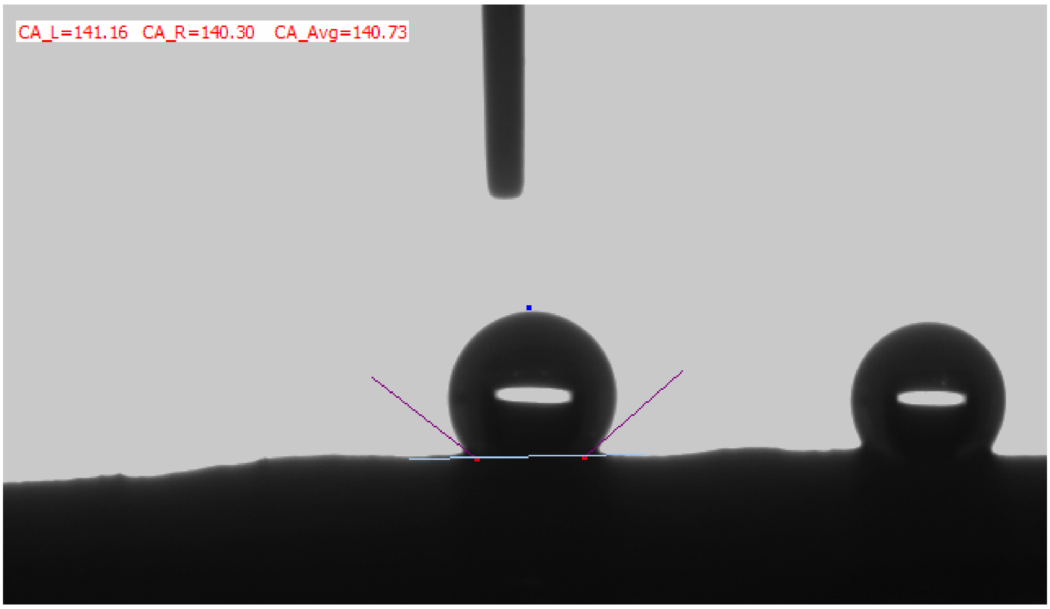
CA_L=141.16 CA_R=140.30 CA_Avg=140.73

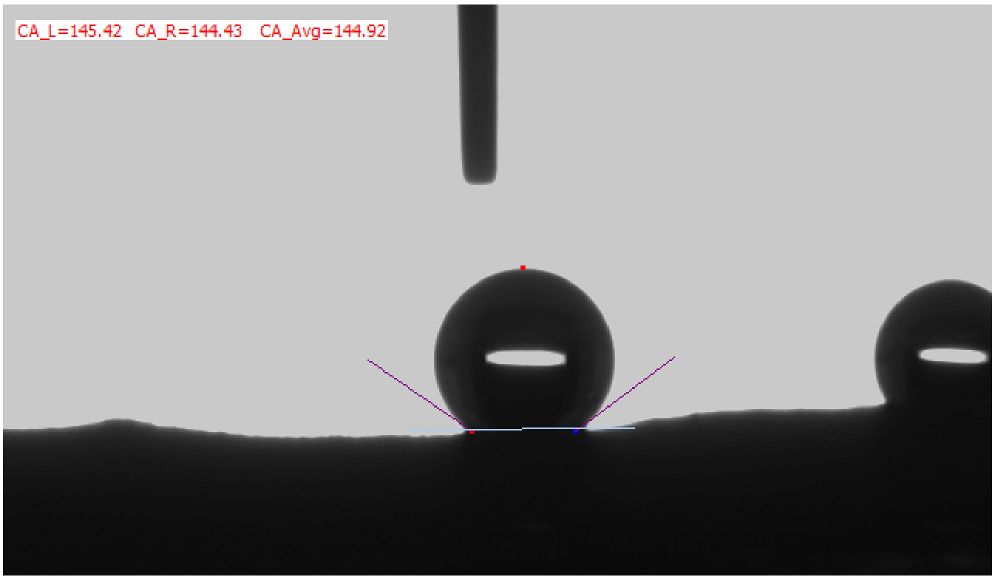
CA_L=145.42 CA_R=144.43 CA_Avg=144.92

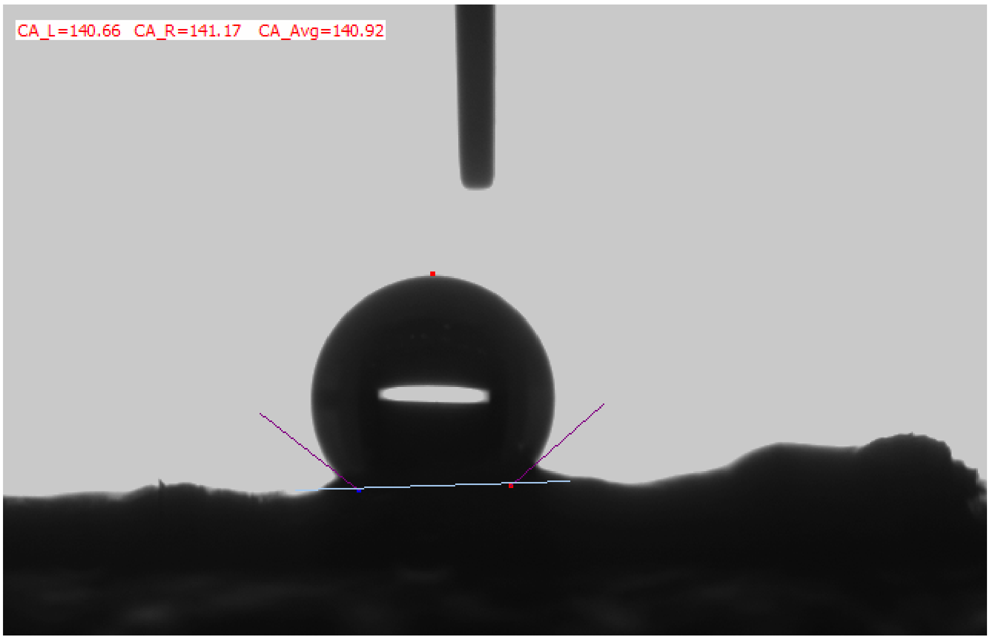
CA_L=140.66 CA_R=141.17 CA_Avg=140.92

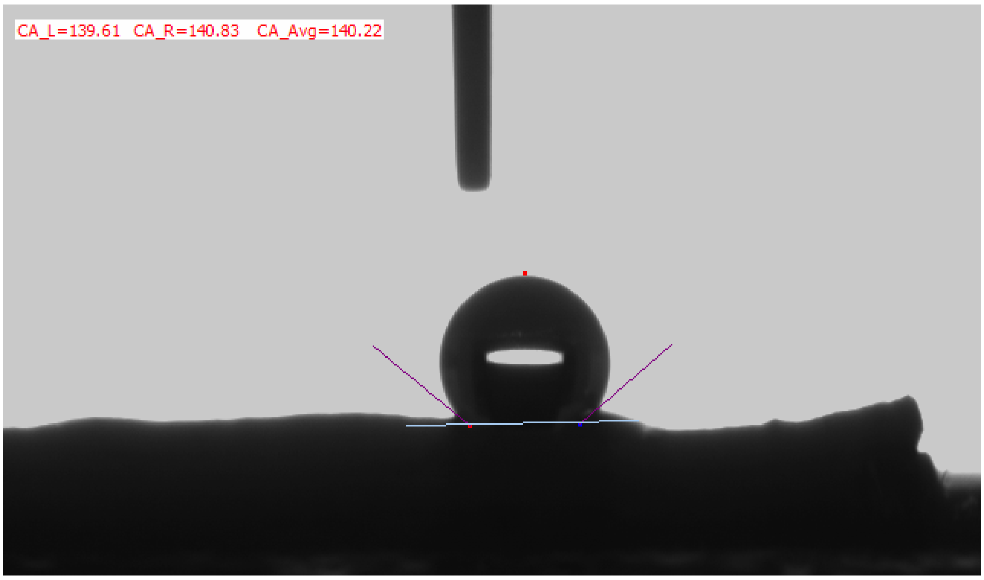
CA_L=139.61 CA_R=140.83 CA_Avg=140.22

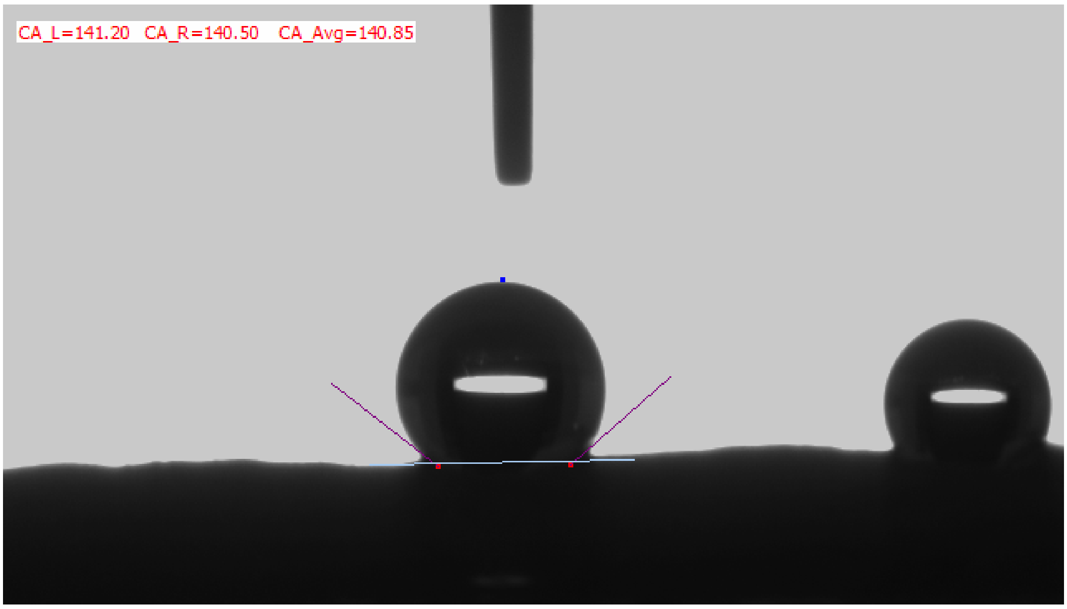
CA_L=141.20 CA_R=140.50 CA_Avg=140.85

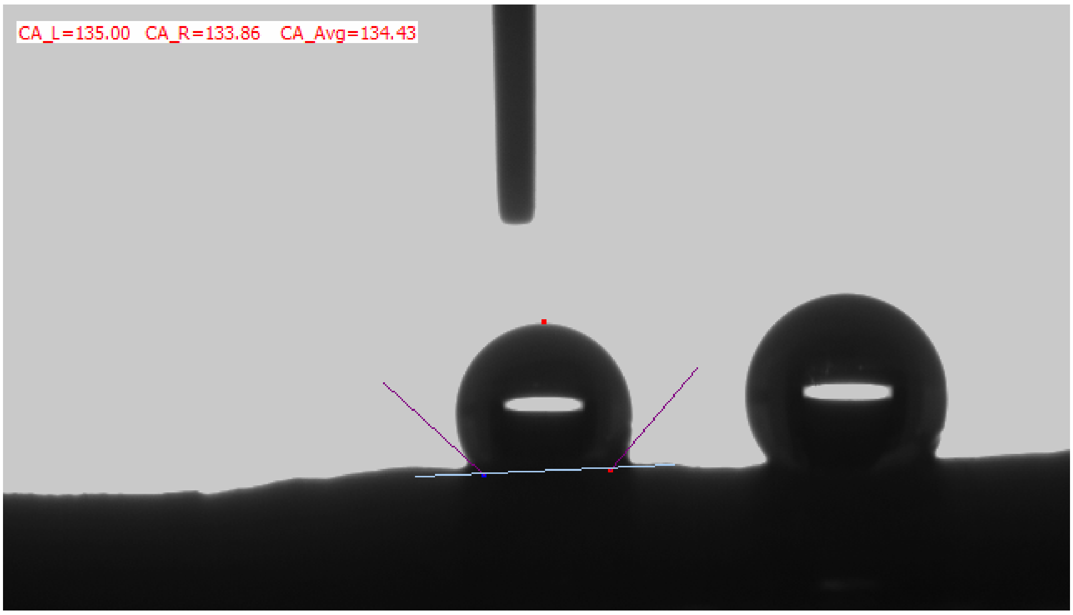
CA_L=135.00 CA_R=133.86 CA_Avg=134.43

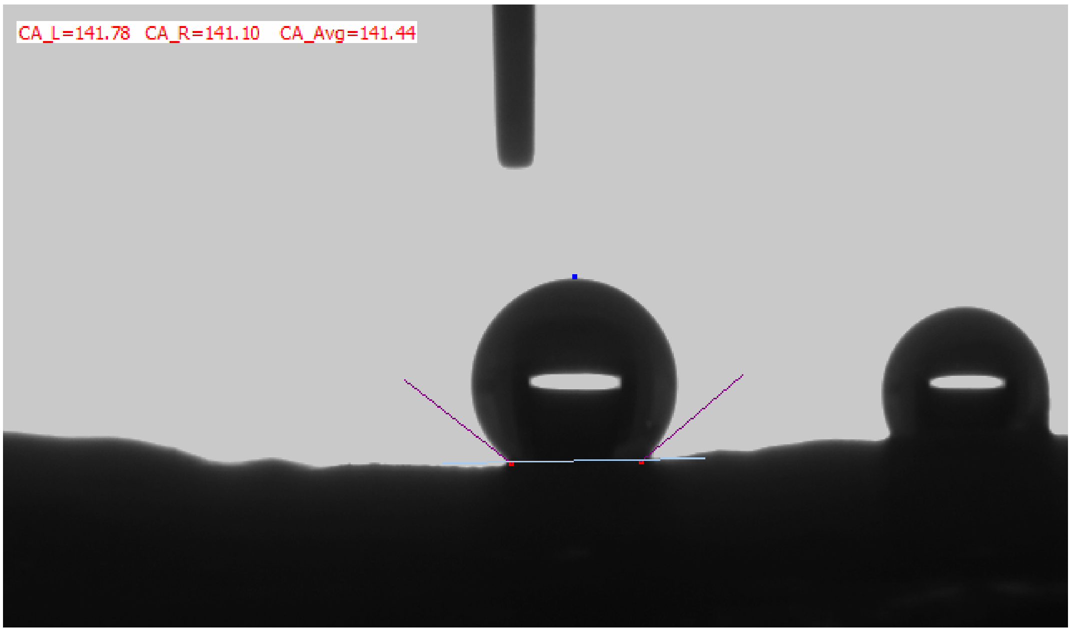
CA_L=141.78 CA_R=141.10 CA_Avg=141.44

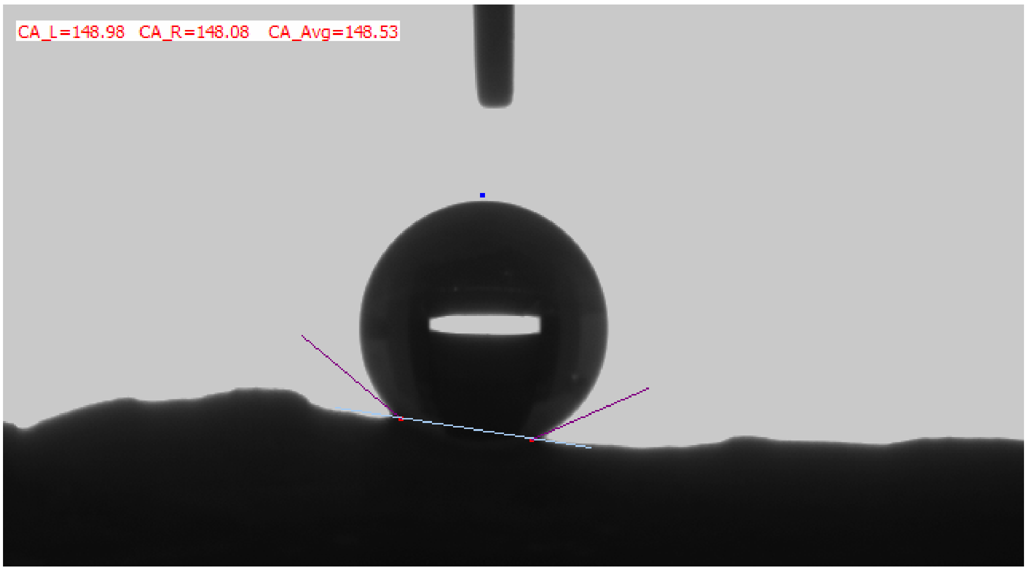
CA_L=148.98 CA_R=148.08 CA_Avg=148.53

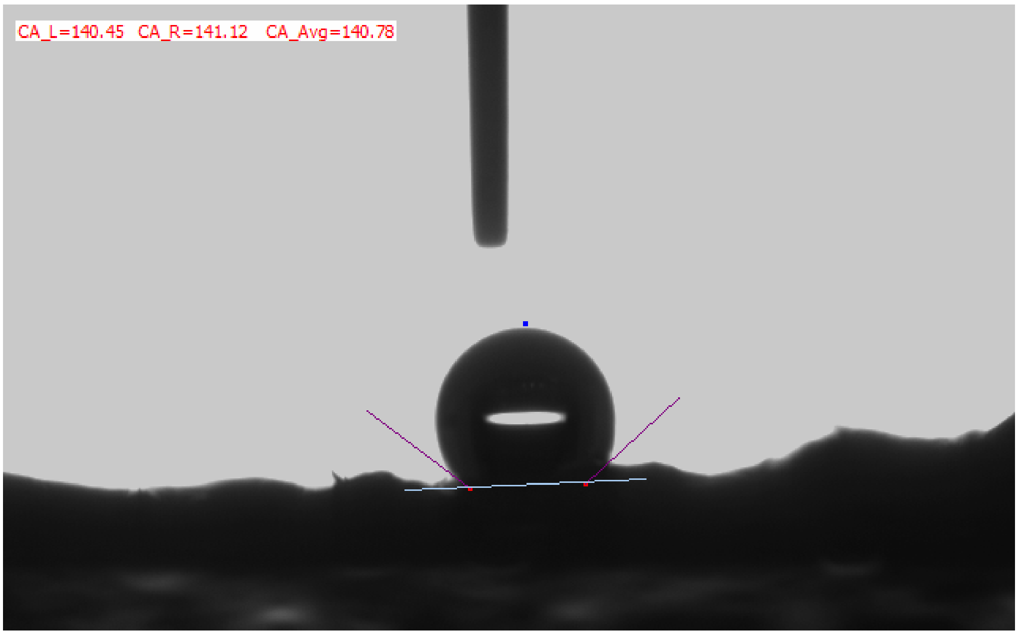
CA_L=140.45 CA_R=141.12 CA_Avg=140.78

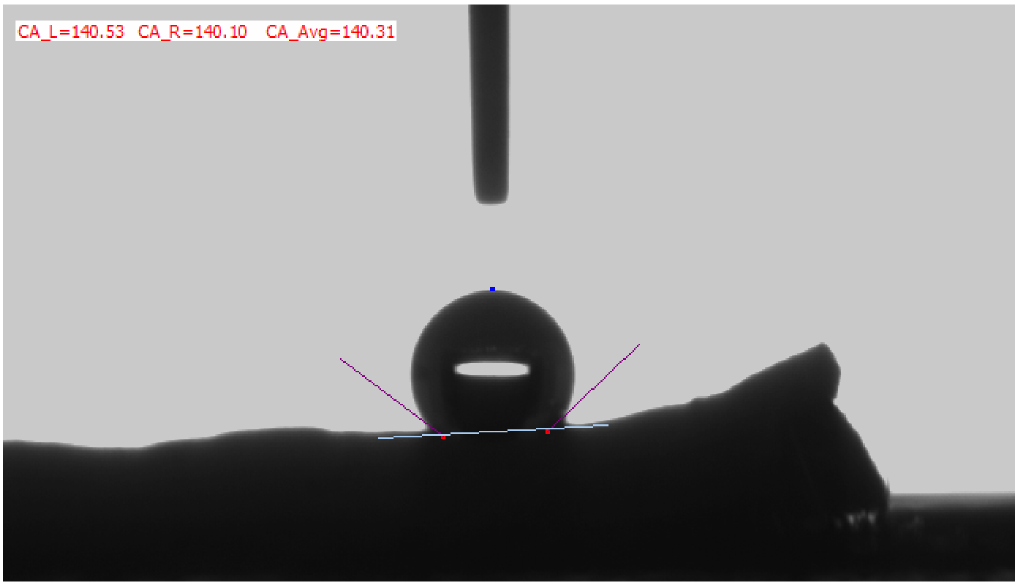
CA_L=140.53 CA_R=140.10 CA_Avg=140.31

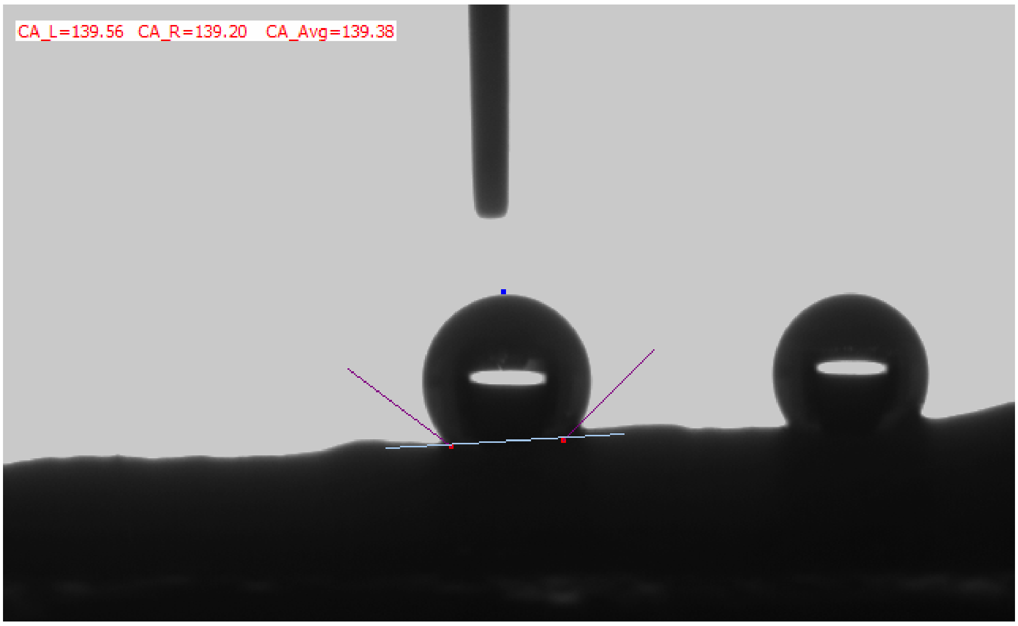
CA_L=139.56 CA_R=139.20 CA_Avg=139.38

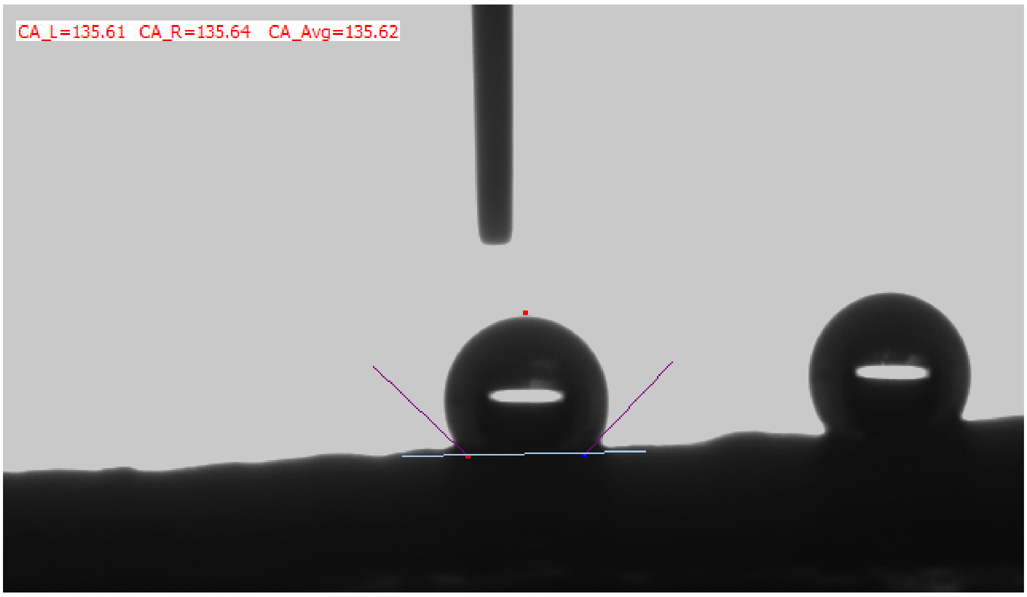
CA_L=135.61 CA_R=135.64 CA_Avg=135.62

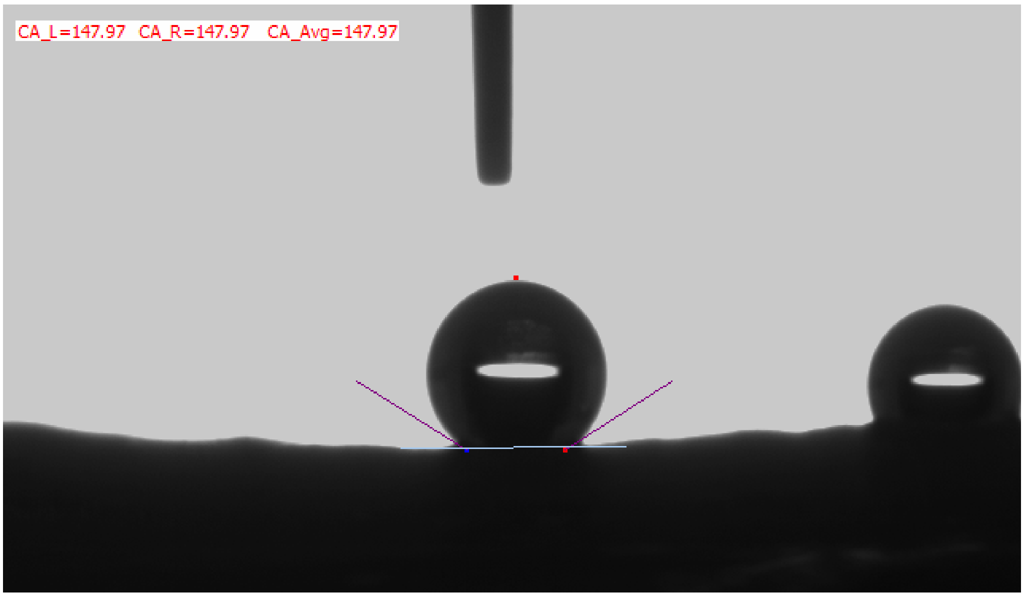
CA_L=147.97 CA_R=147.97 CA_Avg=147.97

N. fusca **(15 images)**

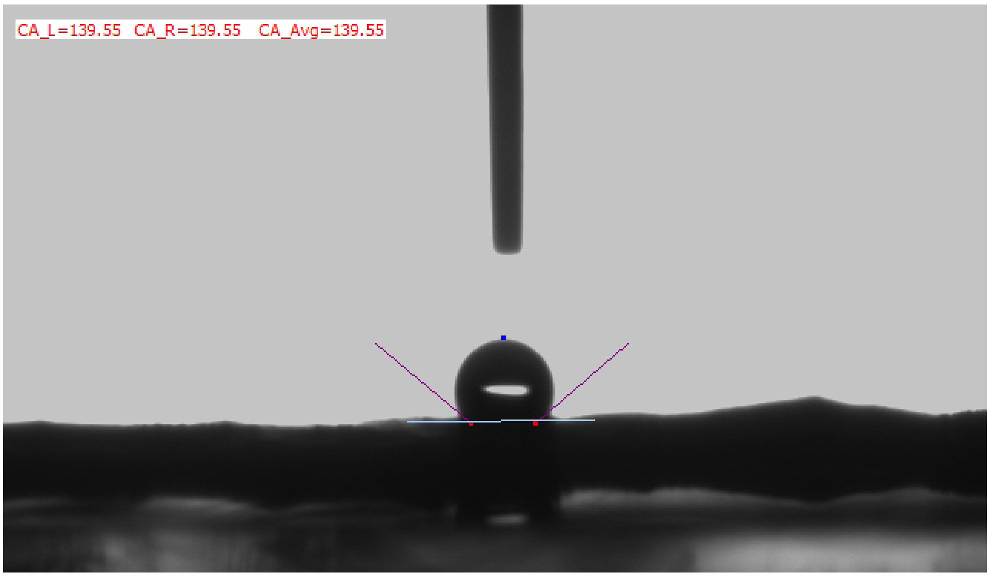

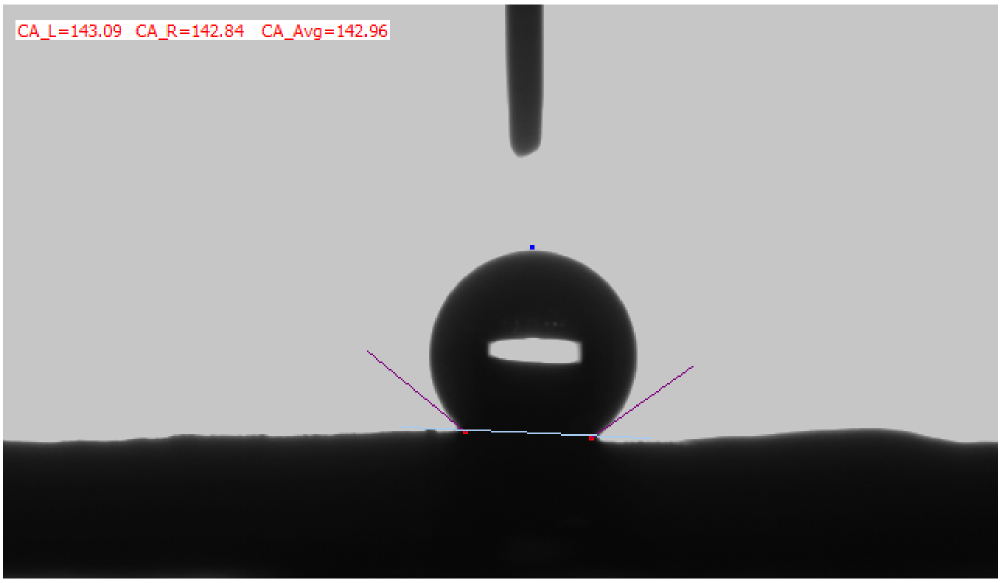

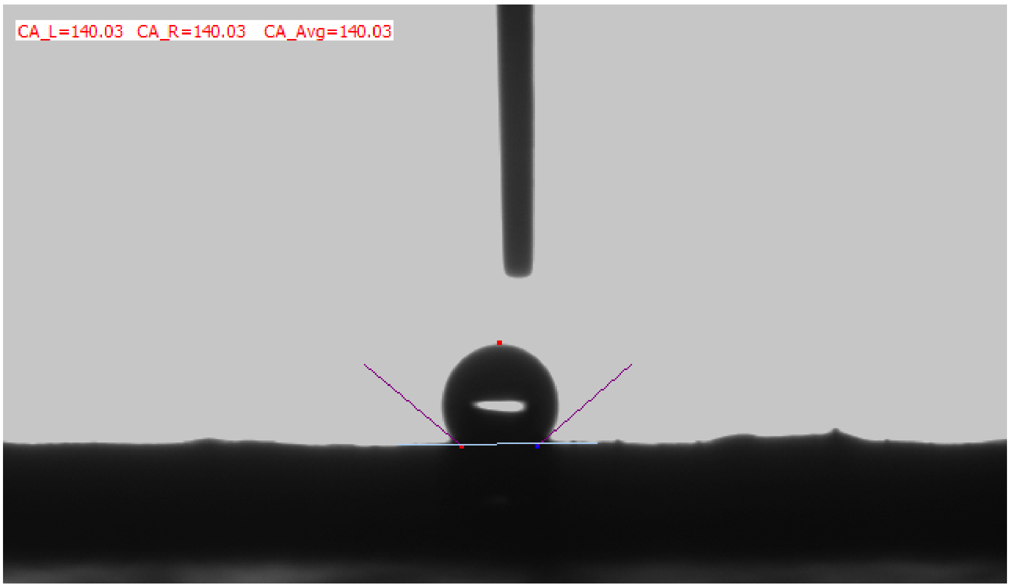

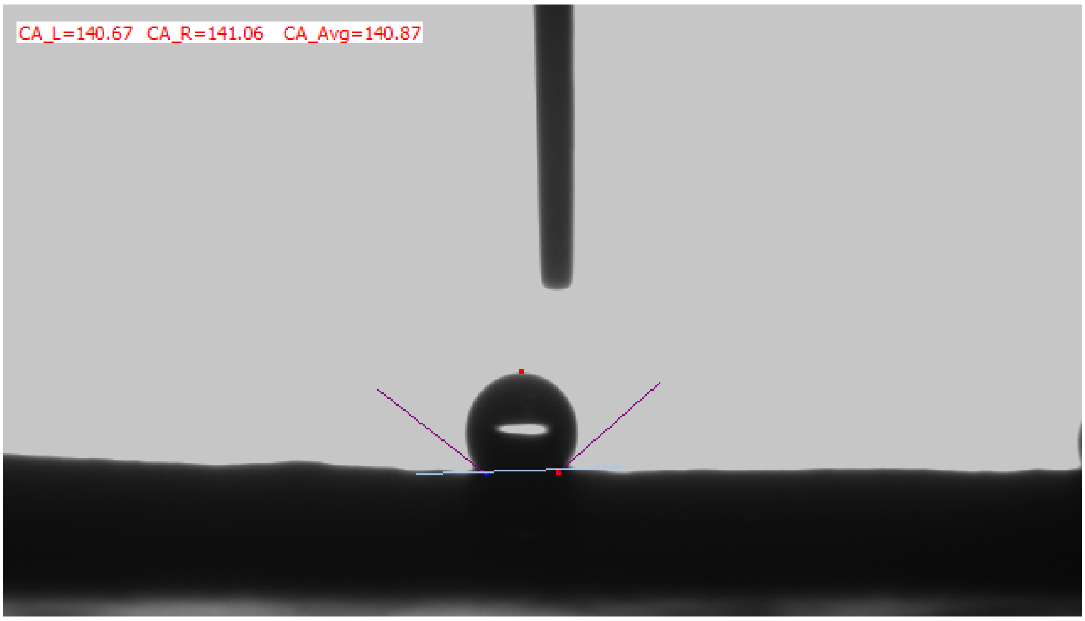
CA_L=140.67 CA_R=141.06 CA_Avg=140.87

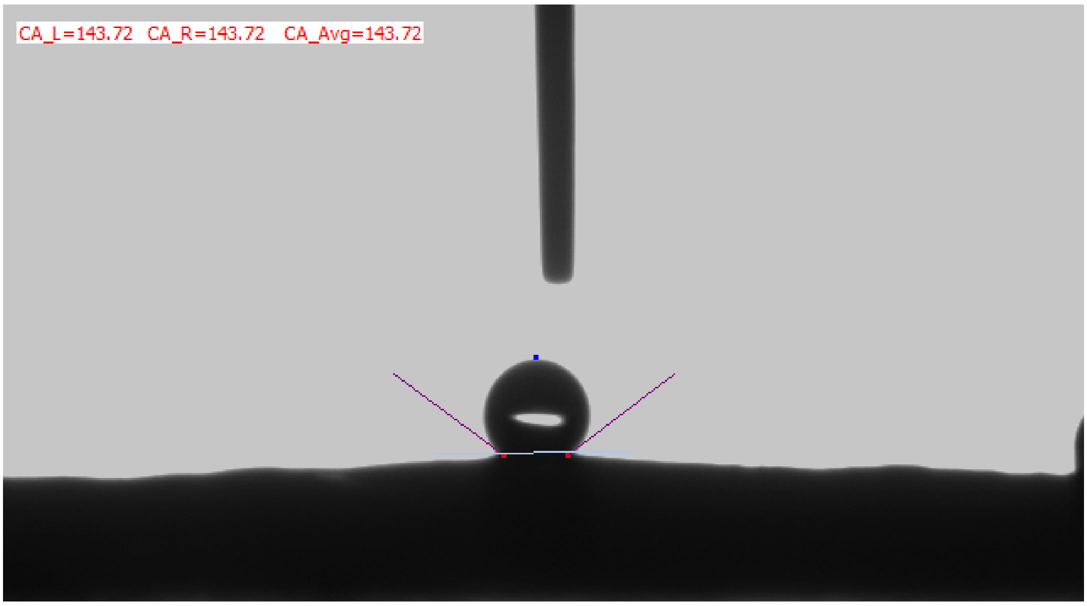
CA_L=143.72 CA_R=143.72 CA_Avg=143.72

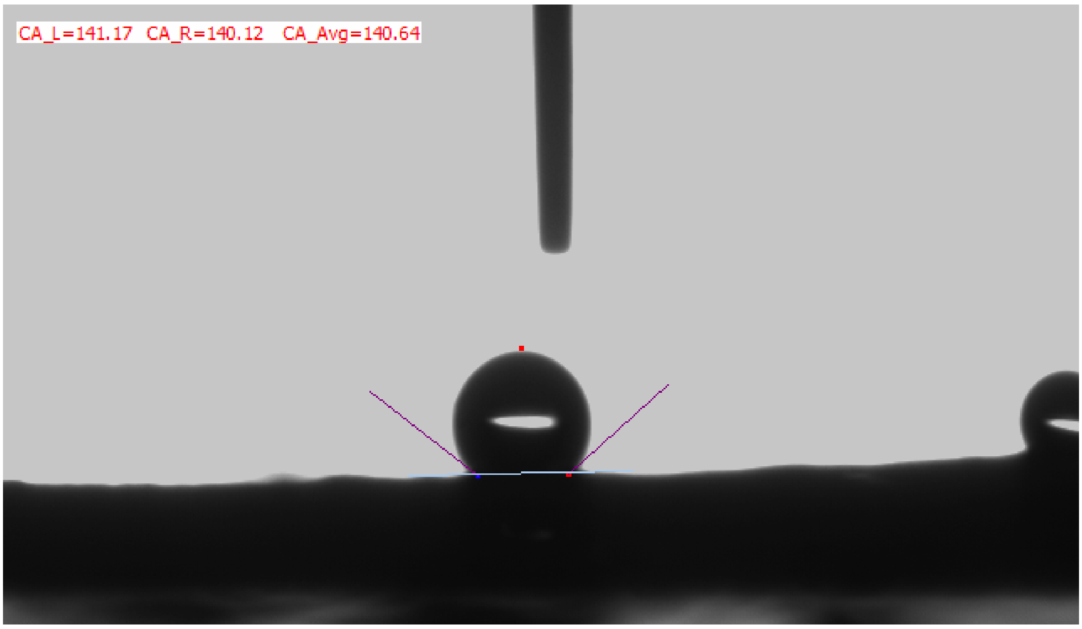
CA_L=141.17 CA_R=140.12 CA_Avg=140.64

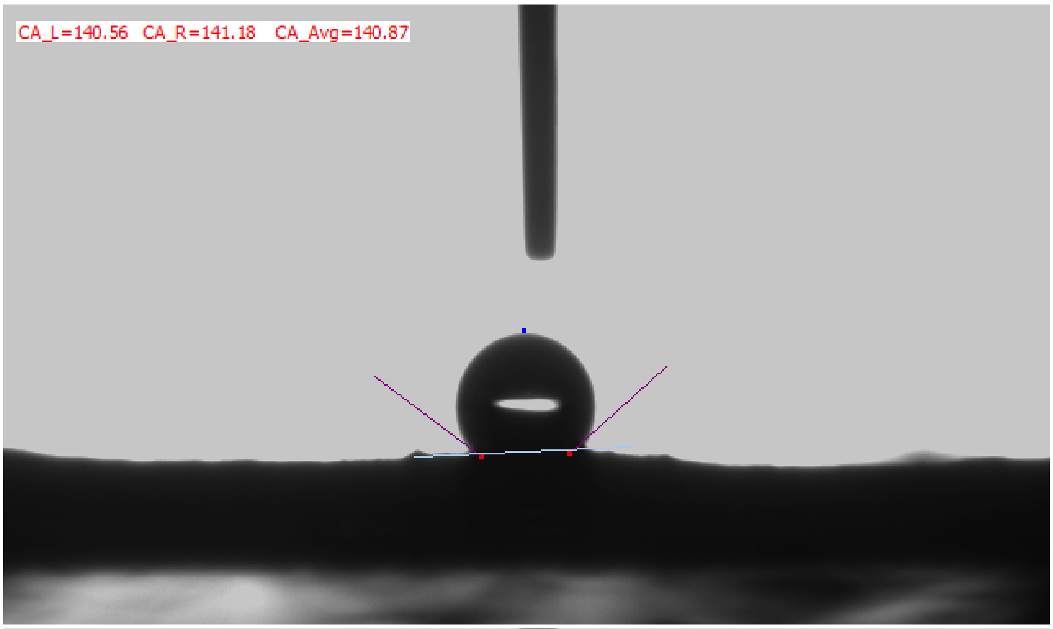
CA_L=140.56 CA_R=141.18 CA_Avg=140.87

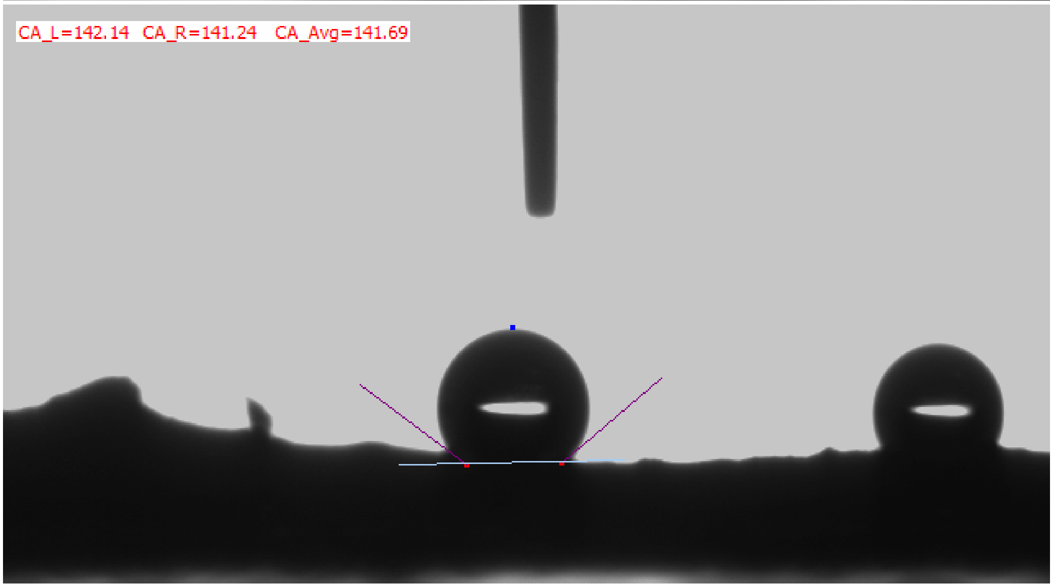
CA_L=142.14 CA_R=141.24 CA_Avg=141.69

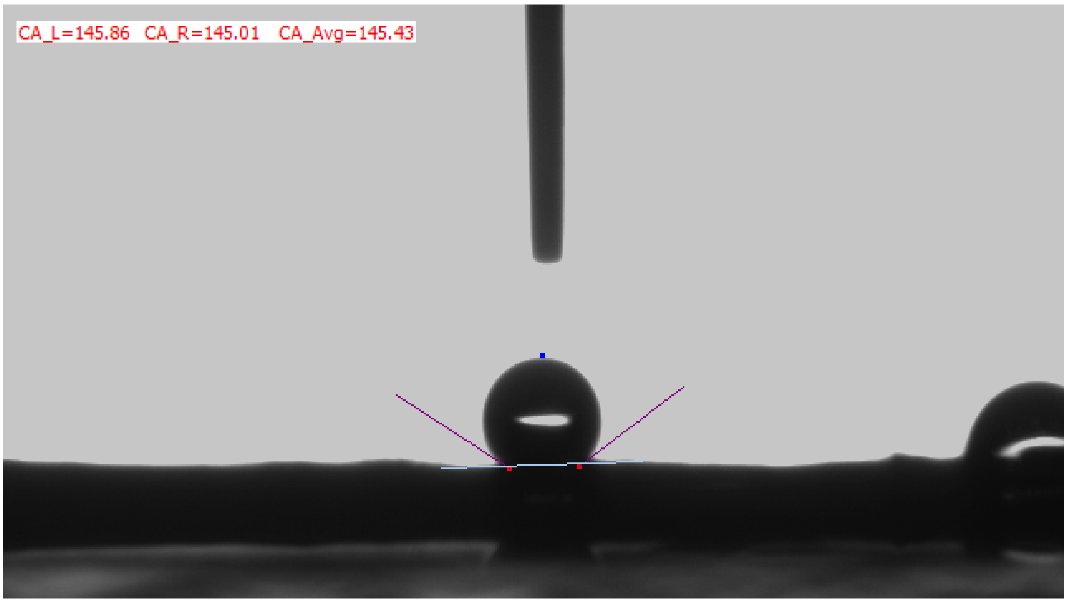
CA_L=145.86 CA_R=145.01 CA_Avg=145.43

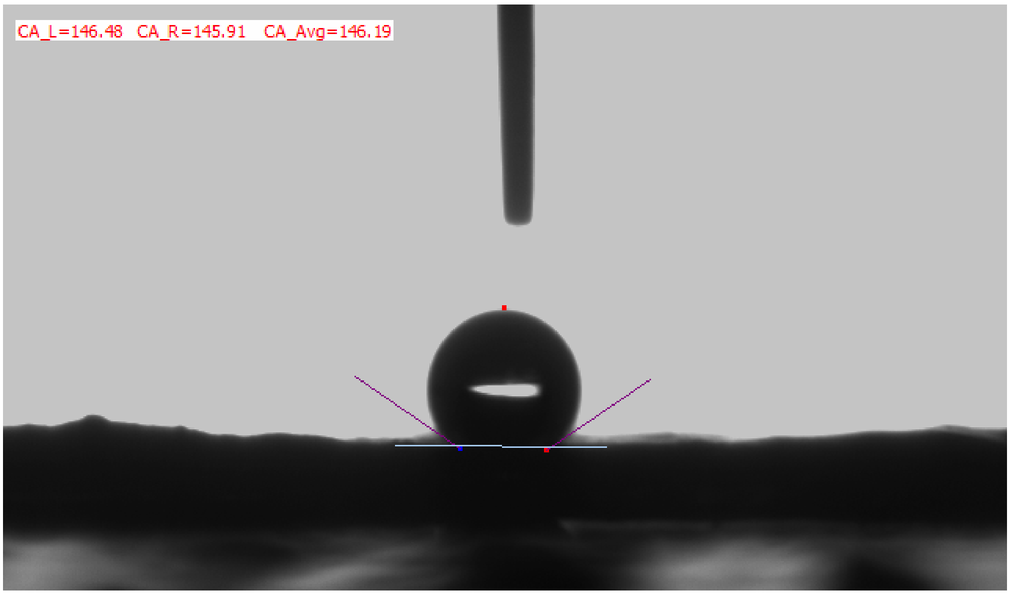
CA_L=146.48 CA_R=145.91 CA_Avg=146.19

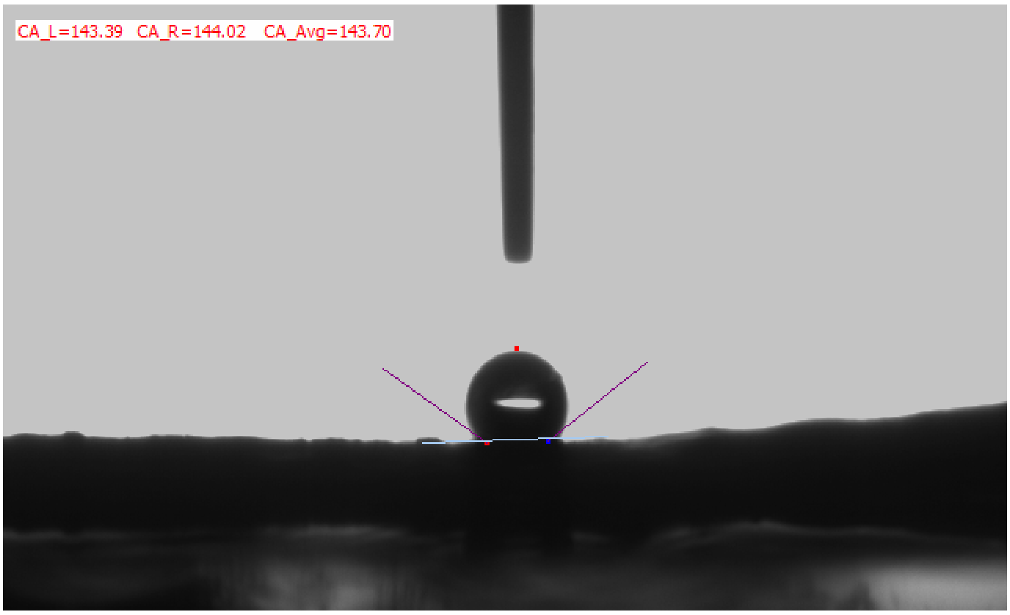
CA_L=143.39 CA_R=144.02 CA_Avg=143.70

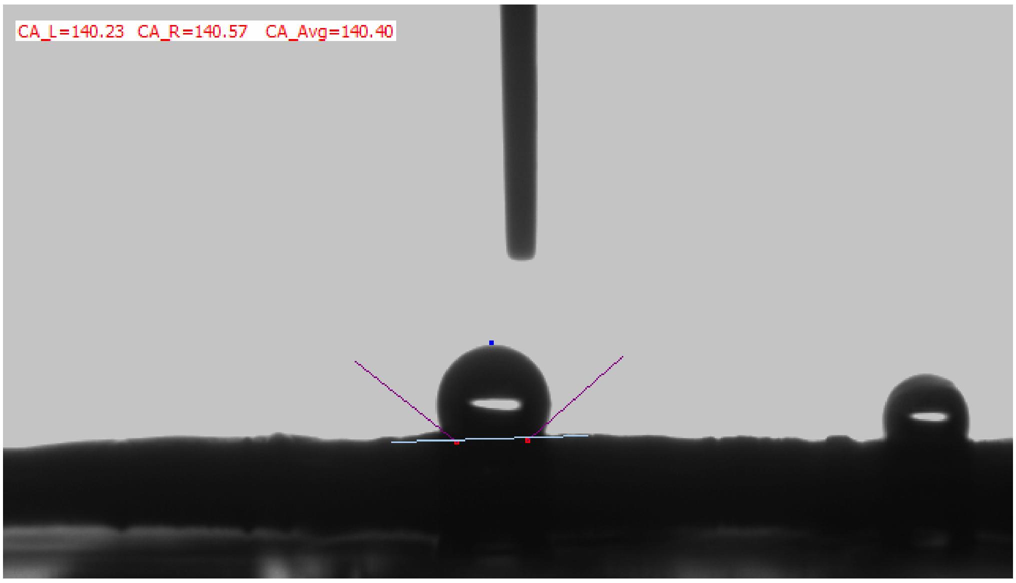
CA_L=140.23 CA_R=140.57 CA_Avg=140.40

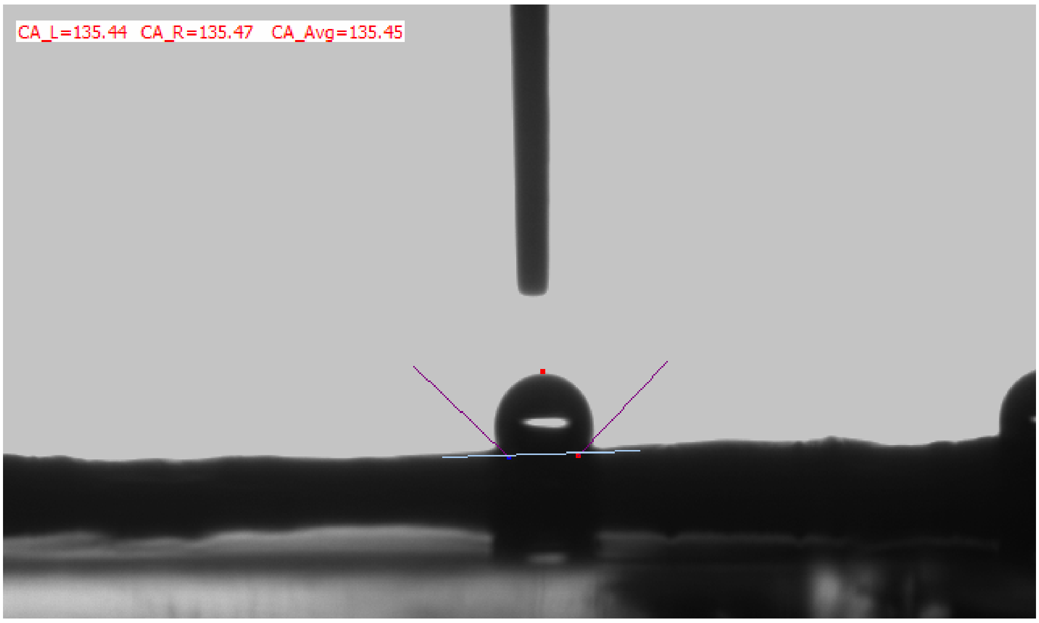
CA_L=135.44 CA_R=135.47 CA_Avg=135.45

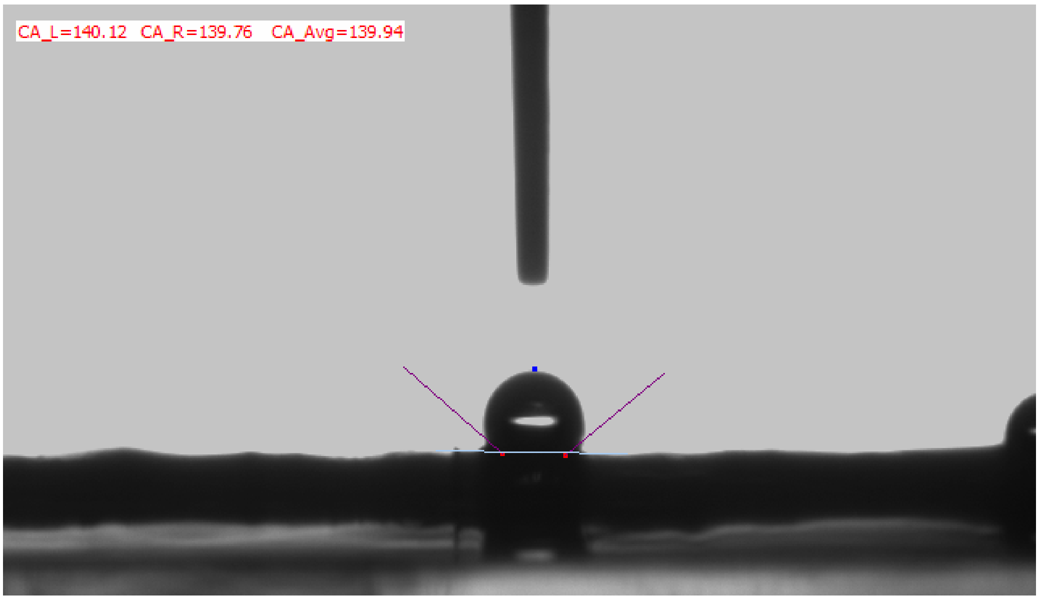
CA_L=140.12 CA_R=139.76 CA_Avg=139.94

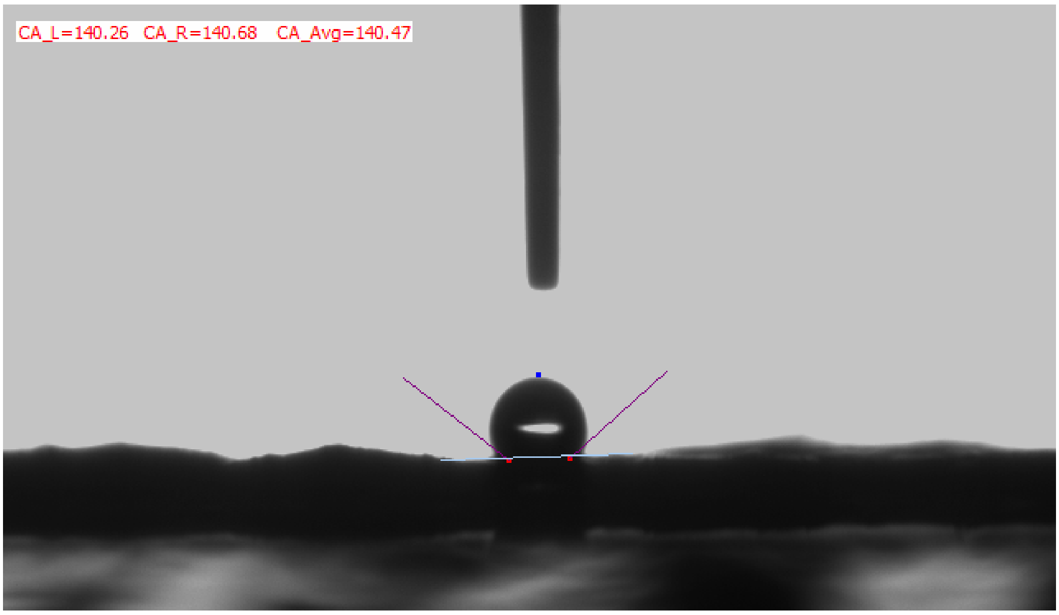
CA_L=140.26 CA_R=140.68 CA_Avg=140.47

N. gracillima **(12 images)**

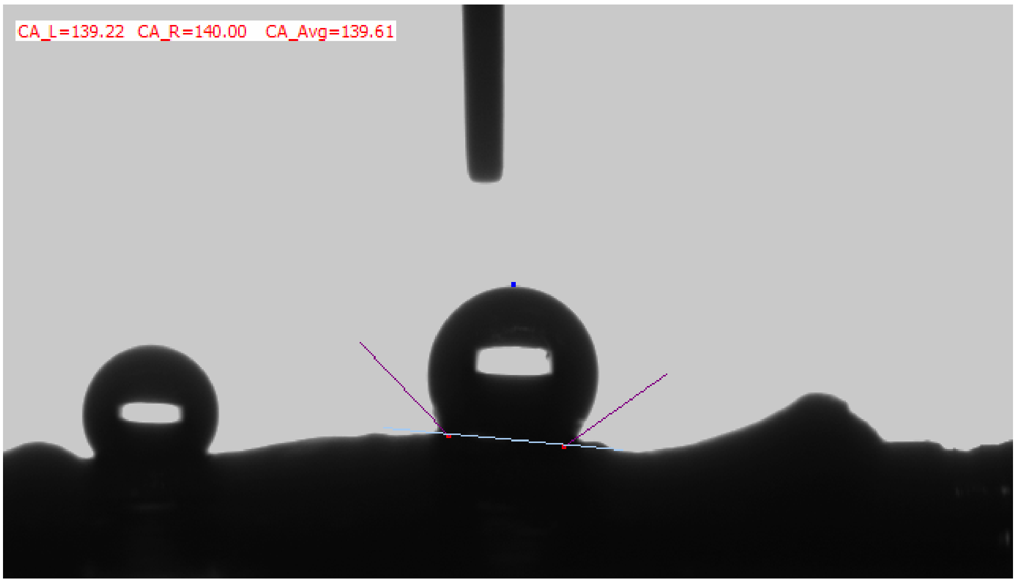

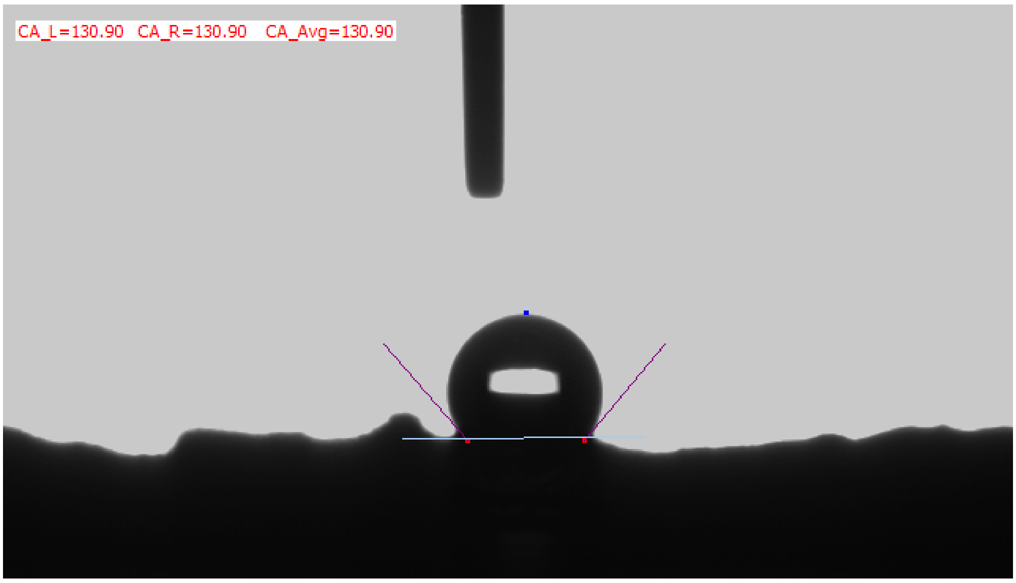

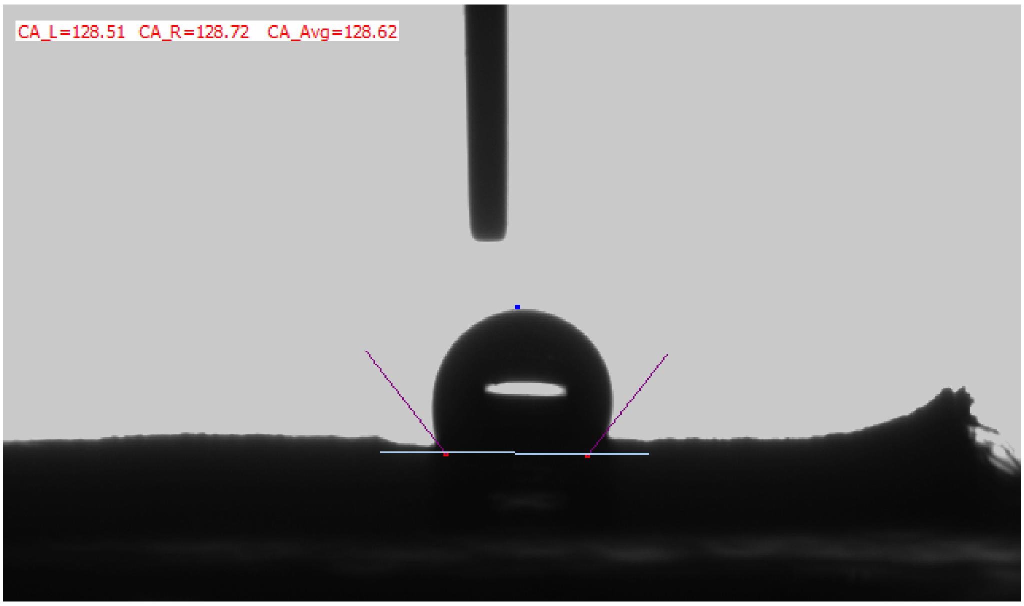

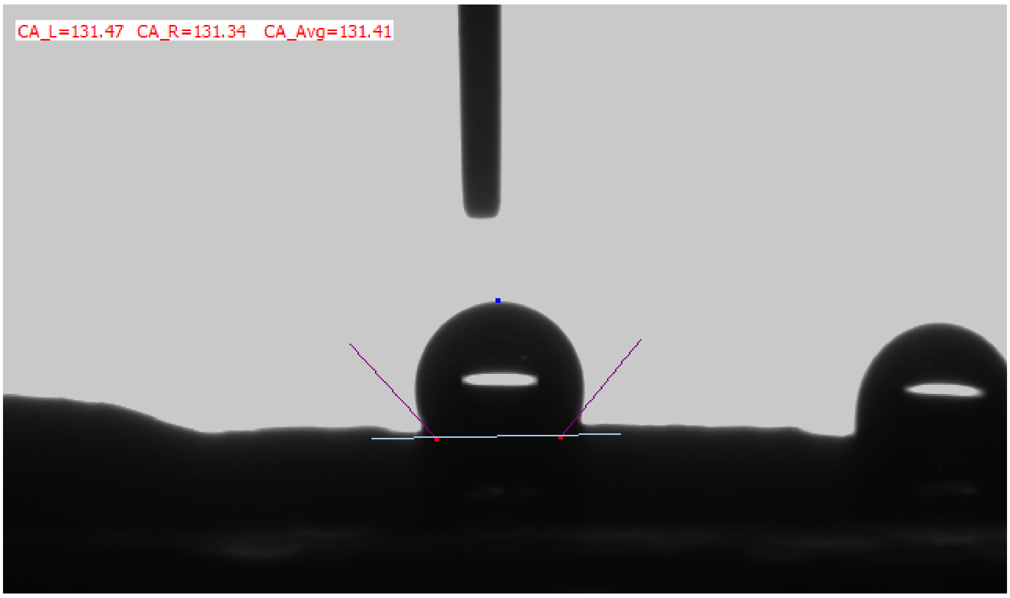
CA_L=131.47 CA_R=131.34 CA_Avg=131.41

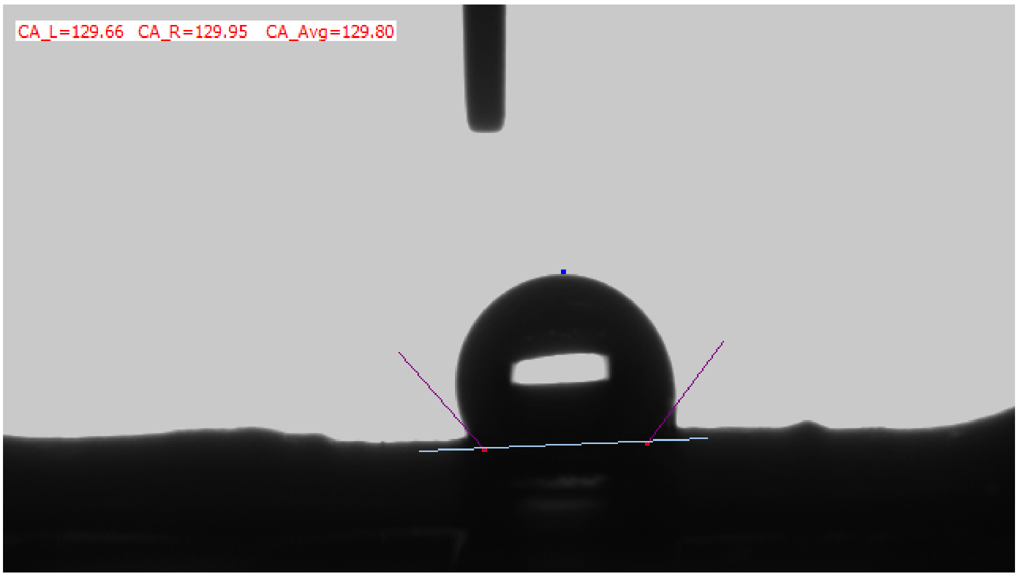
CA_L=129.66 CA_R=129.95 CA_Avg=129.80

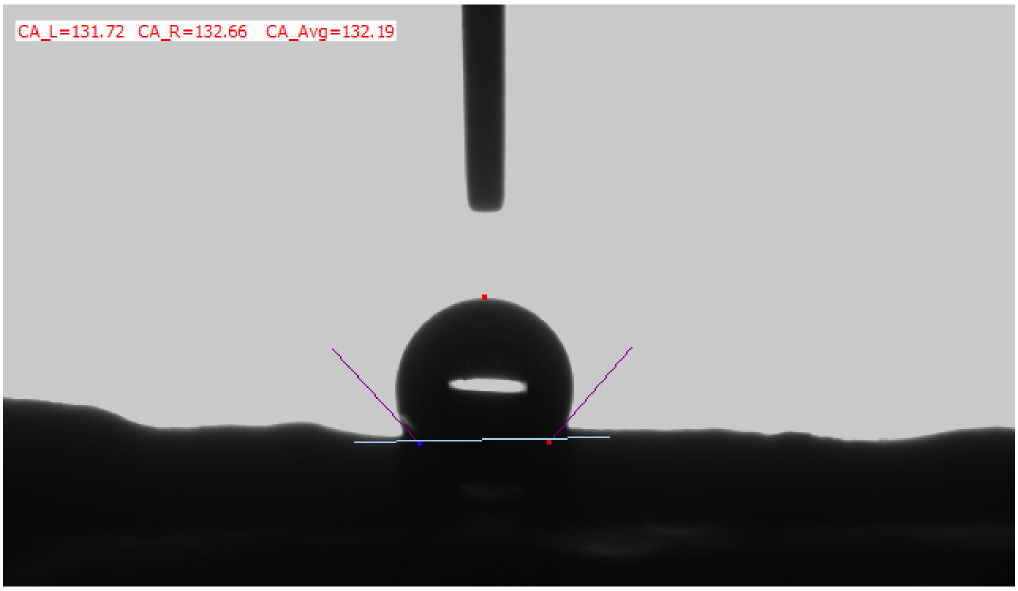
CA_L=131.72 CA_R=132.66 CA_Avg=132.19

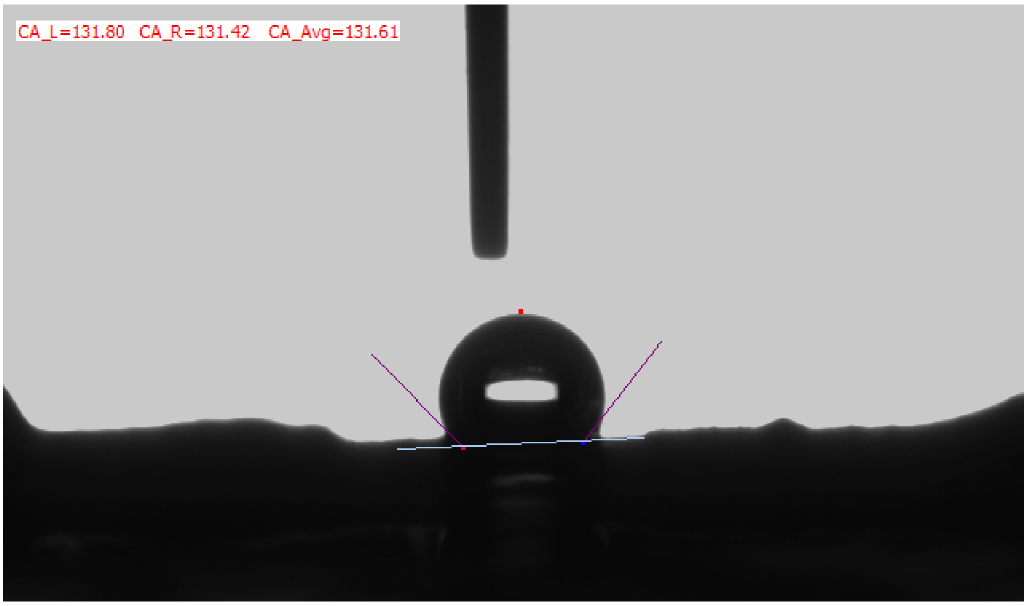
CA_L=131.80 CA_R=131.42 CA_Avg=131.61

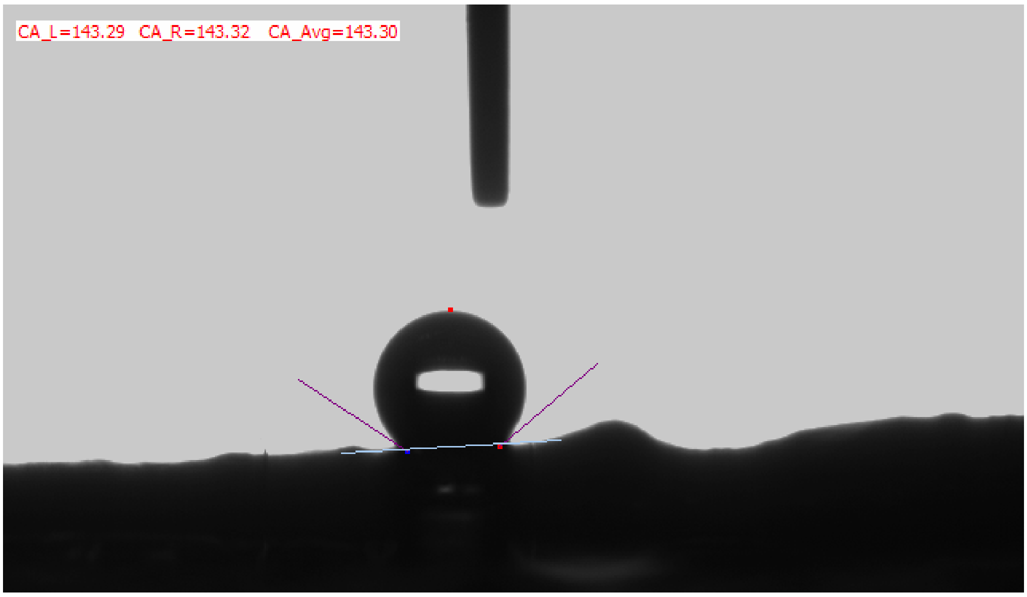
CA_L=143.29 CA_R=143.32 CA_Avg=143.30

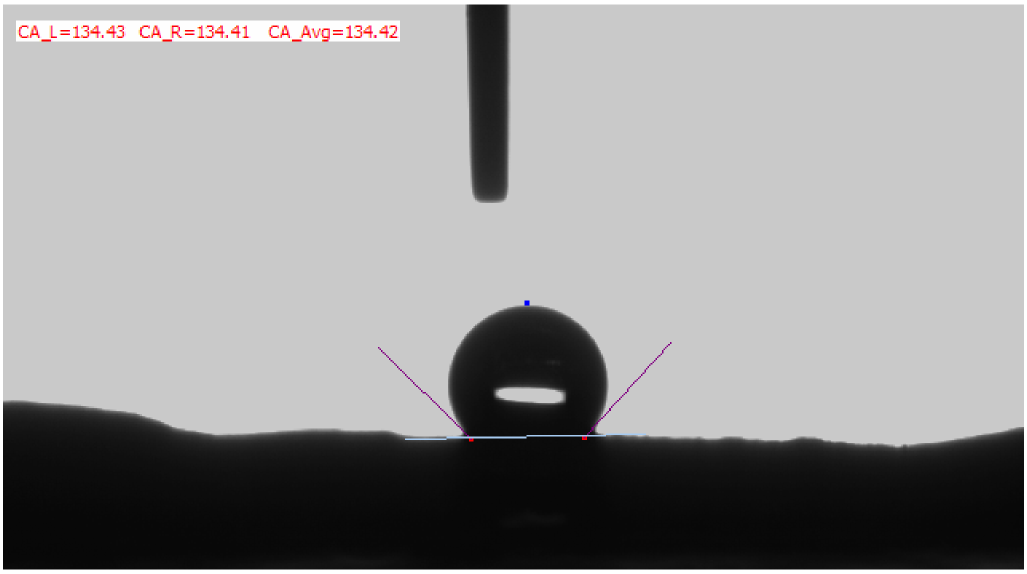
CA_L=134.43 CA_R=134.41 CA_Avg=134.42

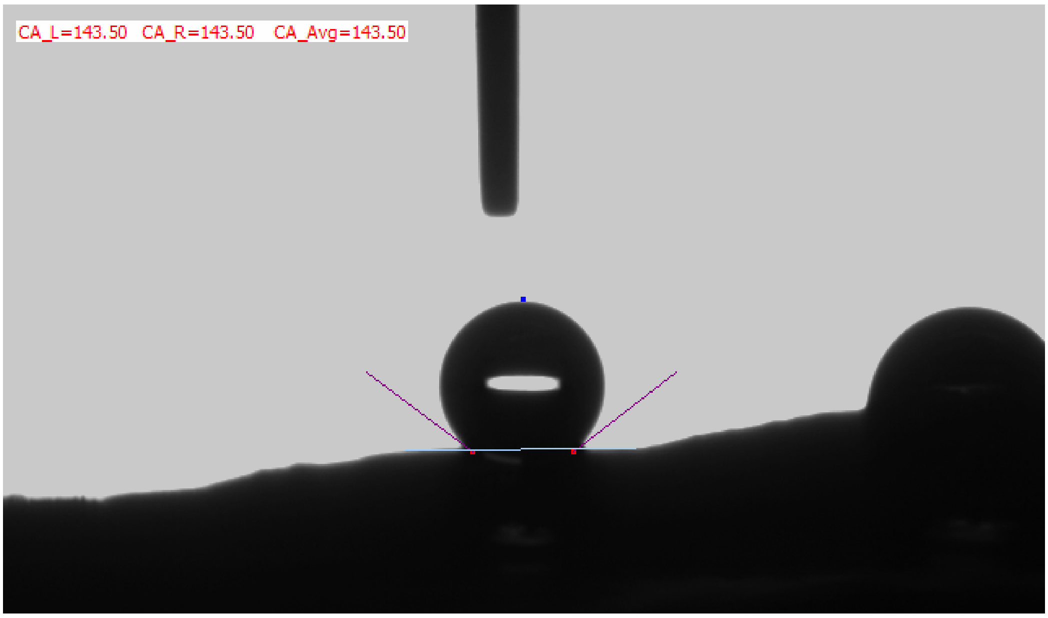
CA_L=143.50 CA_R=143.50 CA_Avg=143.50

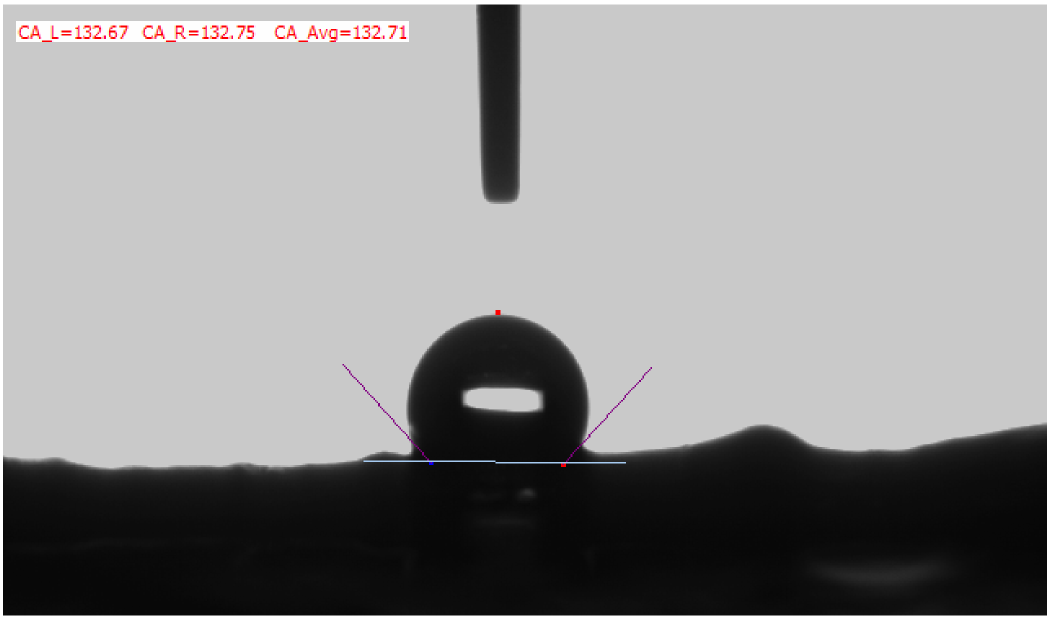
CA_L=132.67 CA_R=132.75 CA_Avg=132.71

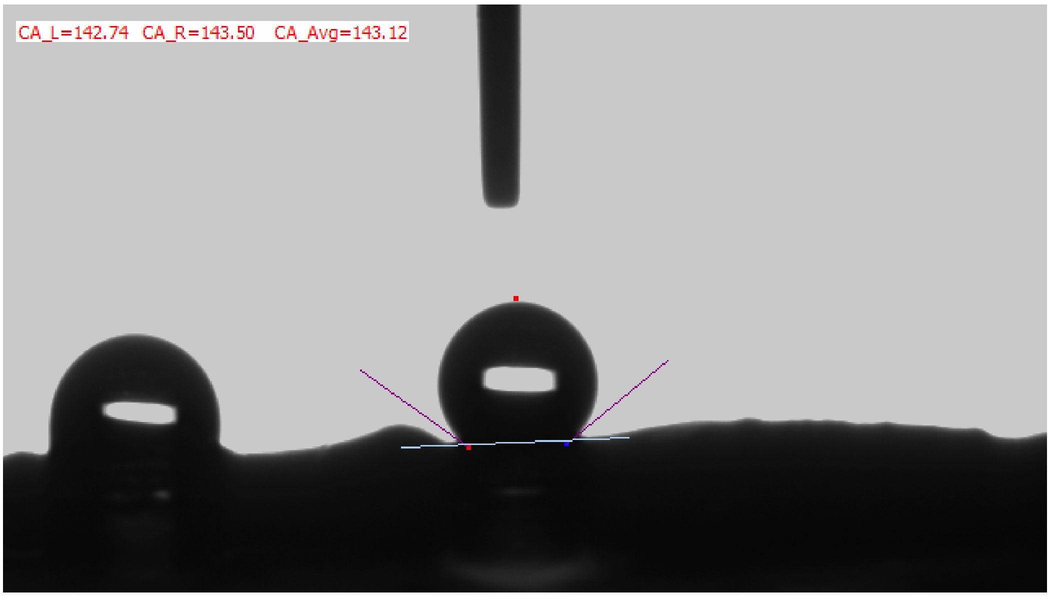
CA_L=142.74 CA_R=143.50 CA_Avg=143.12

N. hookeriana **(12 images)**

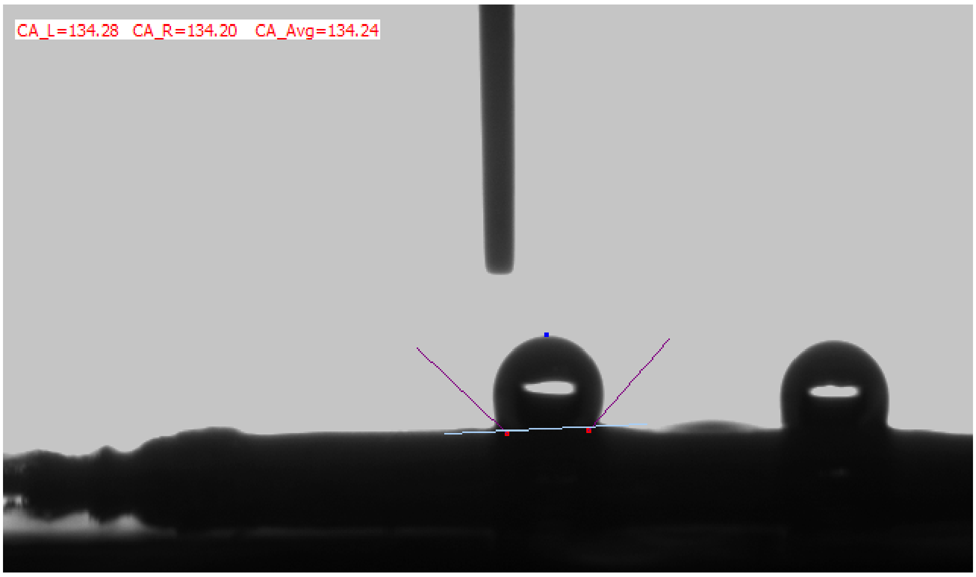

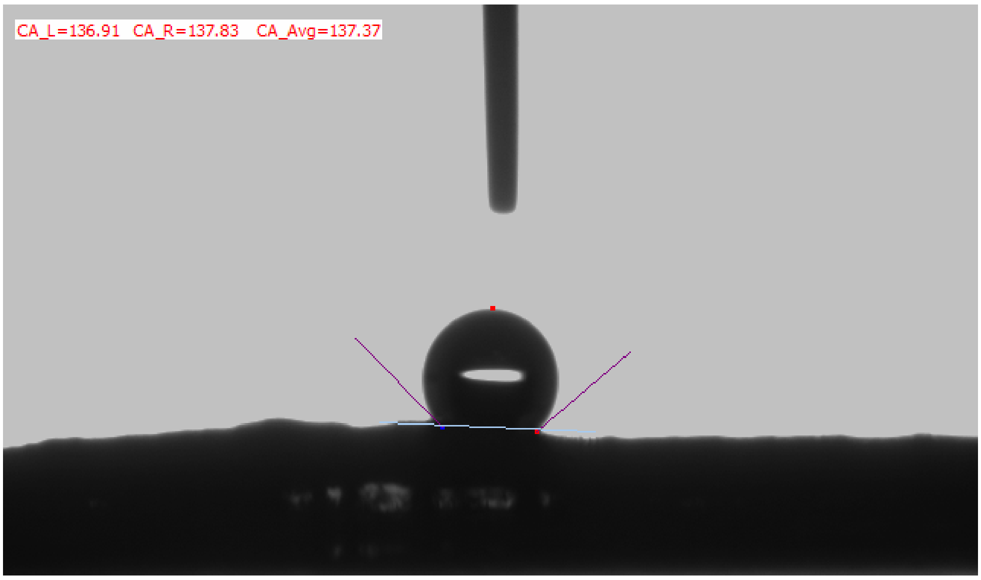

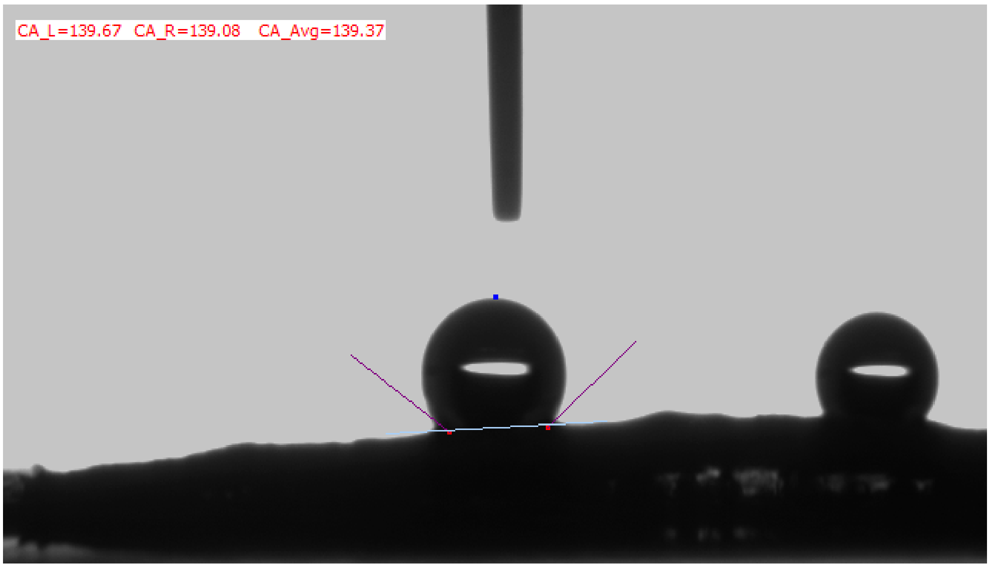

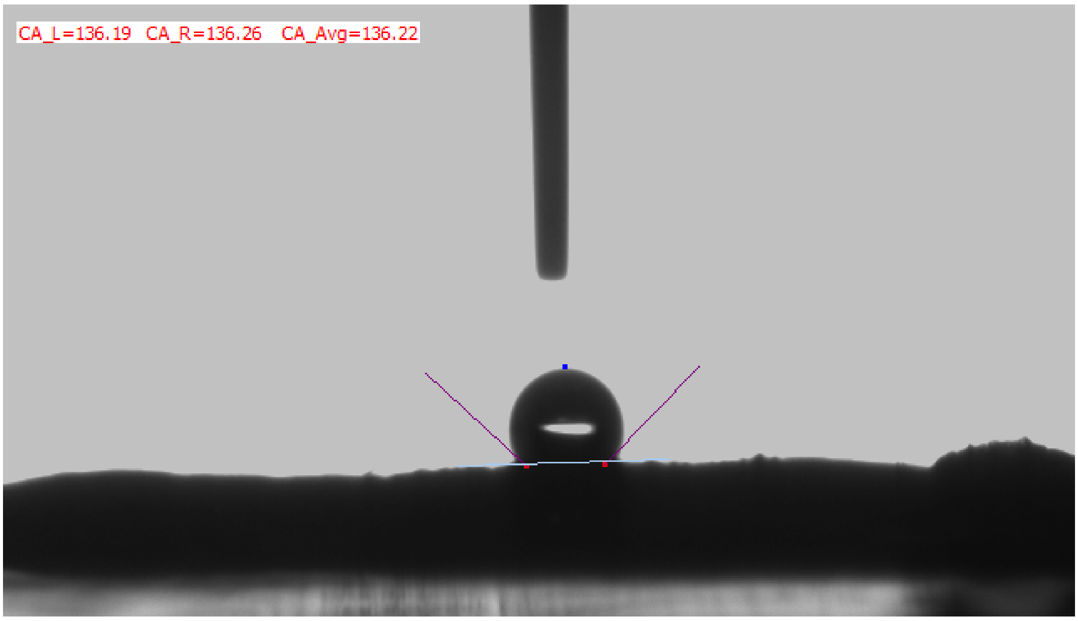
CA_L=136.19 CA_R=136.26 CA_Avg=136.22

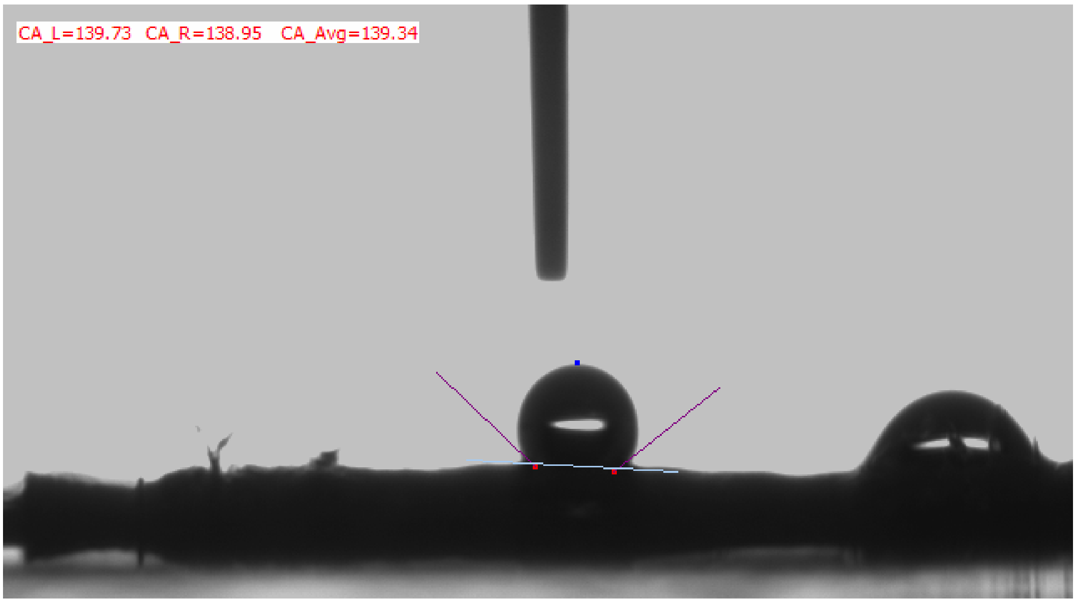
CA_L=139.73 CA_R=138.95 CA_Avg=139.34

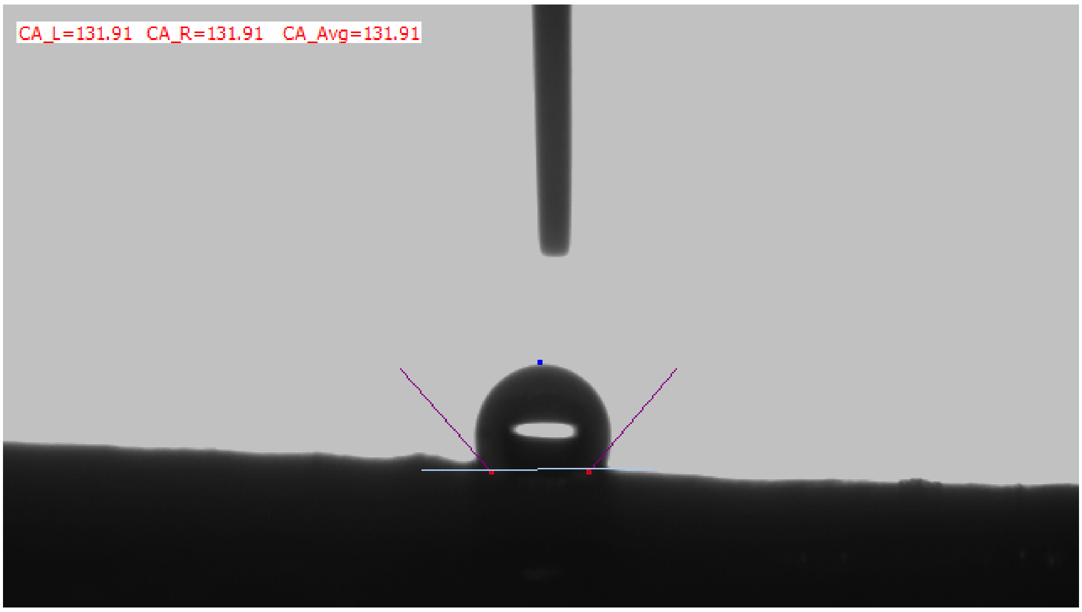
CA_L=131.91 CA_R=131.91 CA_Avg=131.91

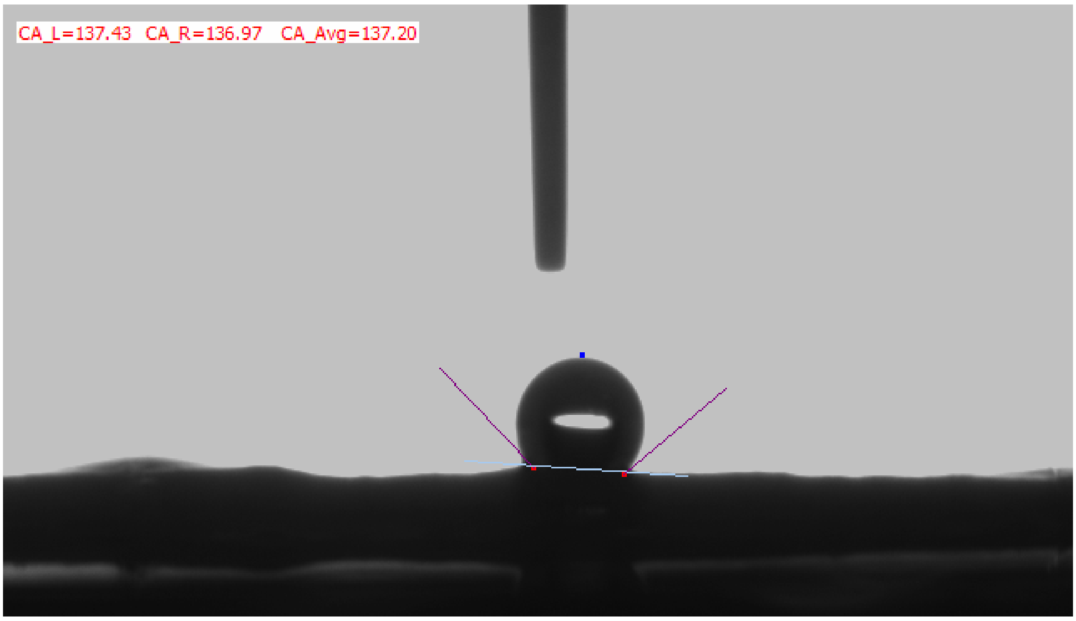
CA_L=137.43 CA_R=136.97 CA_Avg=137.20

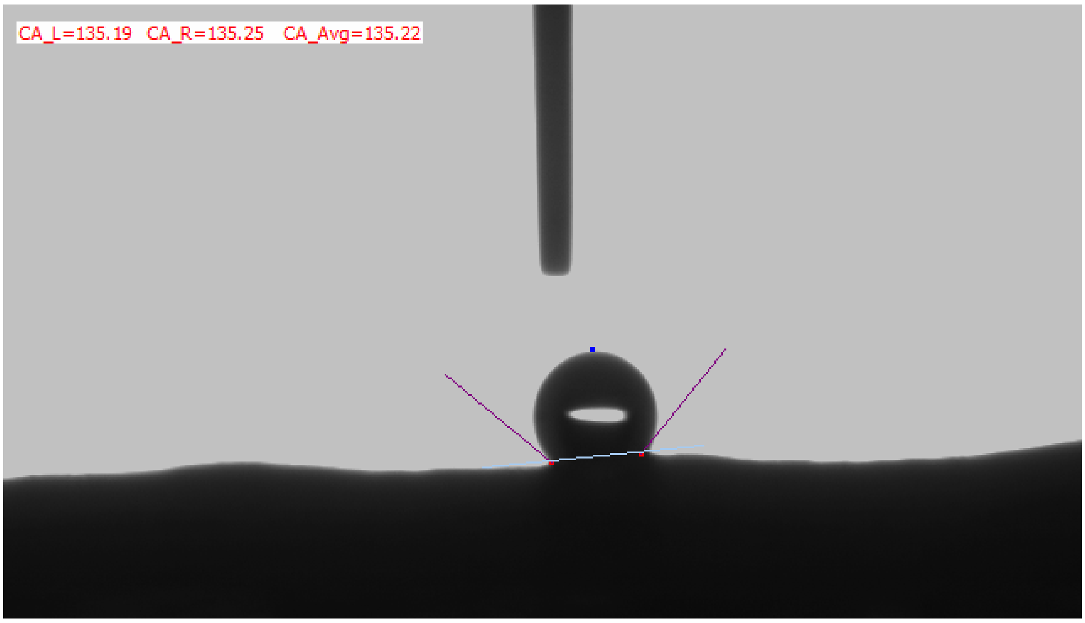
CA_L=135.19 CA_R=135.25 CA_Avg=135.22

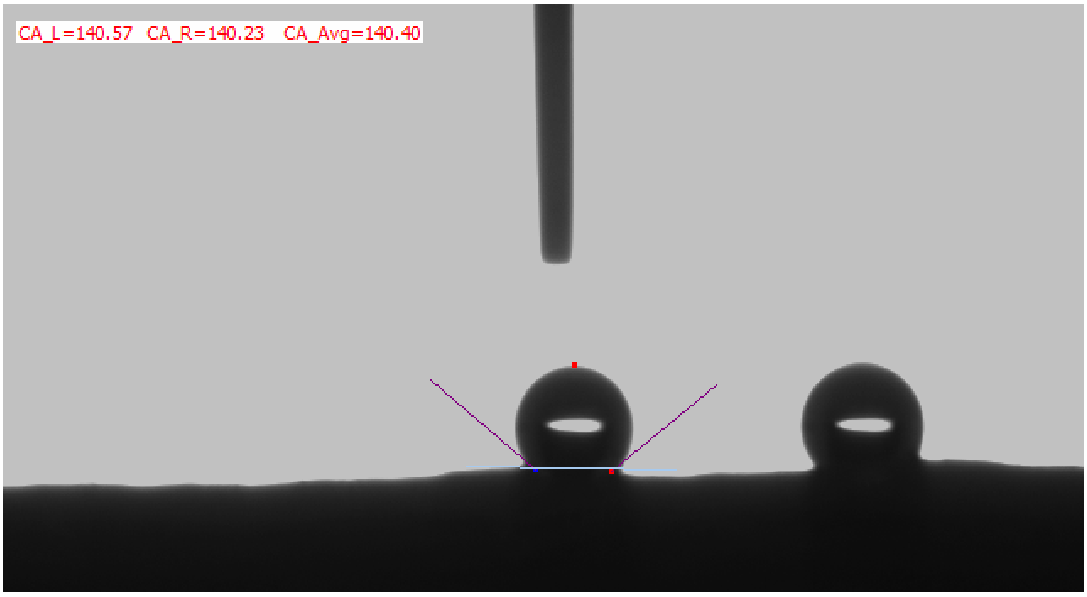
CA_L=140.57 CA_R=140.23 CA_Avg=140.40

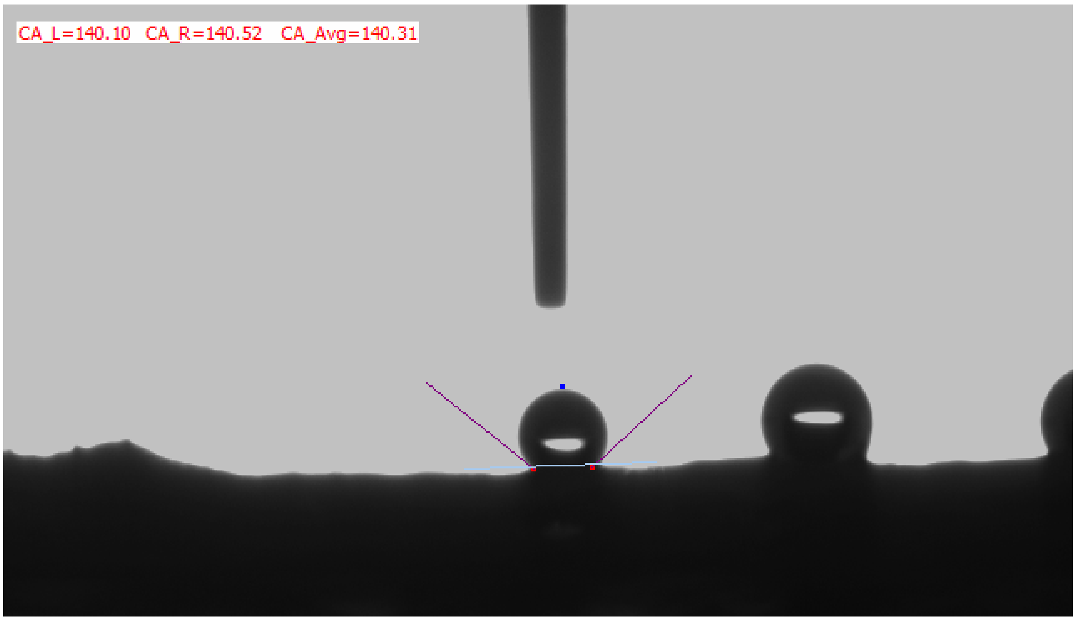
CA_L=140.10 CA_R=140.52 CA_Avg=140.31

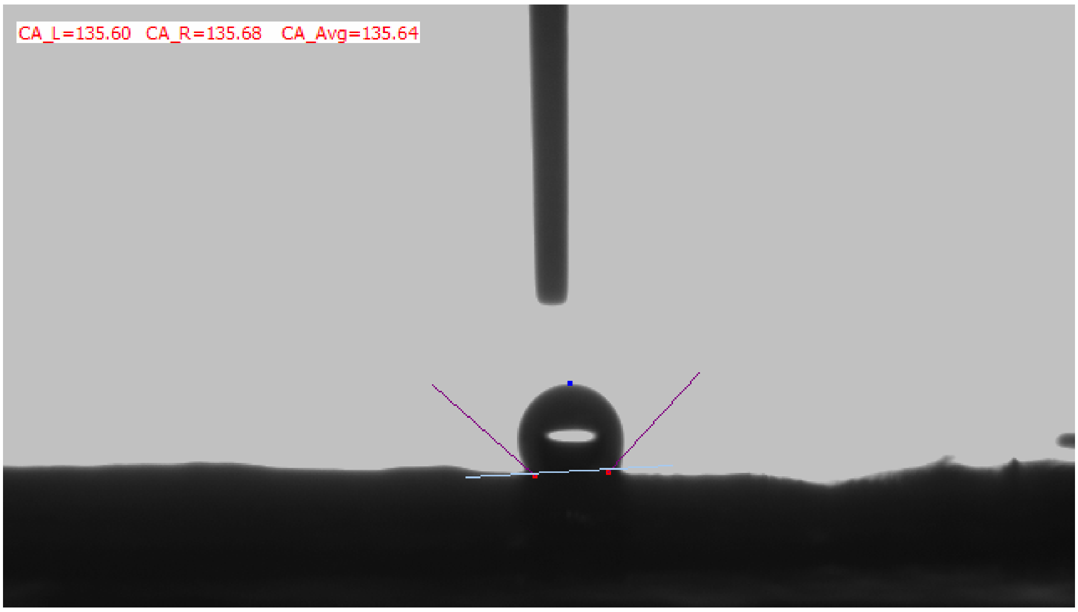
CA_L=135.60 CA_R=135.68 CA_Avg=135.64

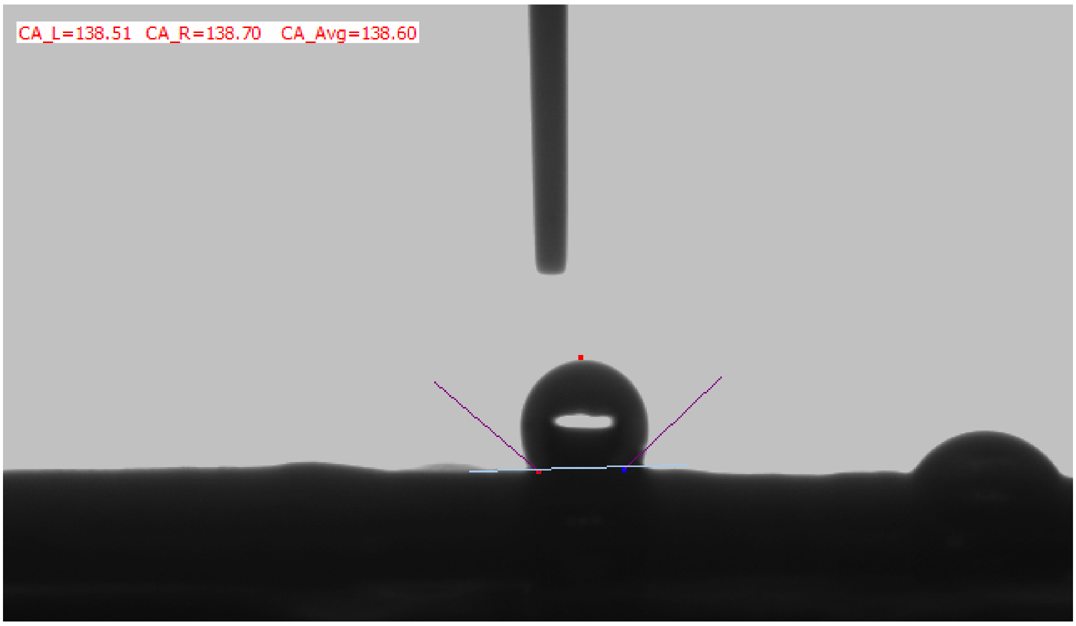
CA_L=138.51 CA_R=138.70 CA_Avg=138.60

N. khasiana **(16 images)**

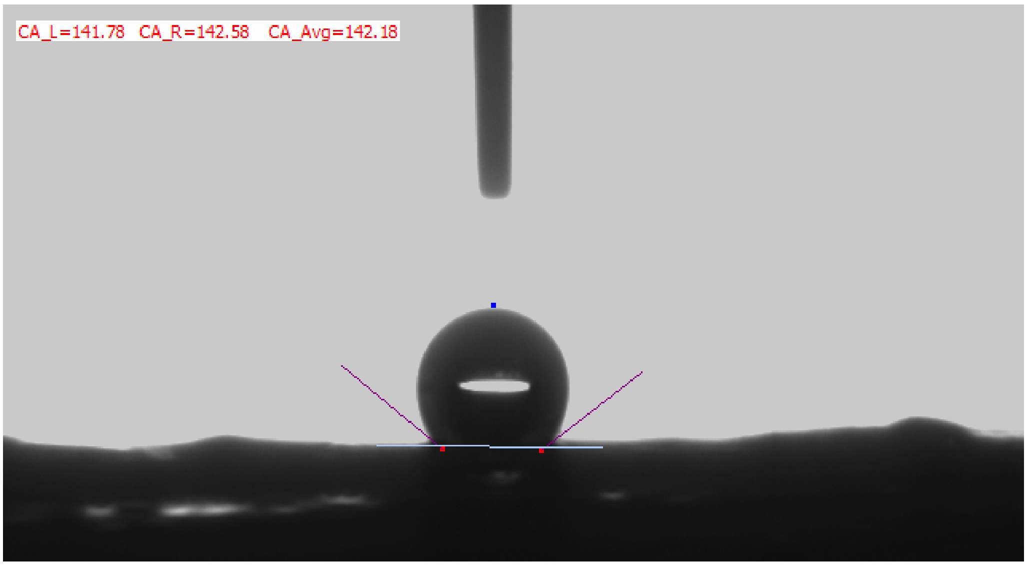

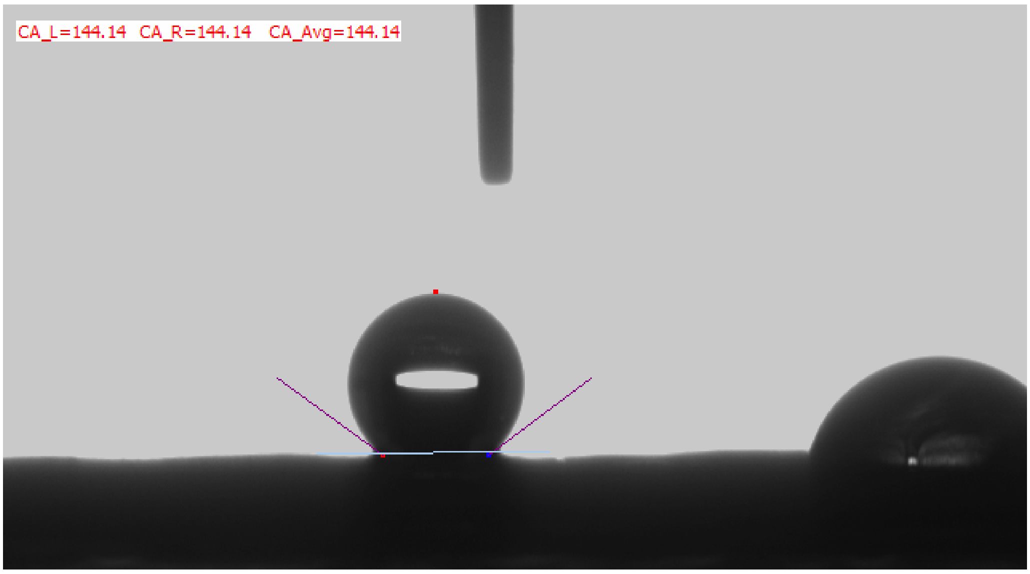

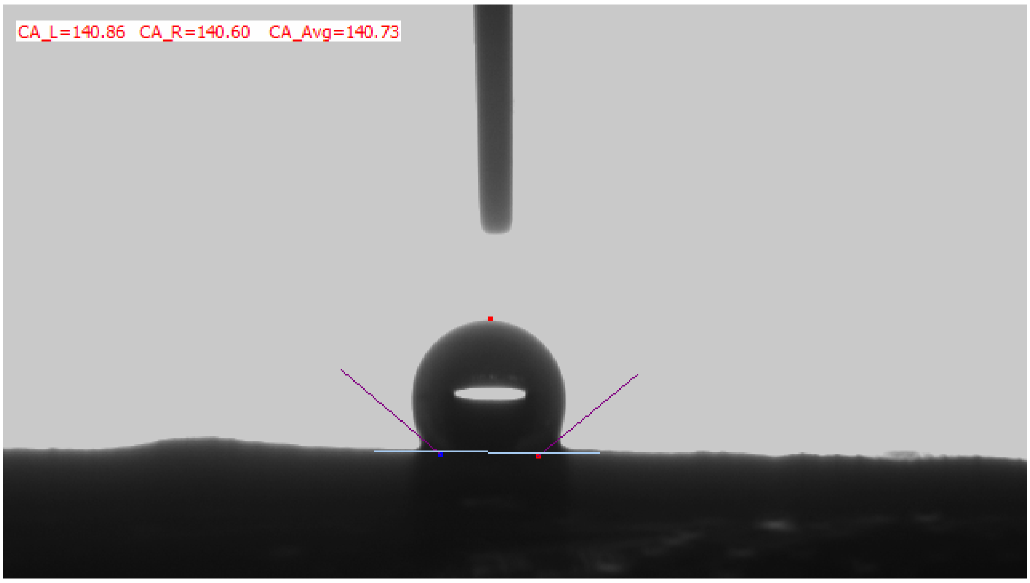

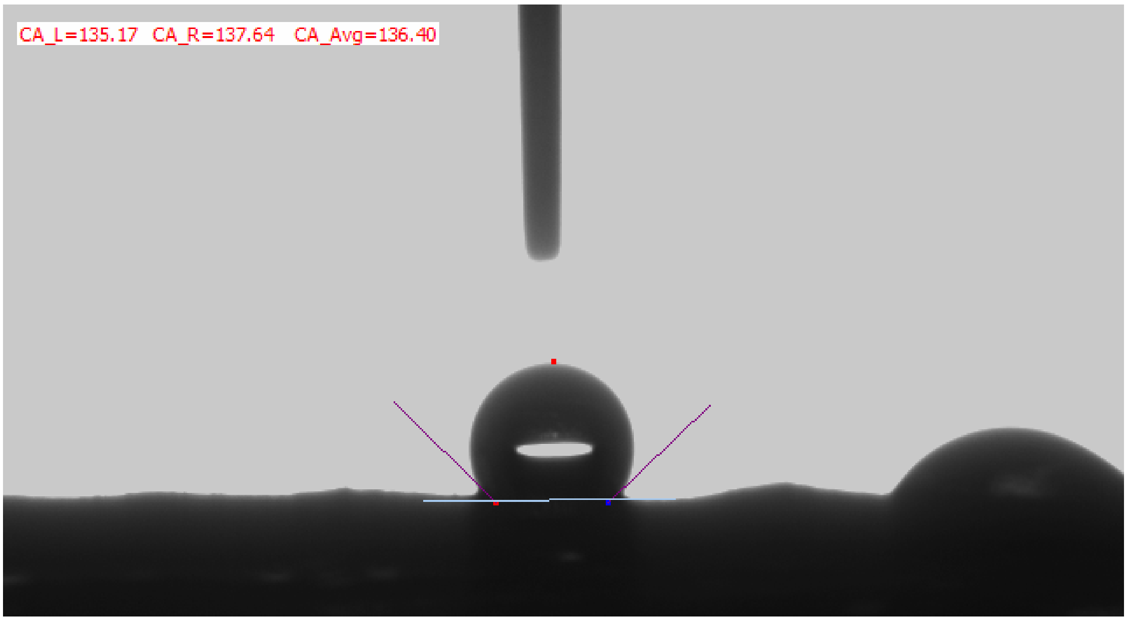
CA_L=135.17 CA_R=137.64 CA_Avg=136.40

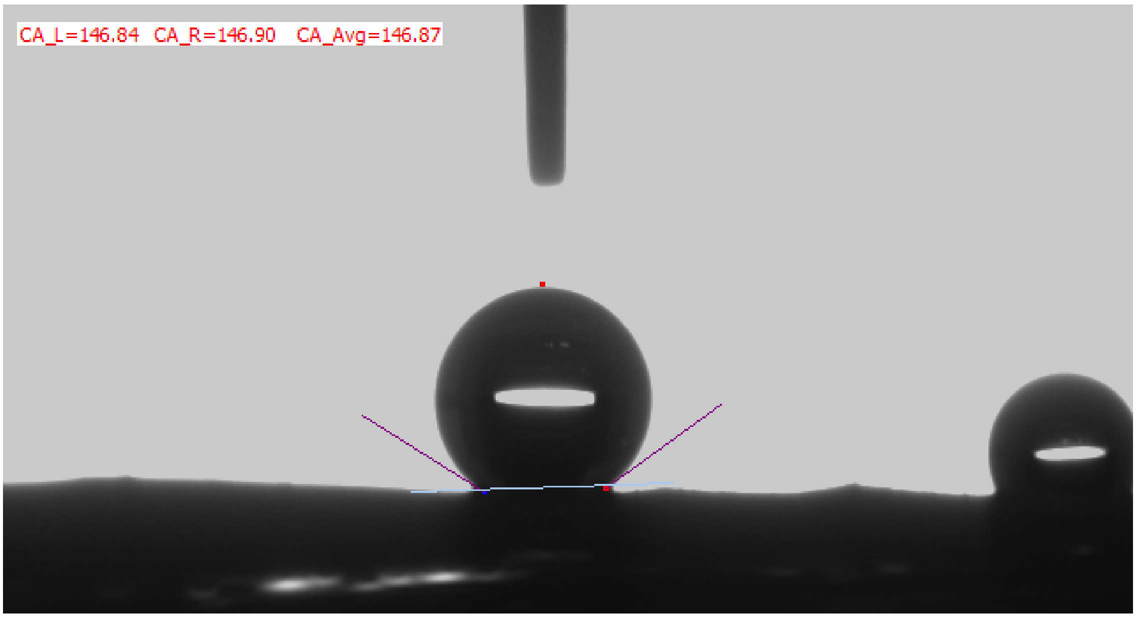
CA_L=146.84 CA_R=146.90 CA_Avg=146.87

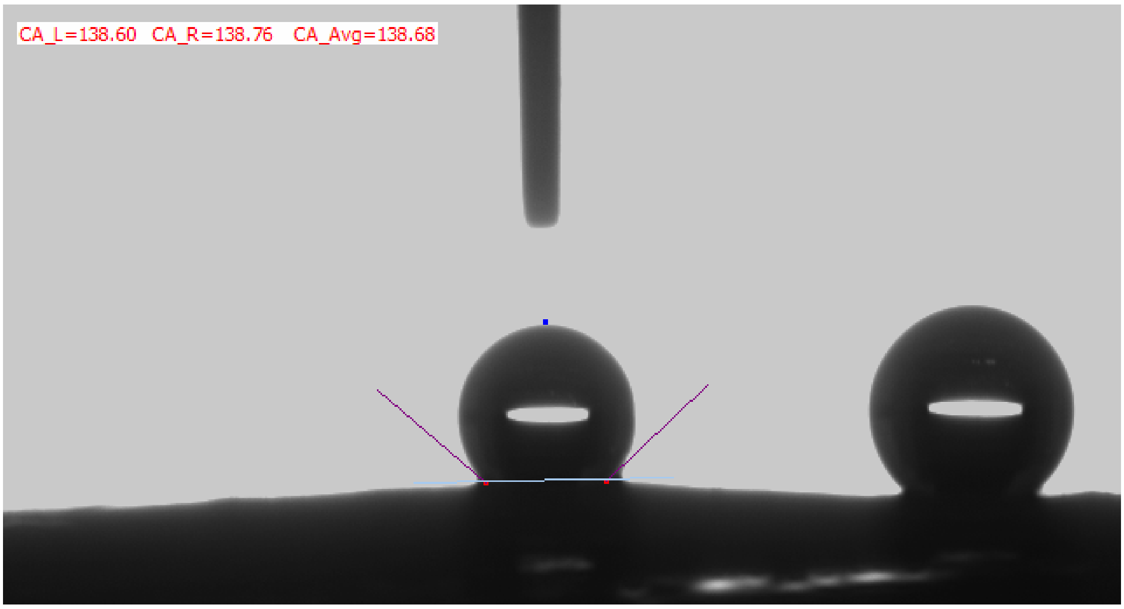
CA_L=138.60 CA_R=138.76 CA_Avg=138.68

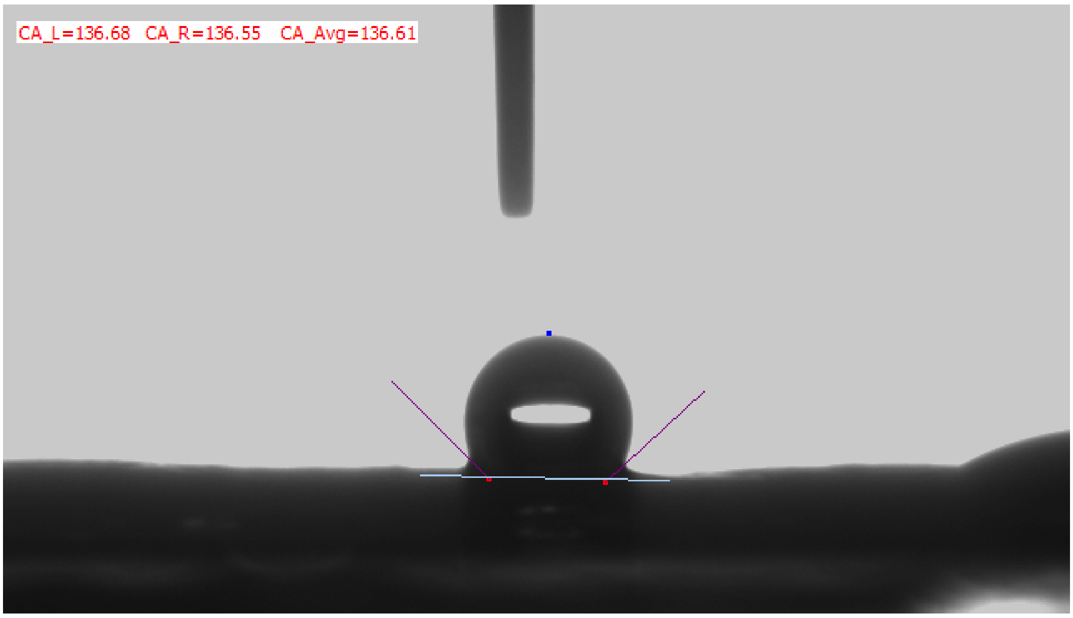
CA_L=136.68 CA_R=136.55 CA_Avg=136.61

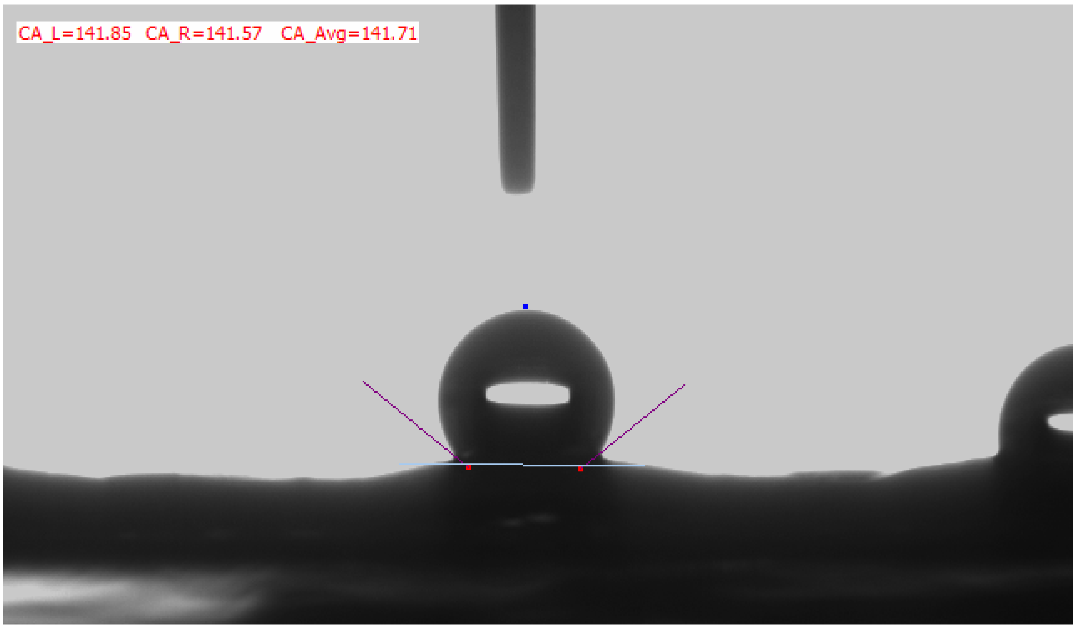
CA_L=141.85 CA_R=141.57 CA_Avg=141.71

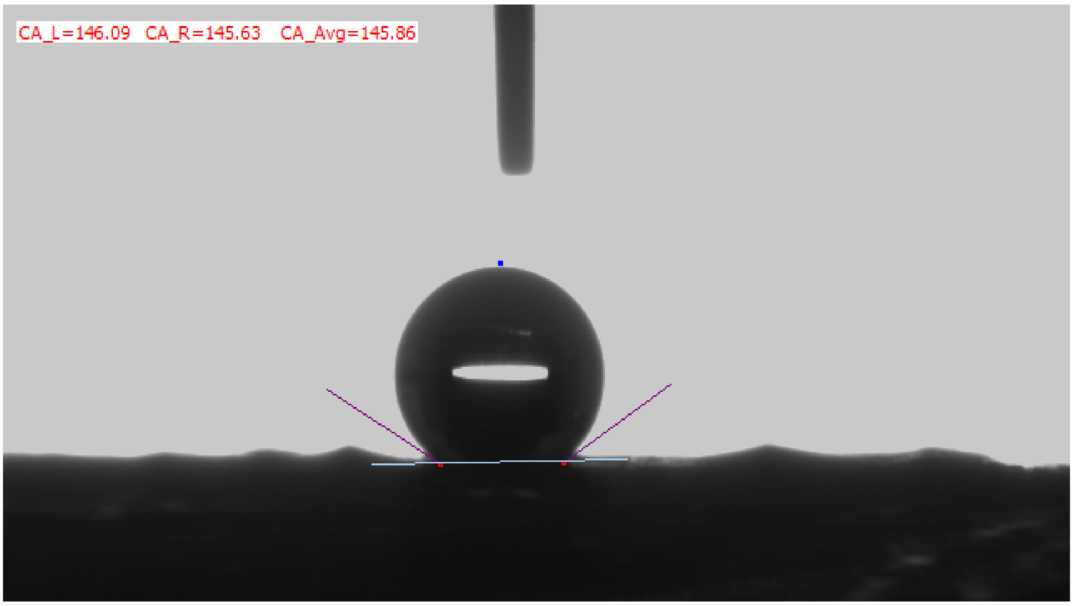
CA_L=146.09 CA_R=145.63 CA_Avg=145.86

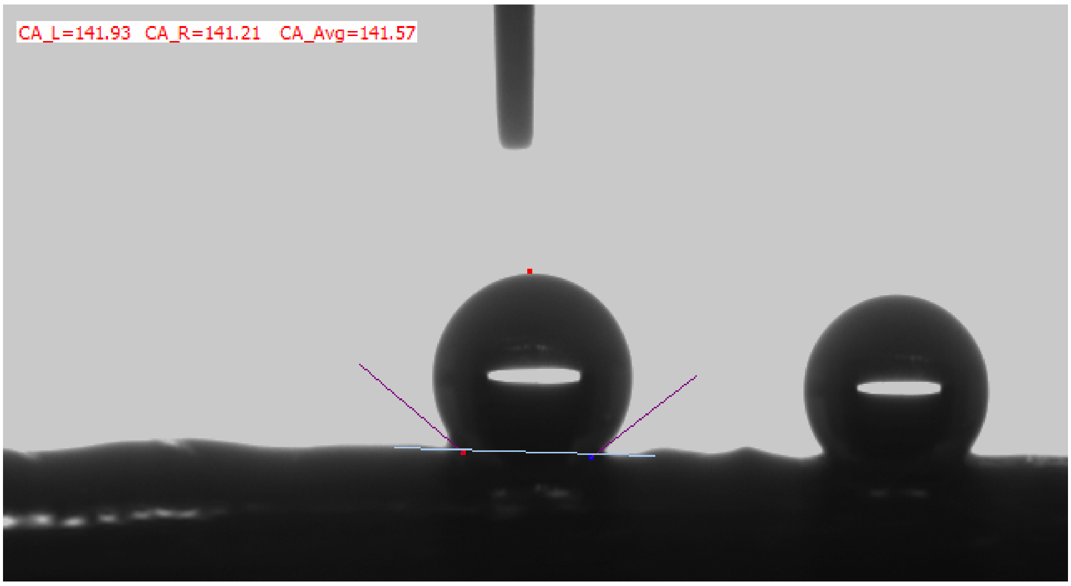
CA_L=141.93 CA_R=141.21 CA_Avg=141.57

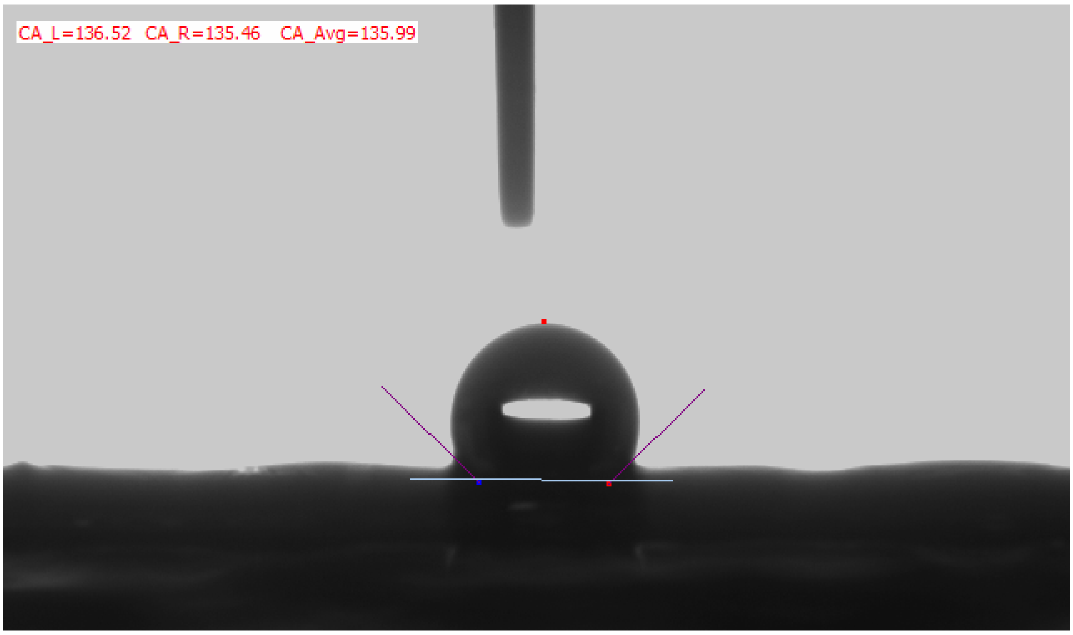
CA_L=136.52 CA_R=135.46 CA_Avg=135.99

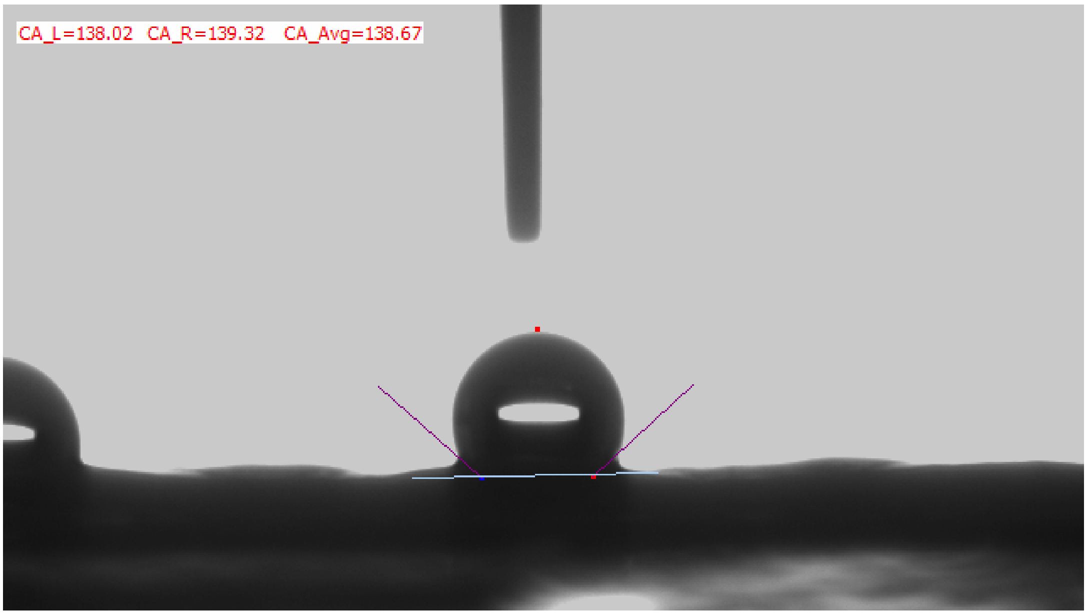
CA_L=138.02 CA_R=139.32 CA_Avg=138.67

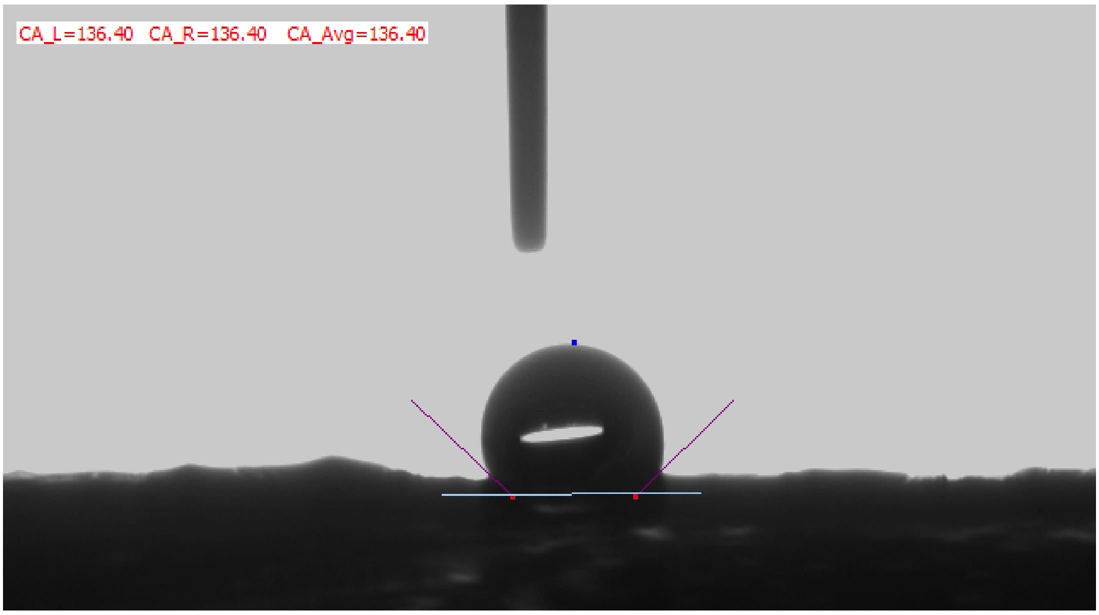
CA_L=136.40 CA_R=136.40 CA_Avg=136.40

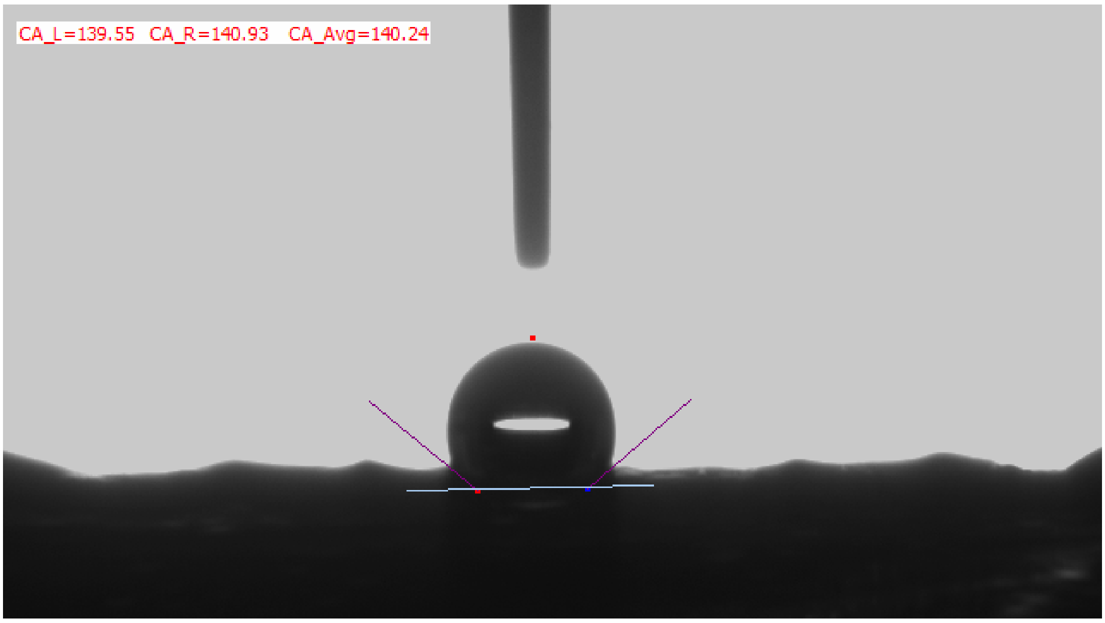
CA_L=139.55 CA_R=140.93 CA_Avg=140.24

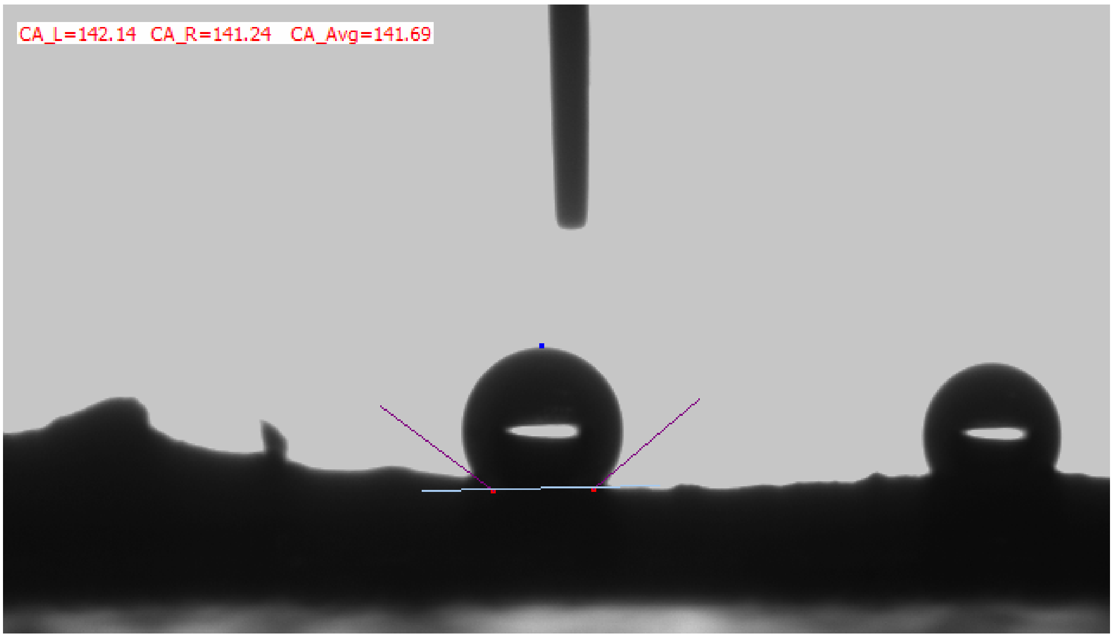
CA_L=142.14 CA_R=141.24 CA_Avg=141.69

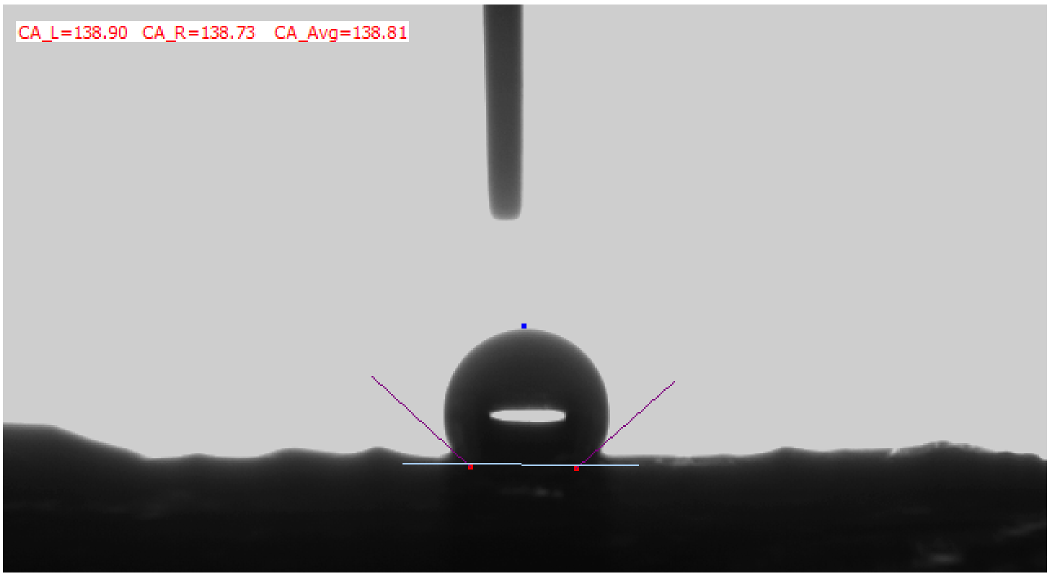

N. mirabilis **(16 images)**

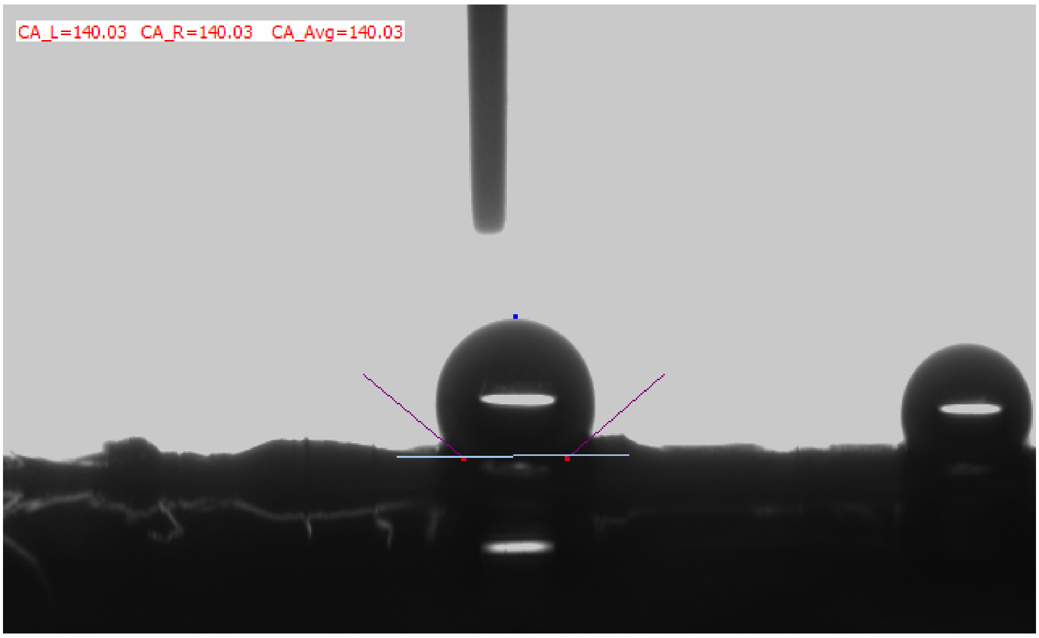

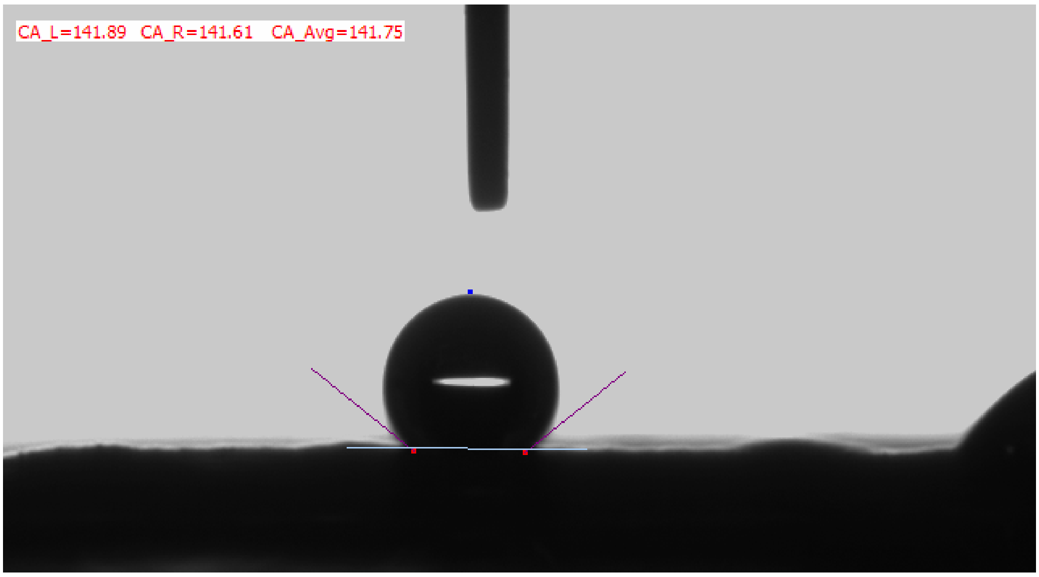

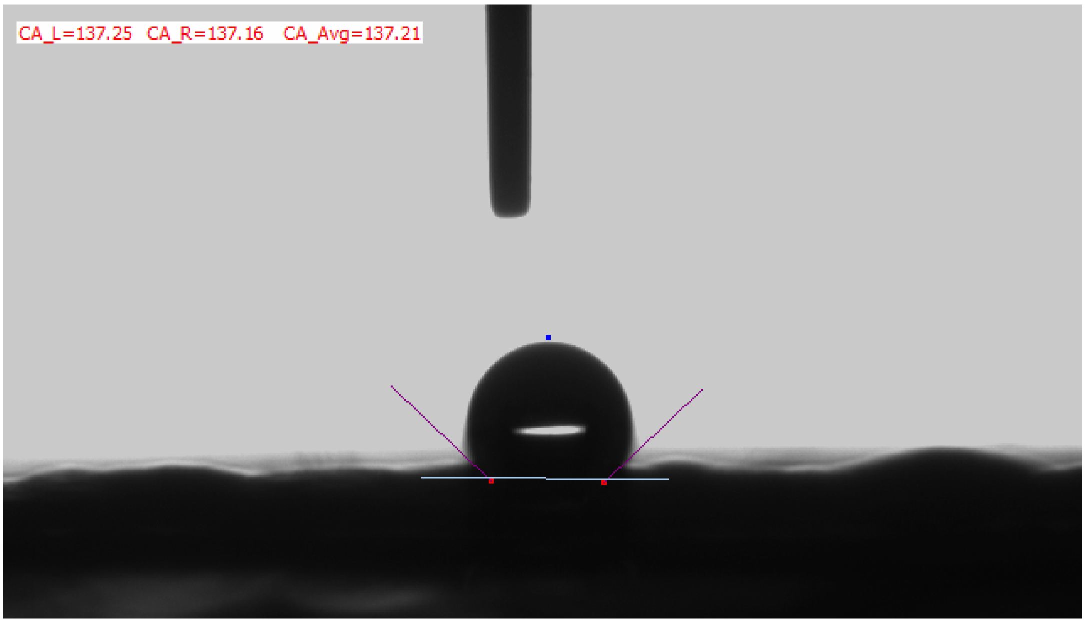
CA_L=137.25 CA_R=137.16 CA_Avg=137.21

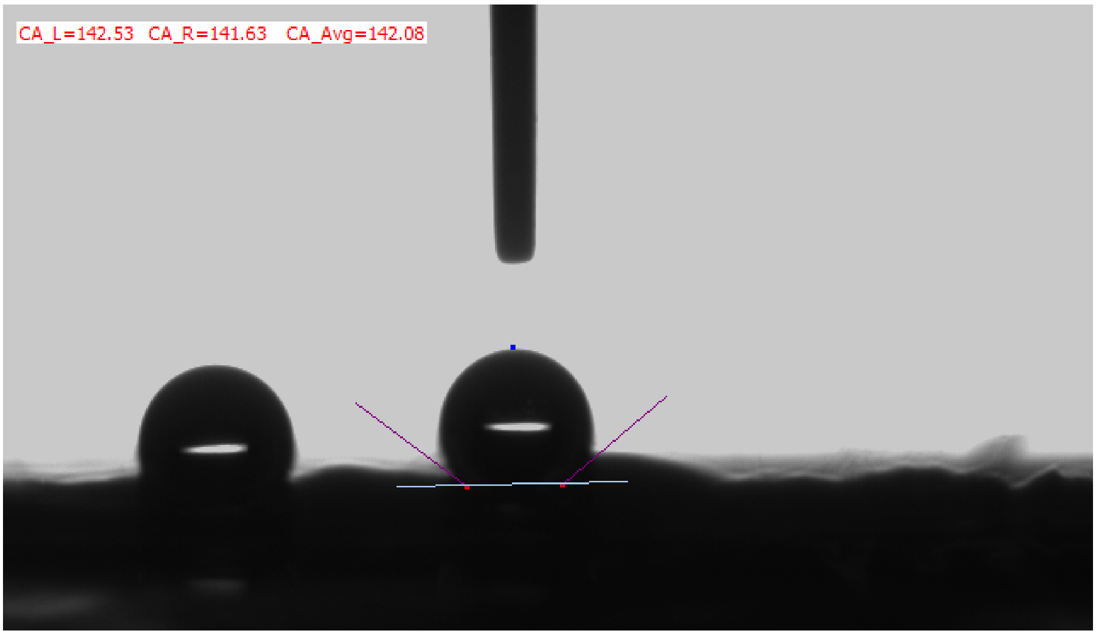
CA_L=142.53 CA_R=141.63 CA_Avg=142.08

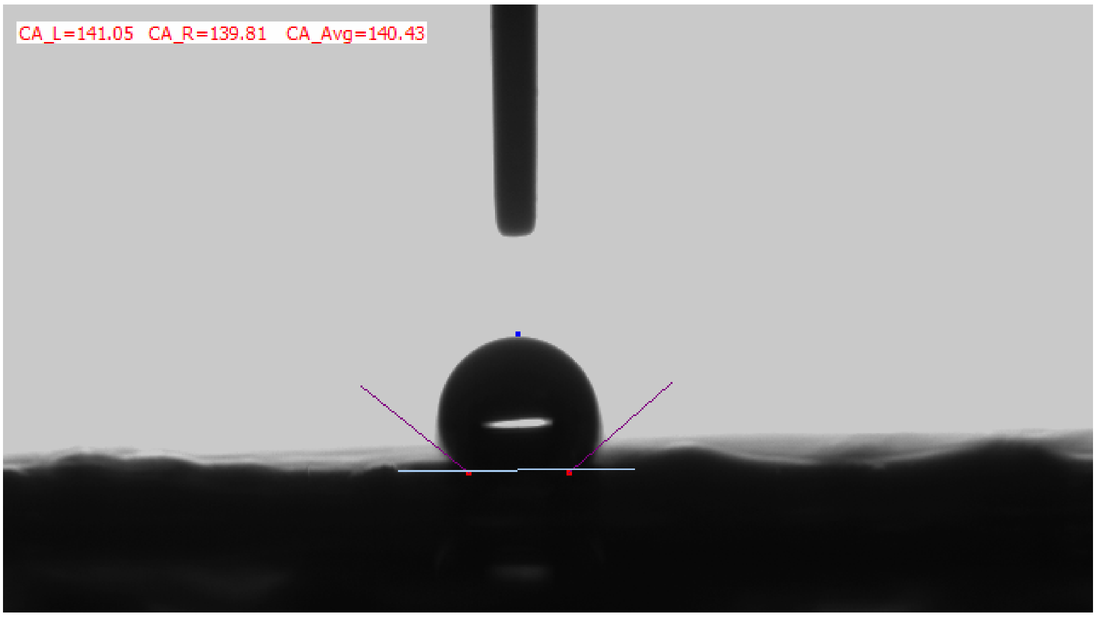
CA_L=141.05 CA_R=139.81 CA_Avg=140.43

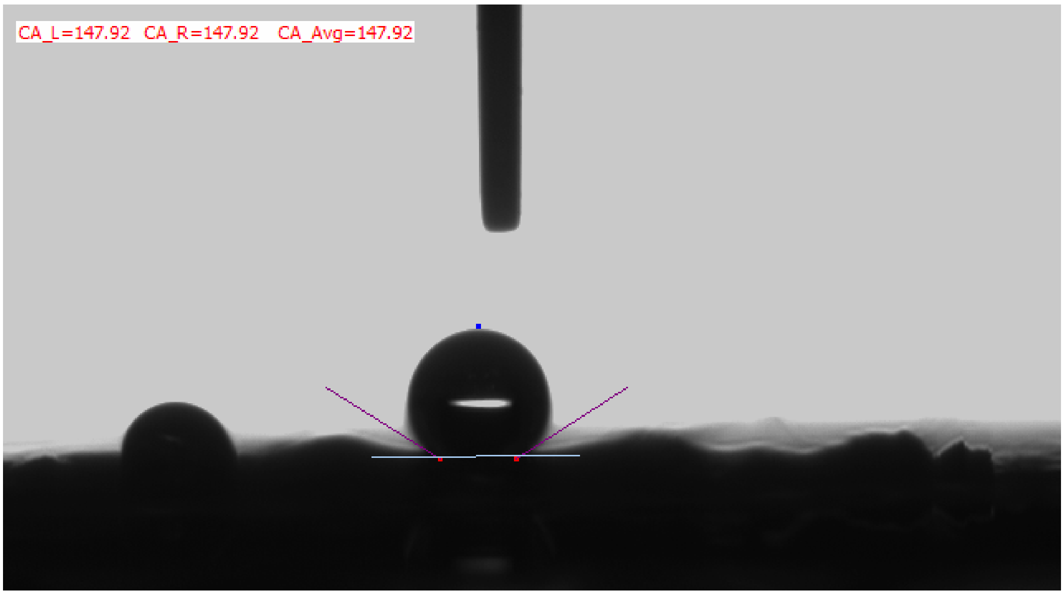
CA_L=147.92 CA_R=147.92 CA_Avg=147.92

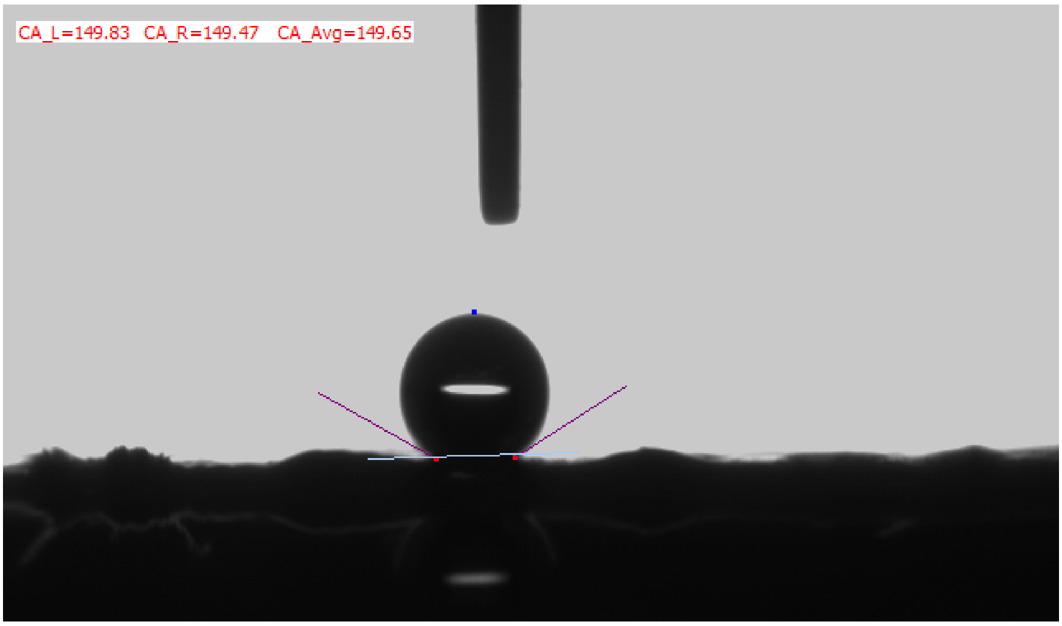
CA_L=149.83 CA_R=149.47 CA_Avg=149.65

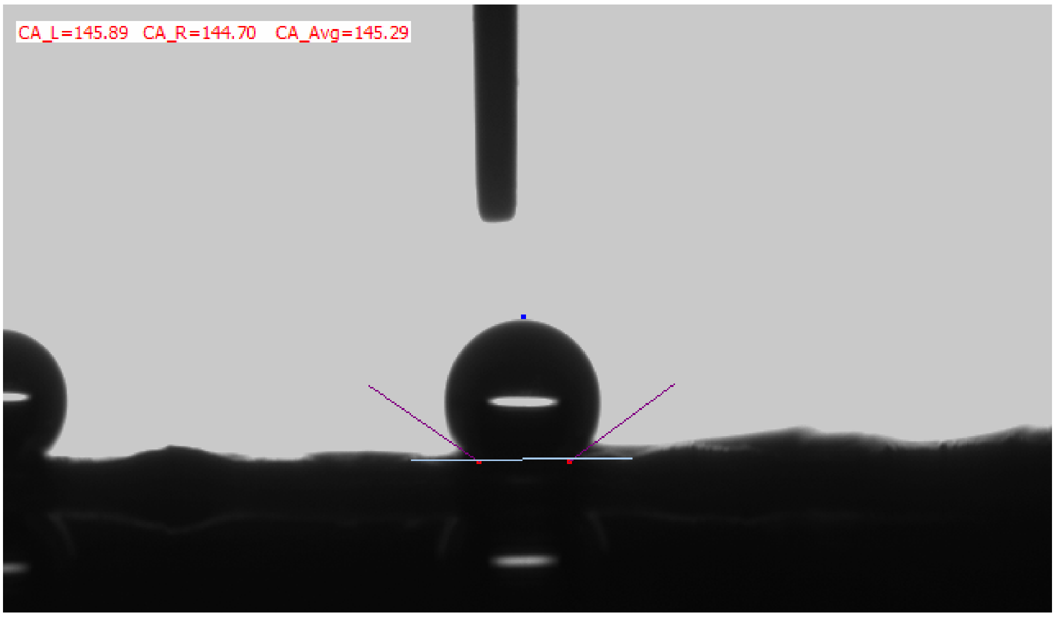
CA_L=145.89 CA_R=144.70 CA_Avg=145.29

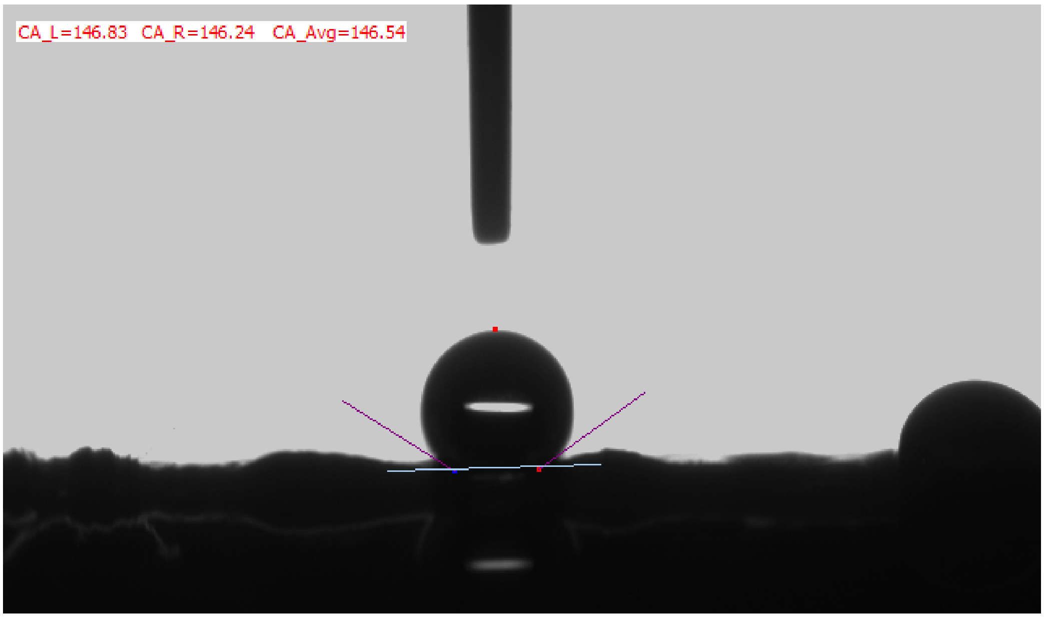
CA_L=146.83 CA_R=146.24 CA_Avg=146.54

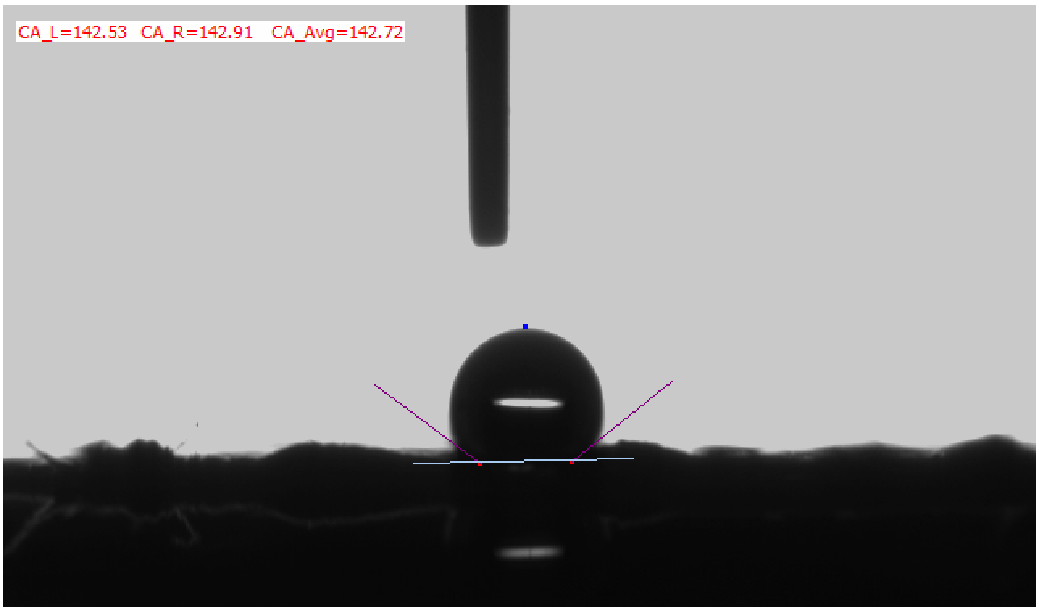
CA_L=142.53 CA_R=142.91 CA_Avg=142.72

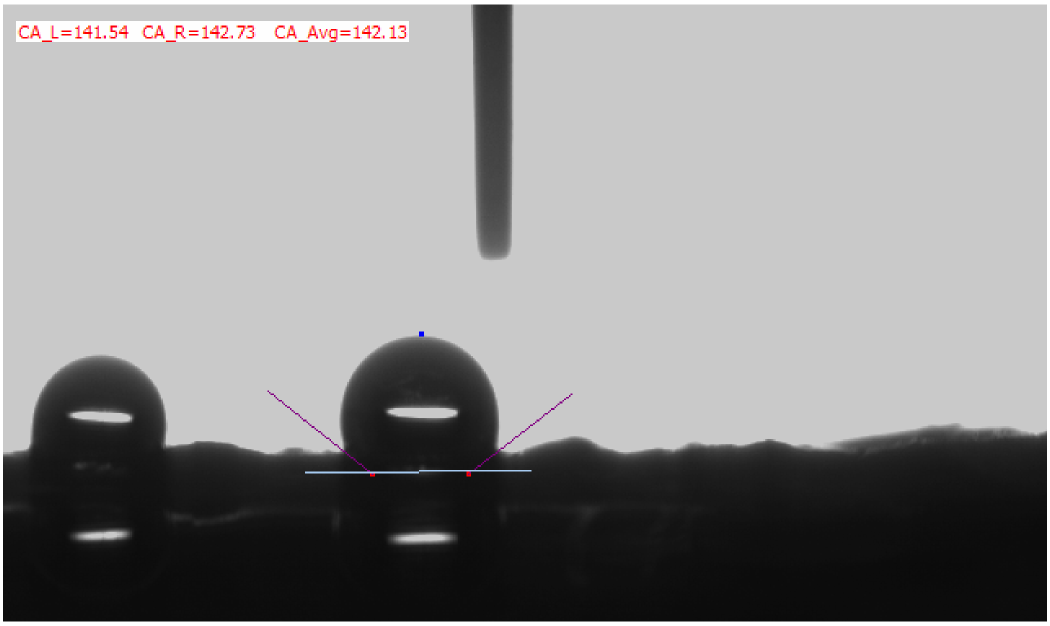
CA_L=141.54 CA_R=142.73 CA_Avg=142.13

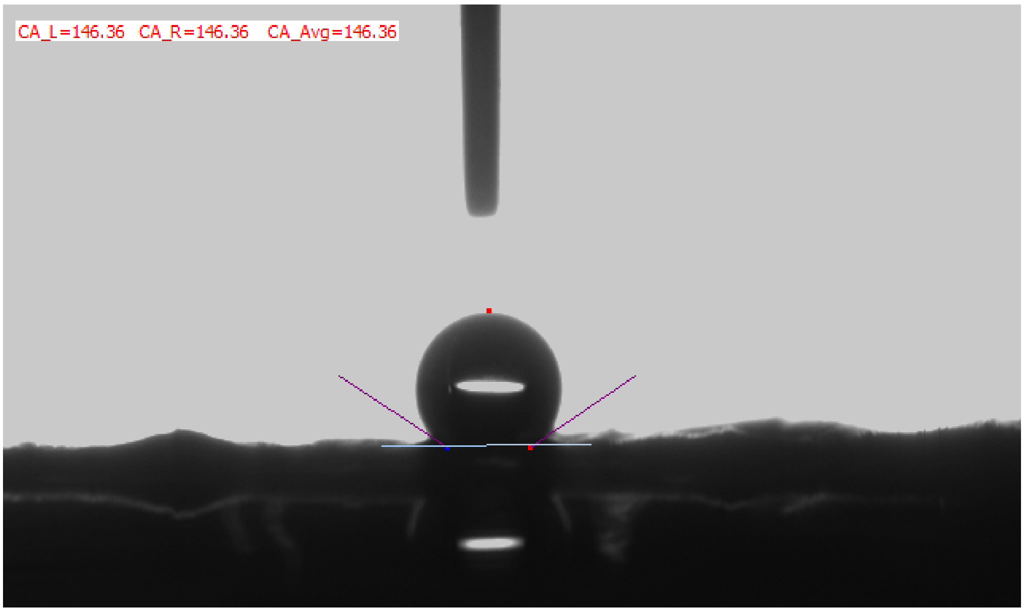
CA_L=146.36 CA_R=146.36 CA_Avg=146.36

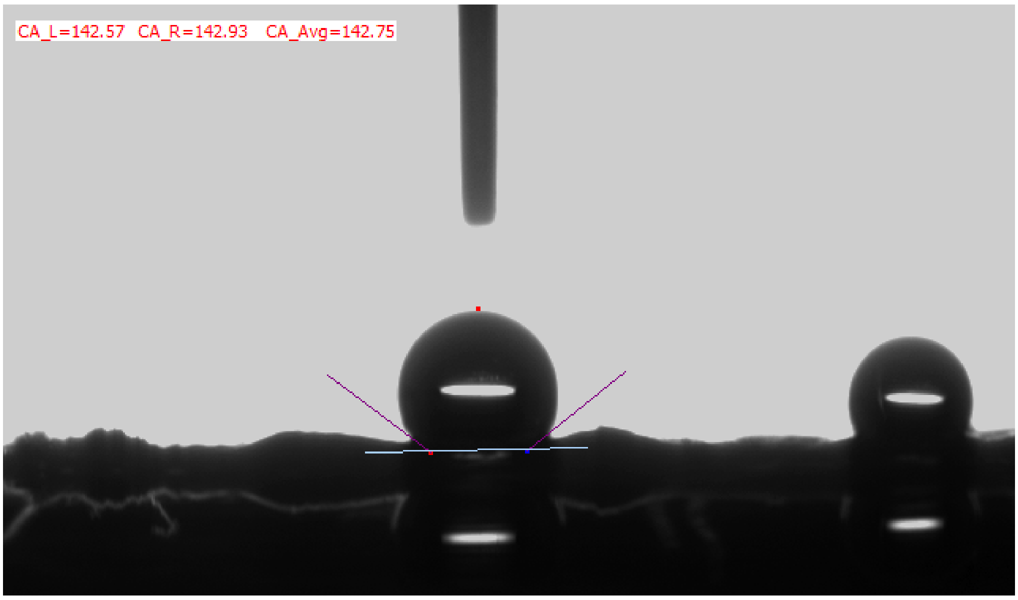
CA_L=142.57 CA_R=142.93 CA_Avg=142.75

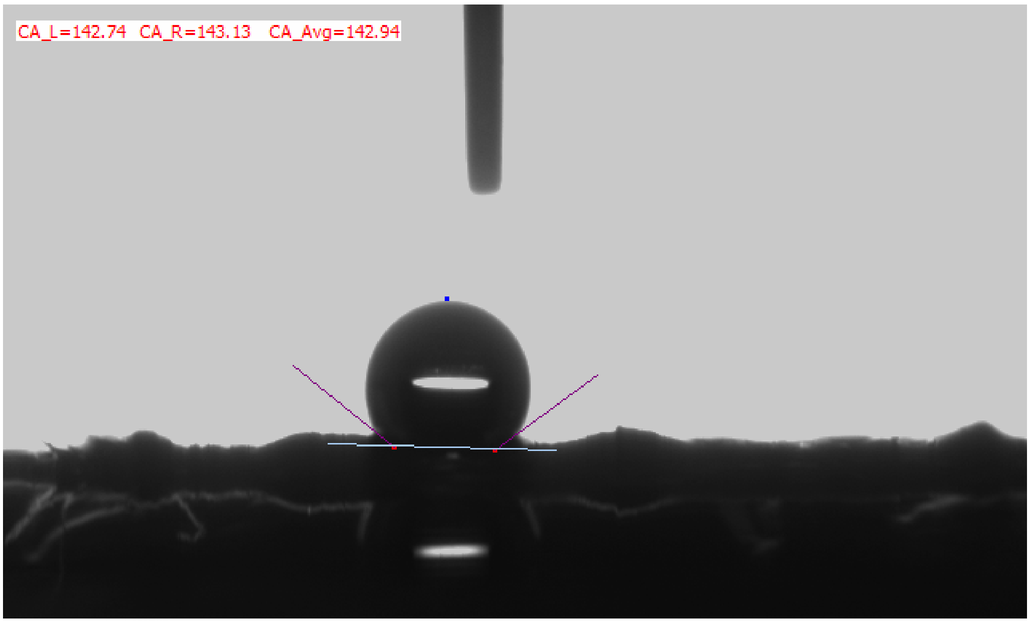
CA_L=142.74 CA_R=143.13 CA_Avg=142.94

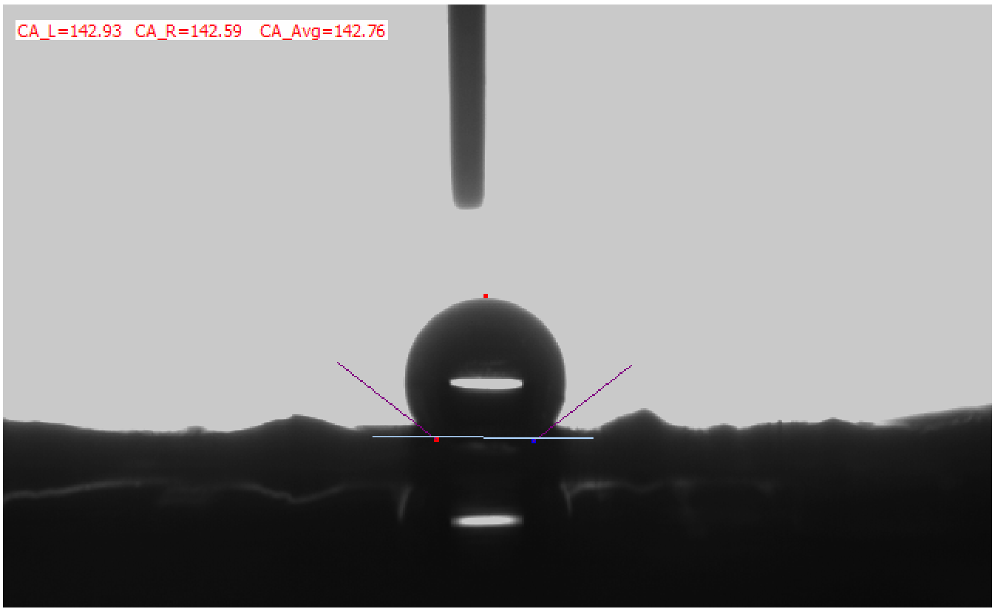

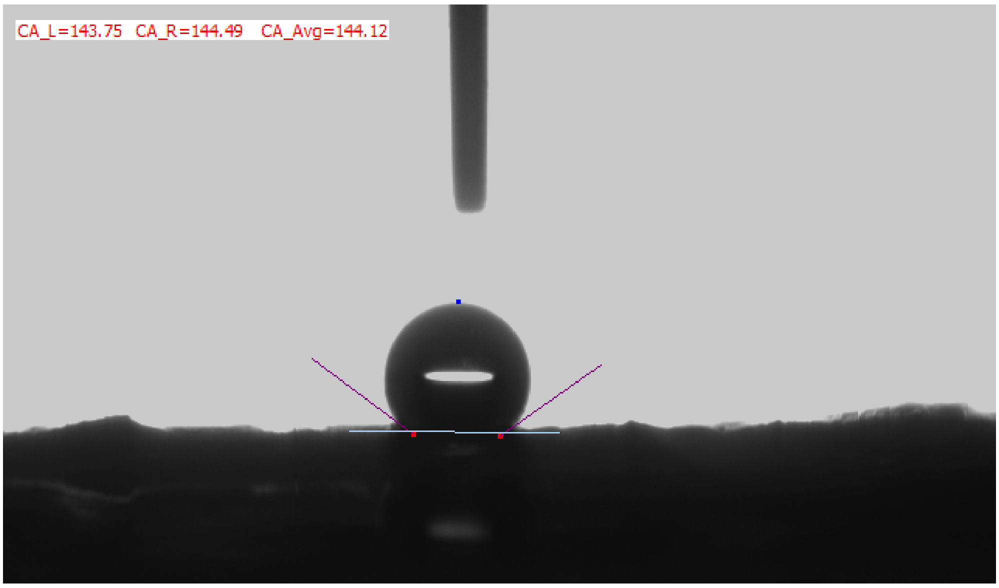

***N. Miranda* (16 images)**

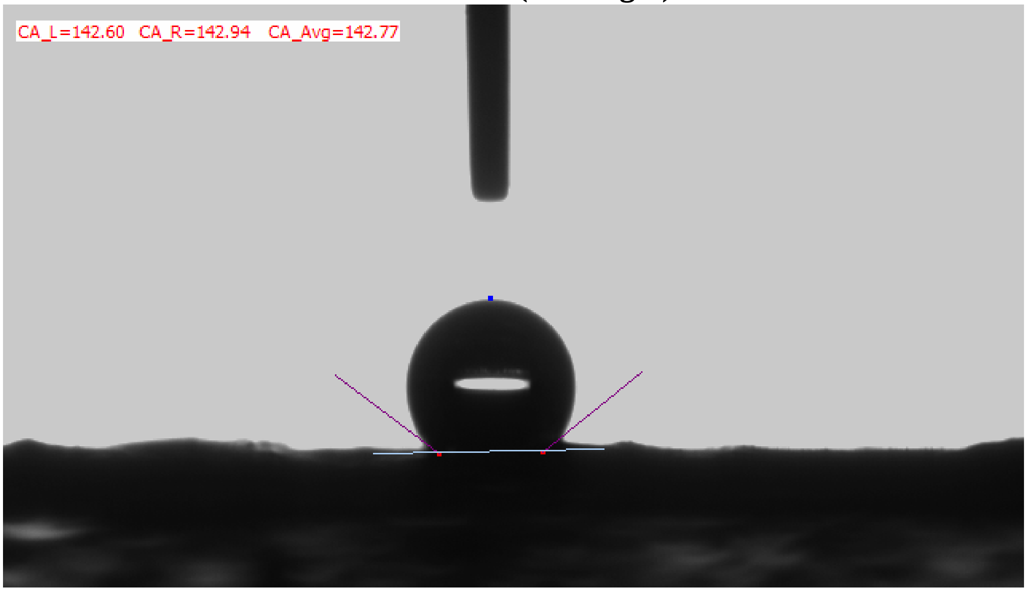

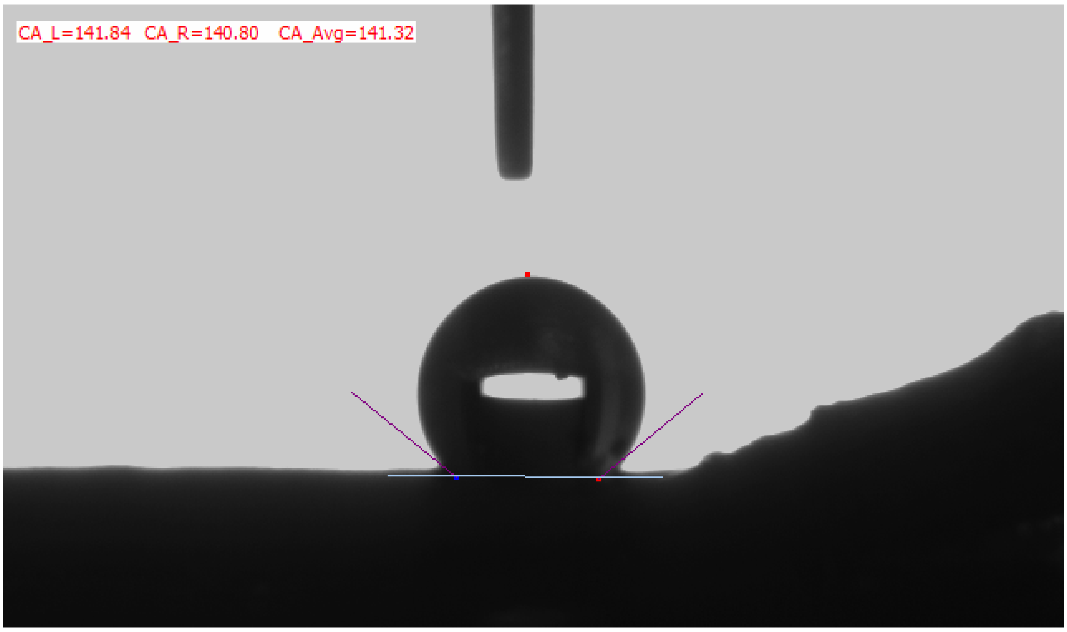
CA_L=141.84 CA_R=140.80 CA_Avg=141.32

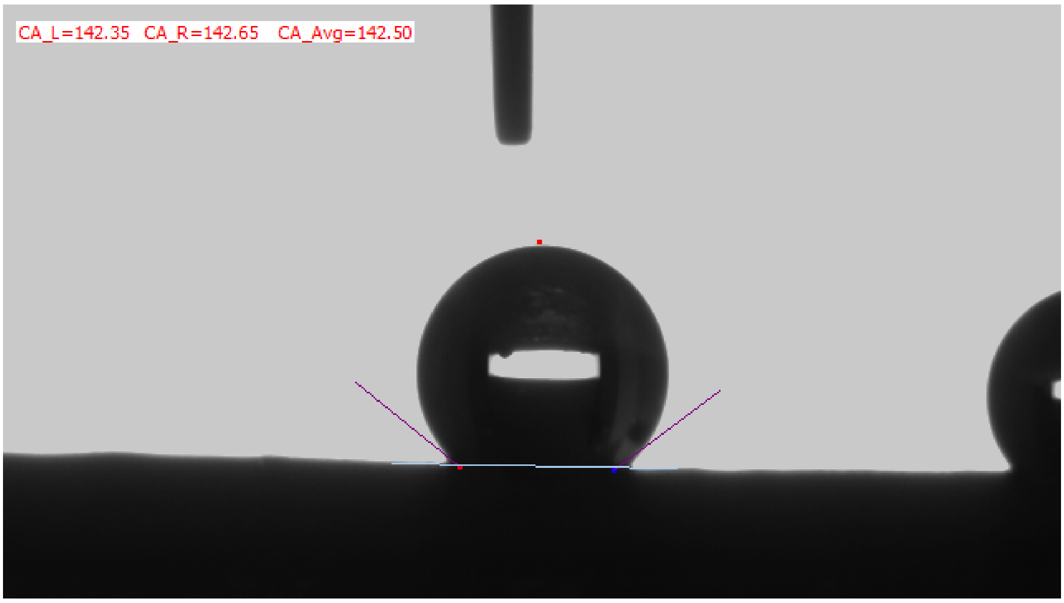
CA_L=142.35 CA_R=142.65 CA_Avg=142.50

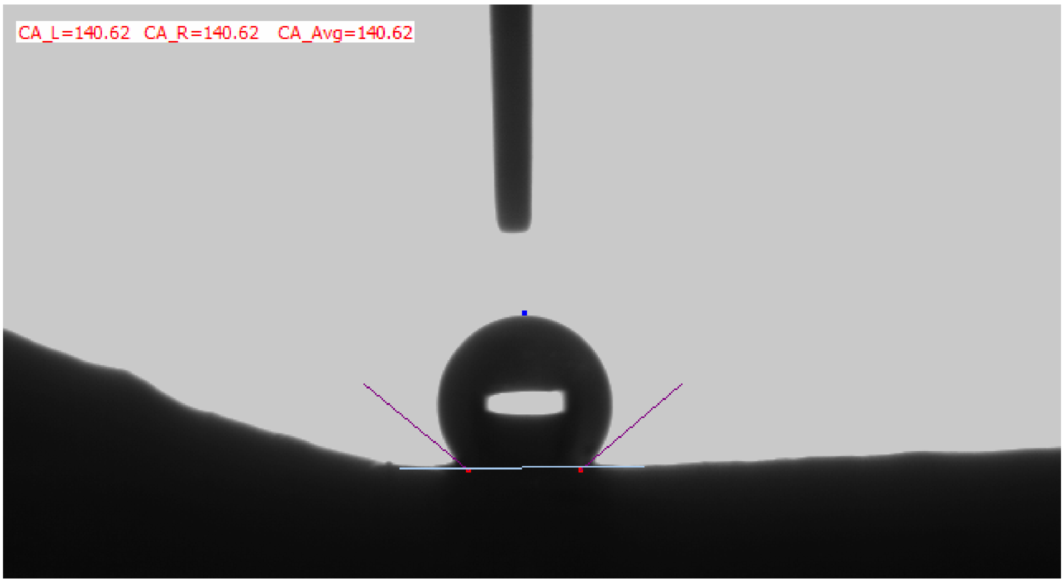
CA_L=140.62 CA_R=140.62 CA_Avg=140.62

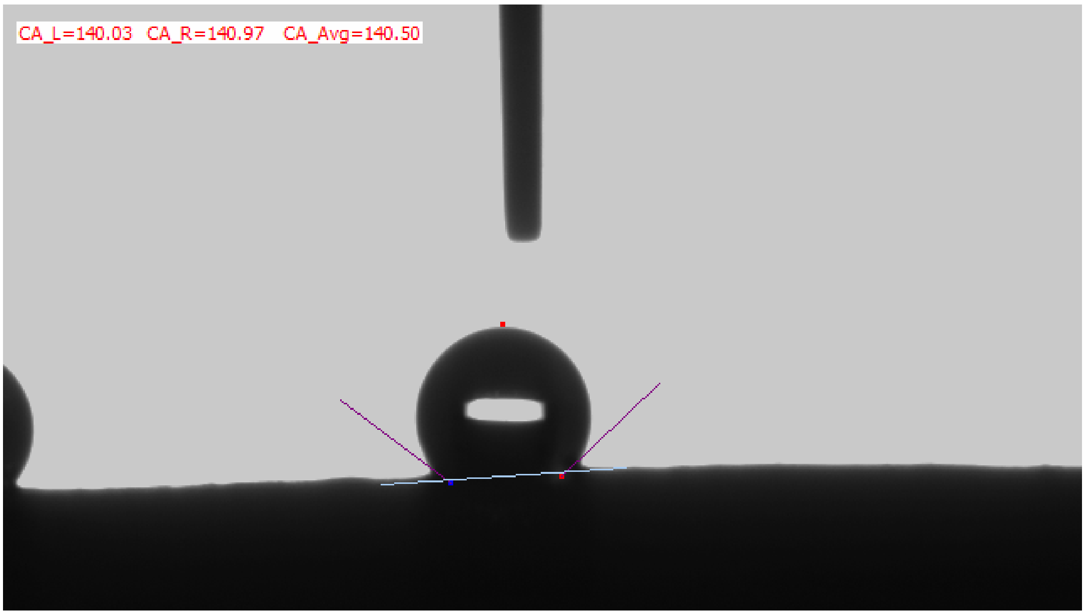
CA_L=140.03 CA_R=140.97 CA_Avg=140.50

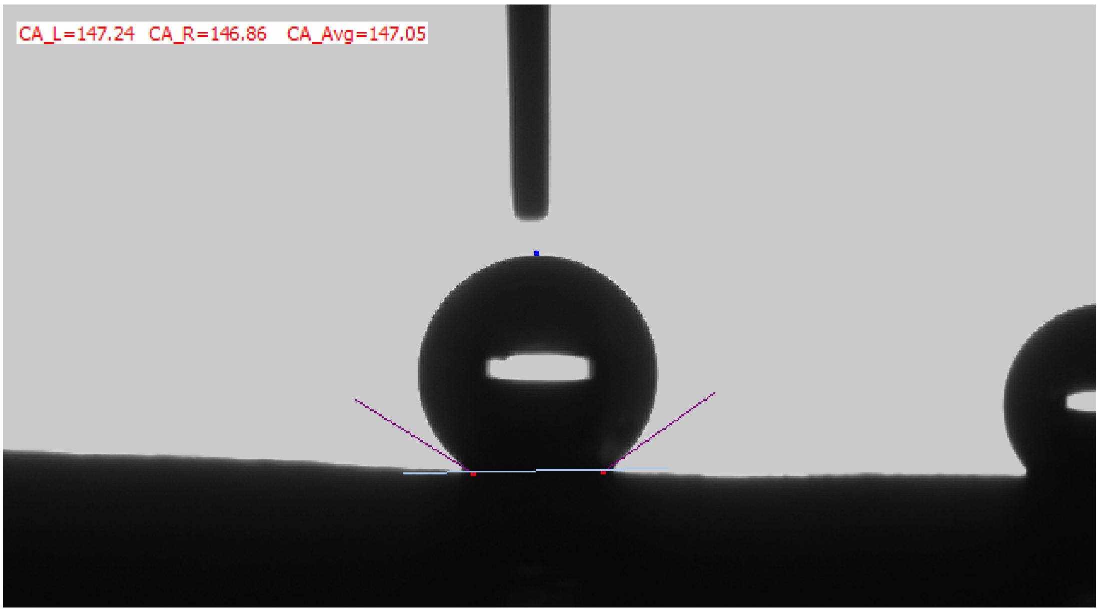
CA_L=147.24 CA_R=146.86 CA_Avg=147.05

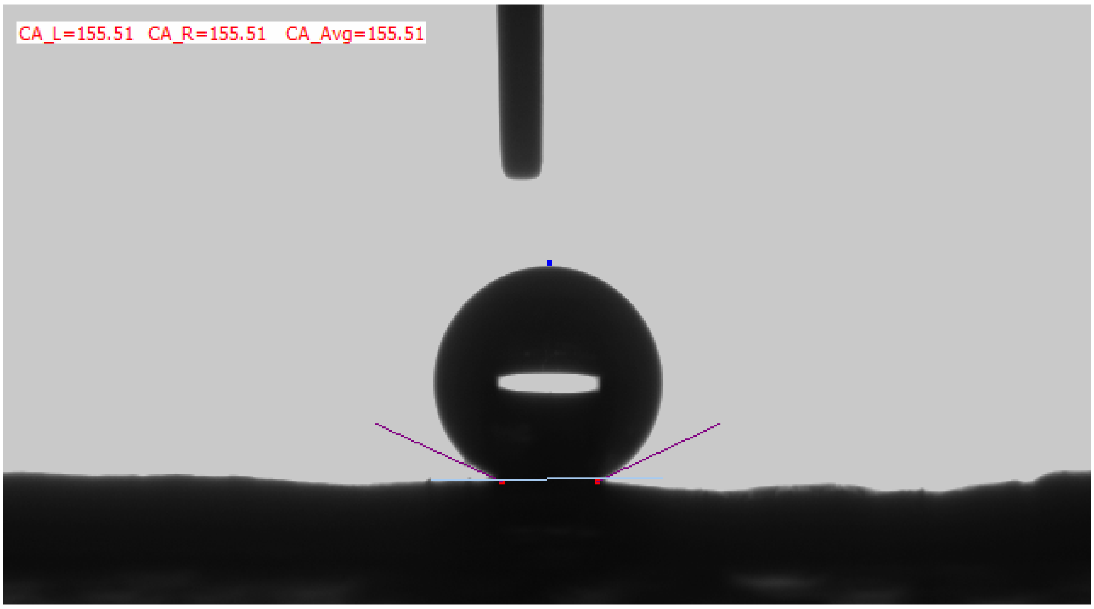
CA_L=155.51 CA_R=155.51 CA_Avg=155.51

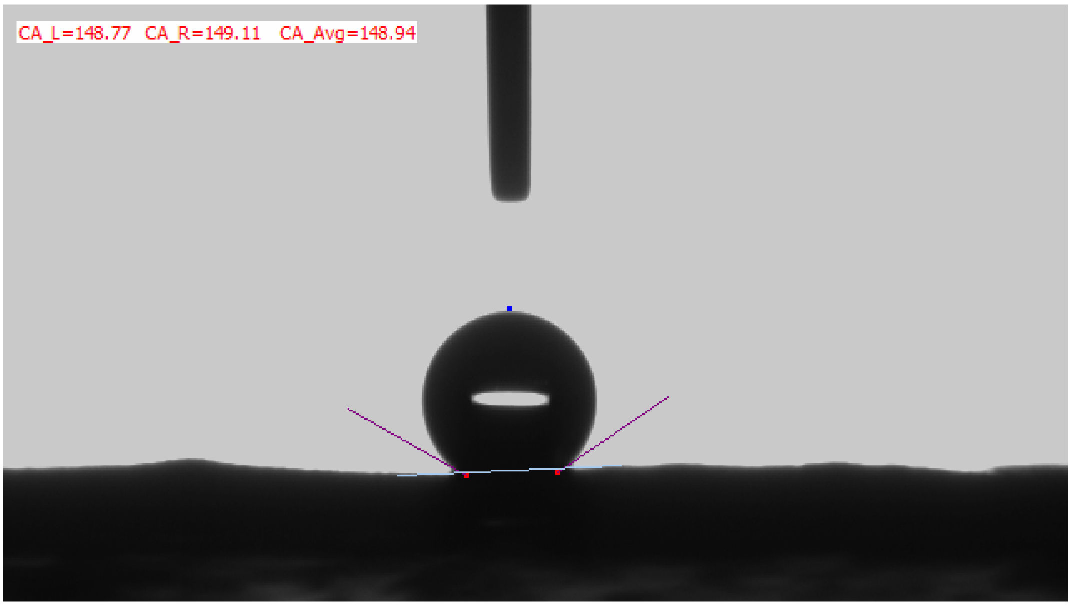
CA_L=148.77 CA_R=149.11 CA_Avg=148.94

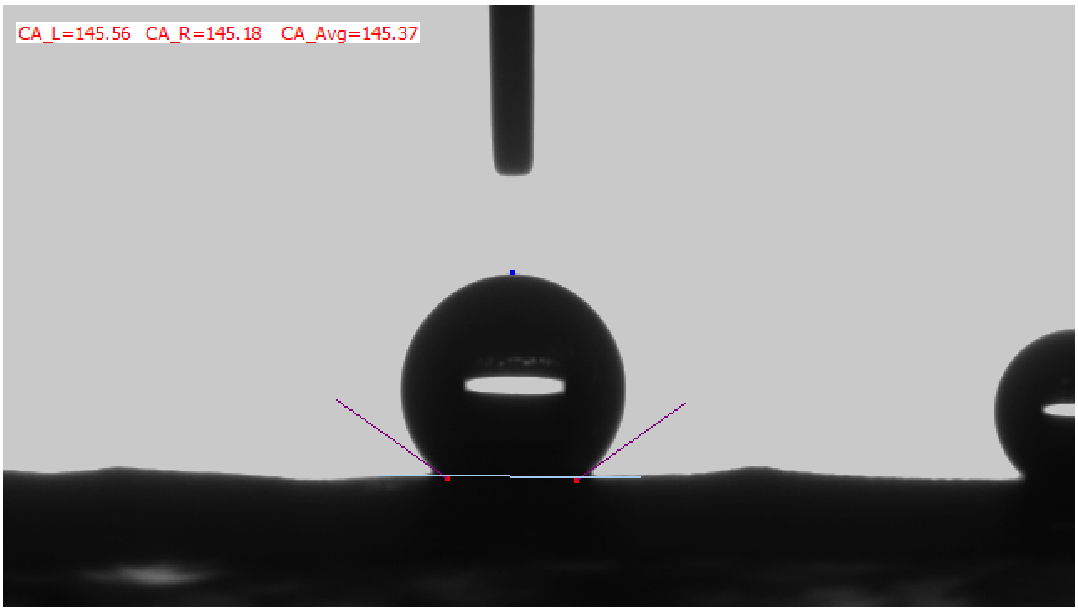
CA_L=145.56 CA_R=145.18 CA_Avg=145.37

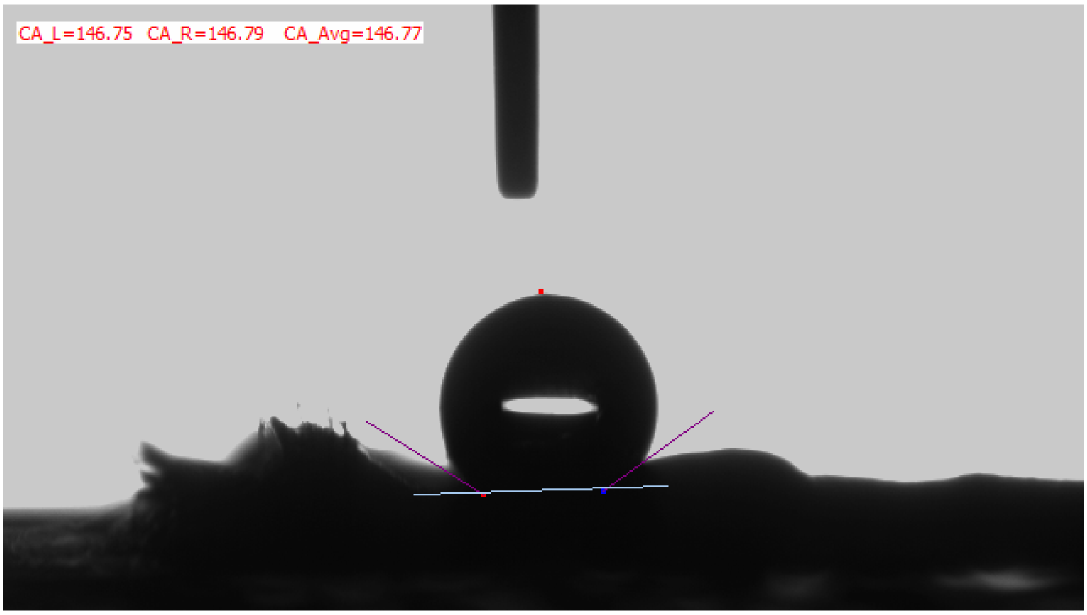
CA_L=146.75 CA_R=146.79 CA_Avg=146.77

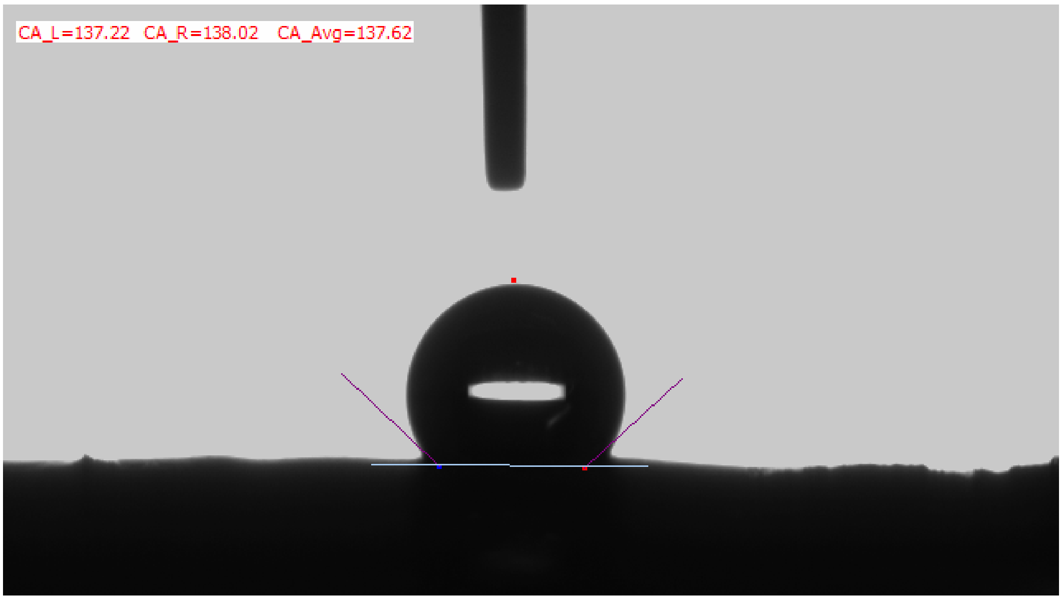
CA_L=137.22 CA_R=138.02   CA_Avg=137.62

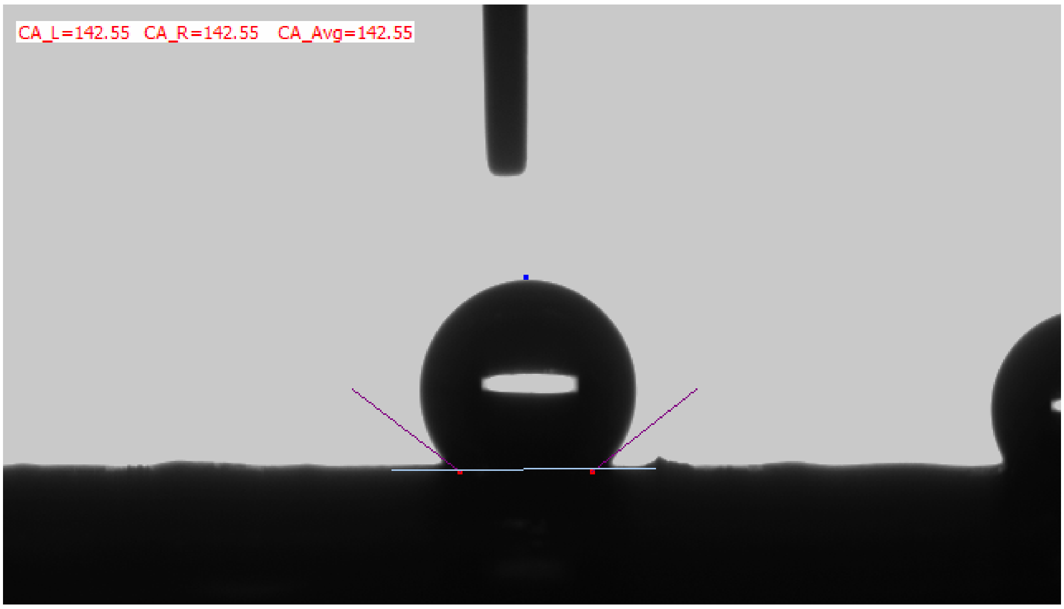
CA_L=142.55 CA_R=142.55   CA_Avg=142.55

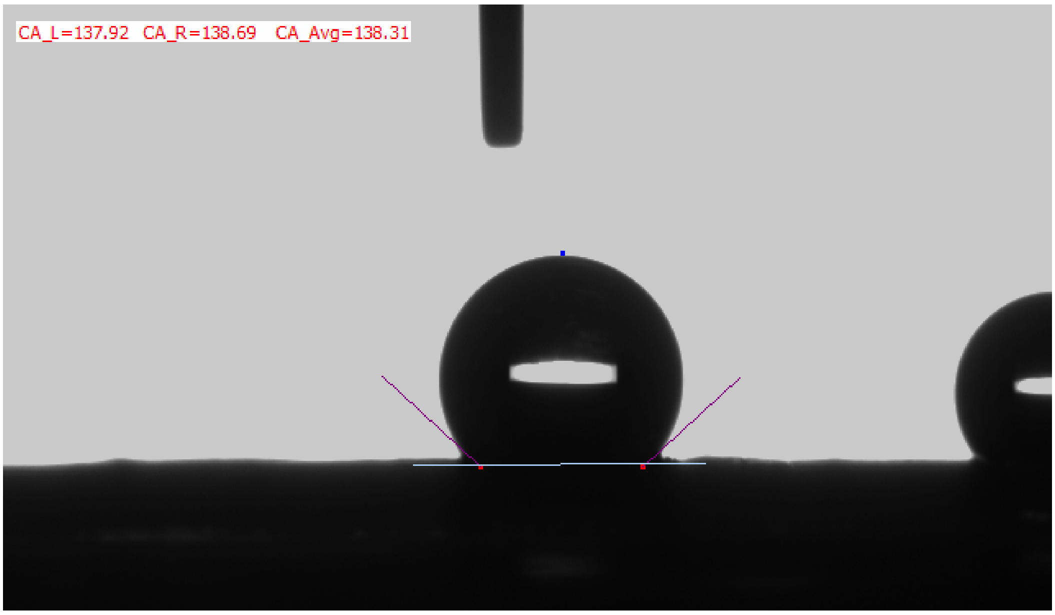
CA_L=137.92 CA_R=138.69   CA_Avg=138.31

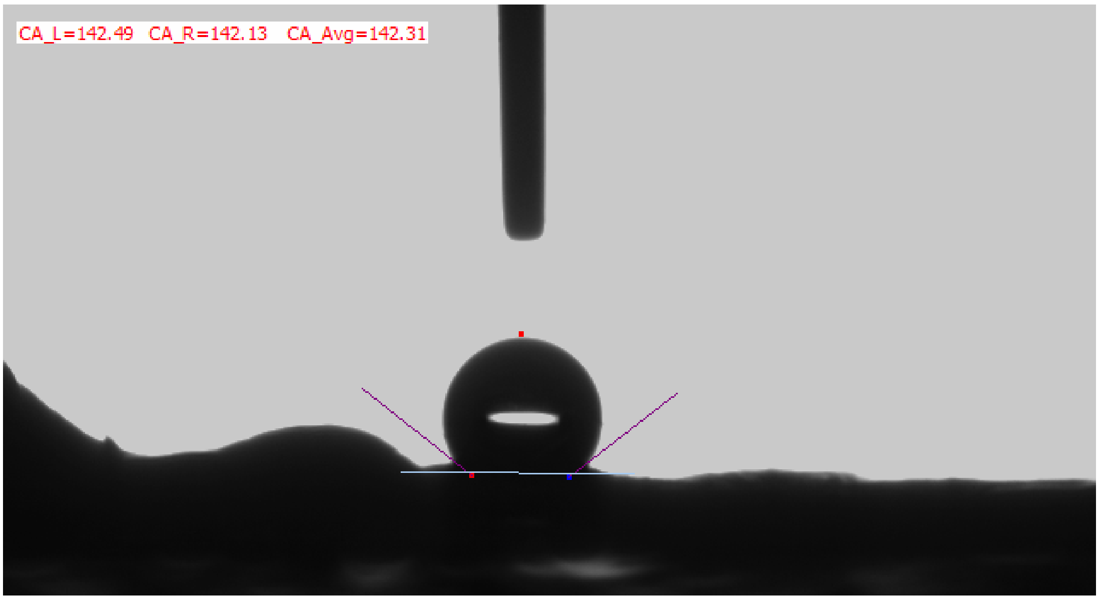
CA_L=142.49 CA_R=142.13 CA_Avg=142.31

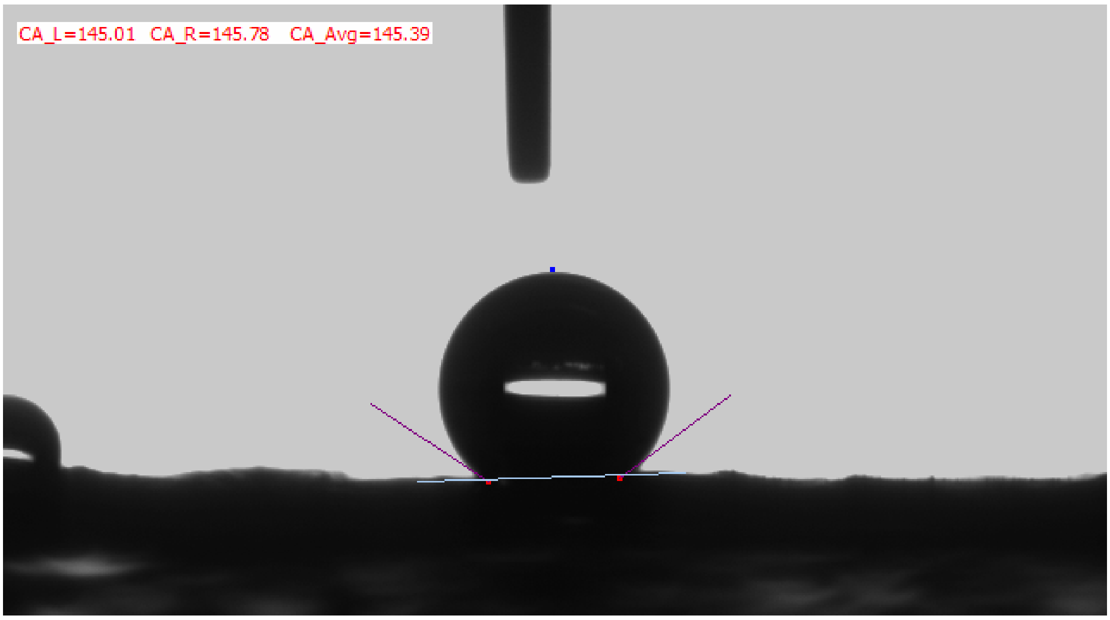
CA_L=145.01 CA_R=145.78 CA_Avg=145.39

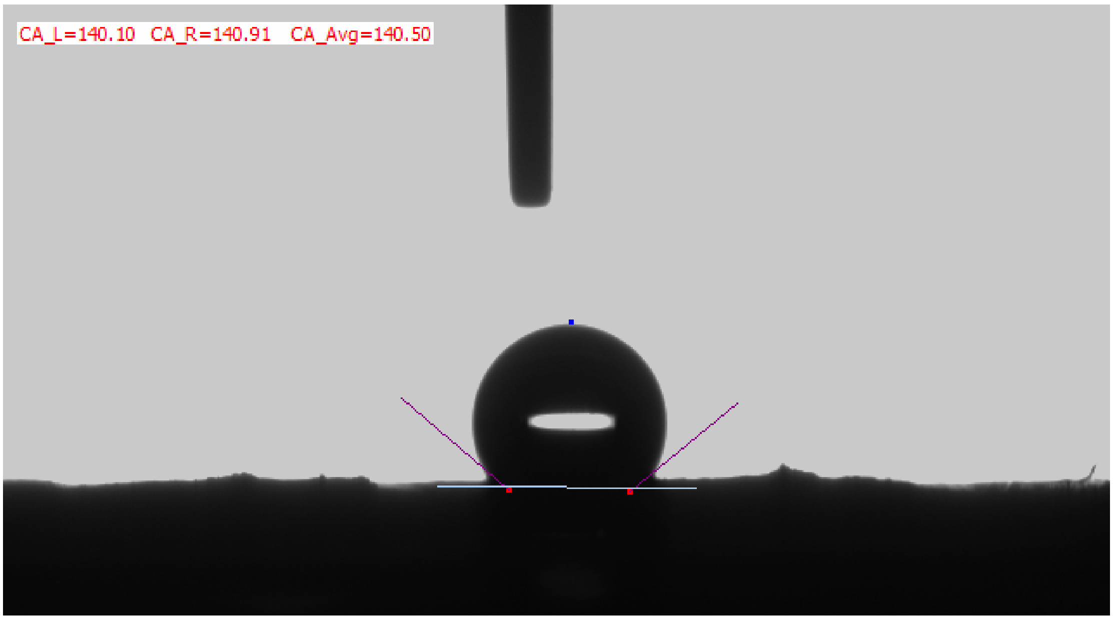
CA_L=140.10 CA_R=140.91 CA_Avg=140.50

S2. Excel data consisting of the static contact angle and surface energy of each sample in studied *Nepenthes* plants.

For this Supplementary Information, please check the datasets-S2.

S3. Excel data involving the statistical information of the *Nepenthes*' static contact angle and surface energy.

For this Supplementary Information, please check the datasets-S3.

S4. Box-plot showing the static contact angle (a) and surface energy (b) of another five *Nepenthes* species.

Note: Horizontal lines represent the median, boxes denote the two inner quartiles, and whiskers represent the maximal and minimal values of the static contact angle and the surface energy.

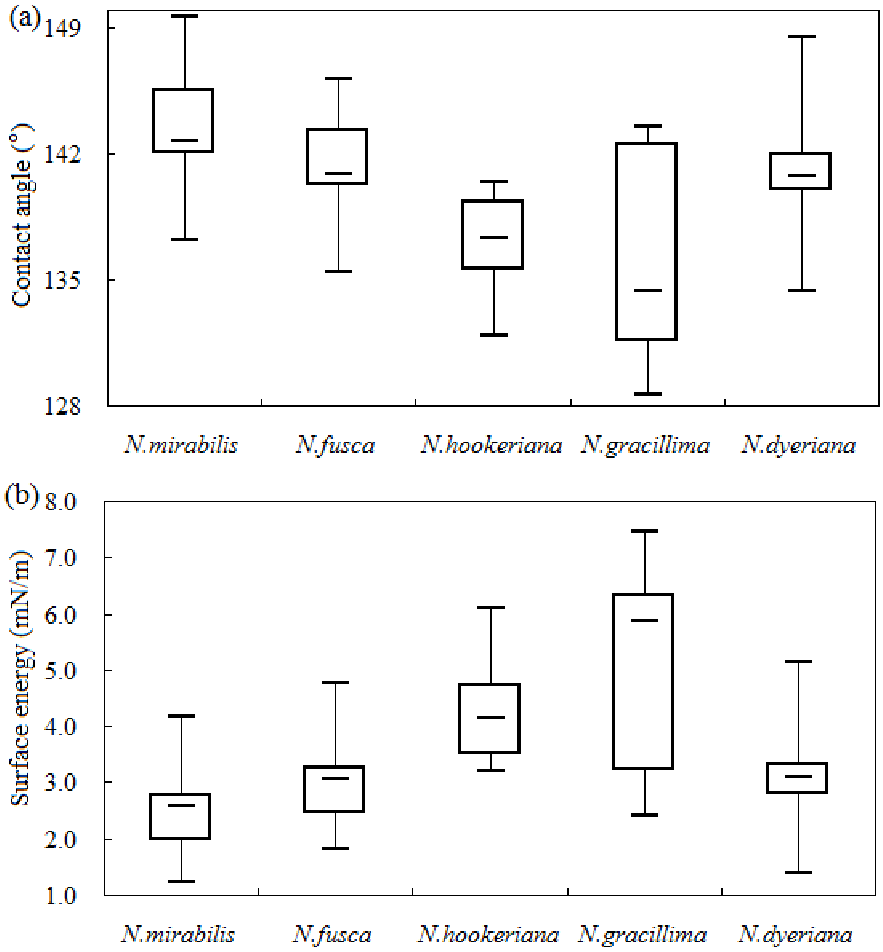

S5. Structural parameters (statistical values) of the slippery surfaces.

For this Supplementary Information, please check the datasets-S5.

S6. Derivation of the Equation 3.

According to the proposed model (Fig. 6a, the slippery surface is simplified as a flat smooth plane with equally distributed triangular prisms of same dimensions), we can obtain the real rough surface area, as follows:

$$A_r = (x+y)l + (a+b)(L-l) + (D-a-b)L$$

$$A_r = (x+y-a-b)l + DL$$

where A_r and L are the real rough surface area and width of the simplified slippery surface, respectively; D is the interval distance of the adjacent triangular prisms (the simplified lunate cells); and l is the length of the triangular prism, $l \le L$.

From the trigonometric function, we obtain the follows:

$$x = \frac{H}{\sin\alpha}, y = \frac{H}{\sin\beta}, a = \frac{H}{\tan\alpha}, b = \frac{H}{\tan\beta}$$

Where α and β are the slope and precipice angles, respectively; H is the height of the triangular prism (the simplified lunate cell).

We also obtain the geometrical surface area (projected area) of the simplified slippery surface, as follows:

$$A_g = DL$$

Based on the above equations, we obtain the roughness factor f_r, as follows:

$$f_r = \frac{A_r}{A_g} = (\frac{1}{\sin\alpha} + \frac{1}{\sin\beta} - \frac{1}{\tan\alpha} - \frac{1}{\tan\beta})\frac{H}{D} \bullet \frac{l}{L} + 1$$

Therefore, following the Wenzel equation (Eq. 1), the Equation 3 can be deduced.

S7. Derivation of the Equation 4.

In the proposed model (Fig. 7a), the slippery surface is simplified as a flat smooth plane with equal-distance distributed triangular prisms and an array of convex cylinders with a particular interval distance. Therefore, the real rough surface area results form two aspects basically: the triangular prisms (lunate cells) and the convex cylinders (platelet-shaped wax crystals).

As shown in S7, the geometrical surface area or the projected area of the simplified slippery surface $A_g = DL$. In this area, the number of convex cylinders can be calculated, as follows:

$$n = DL \bullet R_{wpc} / \pi r_w^2$$

Here, R_{wpc} is the ratio between the area of platelet-shaped wax crystals and the area of the entire slippery surface, r_w represent the radius of the convex cylinder.

For each convex cylinder, the increase of the real rough surface area,

$$A_{cc} = 2\pi r_w h$$

Here, h is the height of the convex cylinder.

The real rough surface area provided by the lunate cells and the wax crystals covered on these lunate cells can be calculated, as follows:

$$A_{r1} = (x+y)l + (x+y)l \bullet \frac{R_{wpc}}{\pi r_w^2} \bullet 2\pi r_w h$$

The real rough surface area provided by the rest part of the simplified slippery surface, can be calculated, as follows:

$$A_{r2} = [DL-(a+b)l] + [DL-(a+b)l] \bullet \frac{R_{wpc}}{\pi r_w^2} \bullet 2\pi r_w h$$

Therefore, we obtain the Equation 4 (roughness factor f_r), as follows:

$$f_r = \frac{A_{r1}+A_{r2}}{A_g} = (1+\frac{2hR_{wpc}}{r_w})[(\frac{1}{\sin\alpha}+\frac{1}{\sin\beta}-\frac{1}{\tan\alpha}-\frac{1}{\tan\beta})\frac{H}{D} \bullet \frac{l}{L}+1]$$

S8. Instructions of the structural parameters (A-wp, R-wpc) obtaining with the software ImageJ.

With the software **ImageJ**, we statistically analyzed and obtained the structural data of wax crystals and Lunate cells, including A_{wp} (Area of single wax platelet in epicuticular wax coverings), R_{wpc} (Ratio between the area of platelet-shaped epicuticular wax coverings and the area of the whole slippery surface), as well as the lunate cell's length, width and distance. Length, width and distance of the lunate cell involve the measurement of line segment, which is very basic, so we here only show the acquisition of A_{wp} and R_{wpc} with the software ImageJ.

Area obtaining of wax platelet with ImageJ

1 Selecting the image obtained from the SEM observation, *Nepenthes alata*,

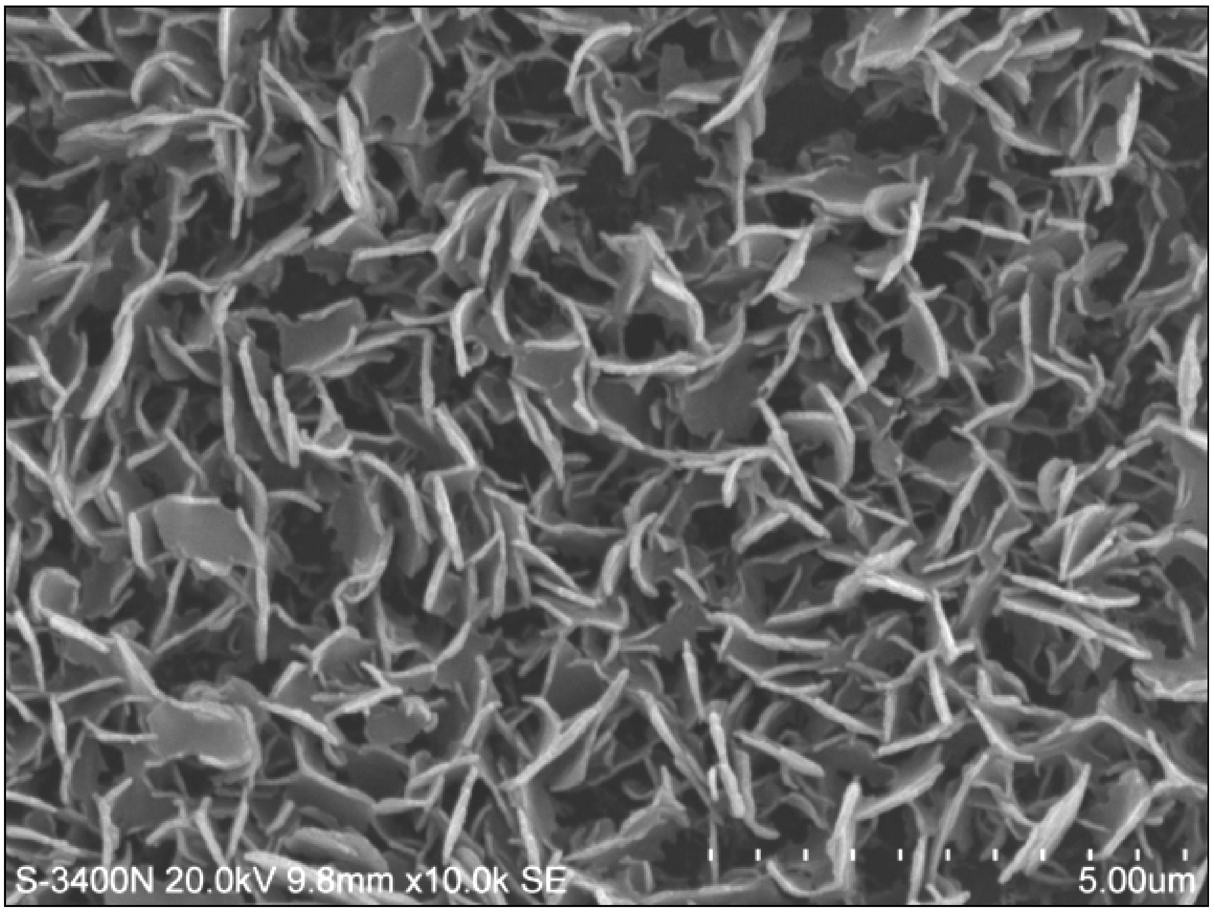

2 Set the scale,

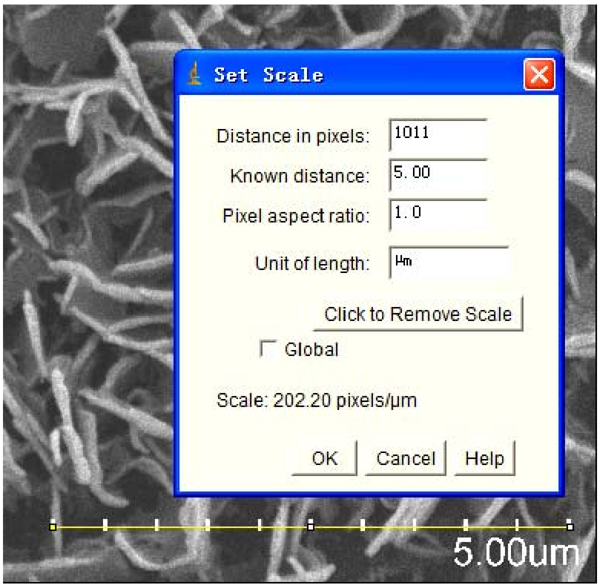

3 Select the “Freehand selections” to draw the profile of the wax platelet,

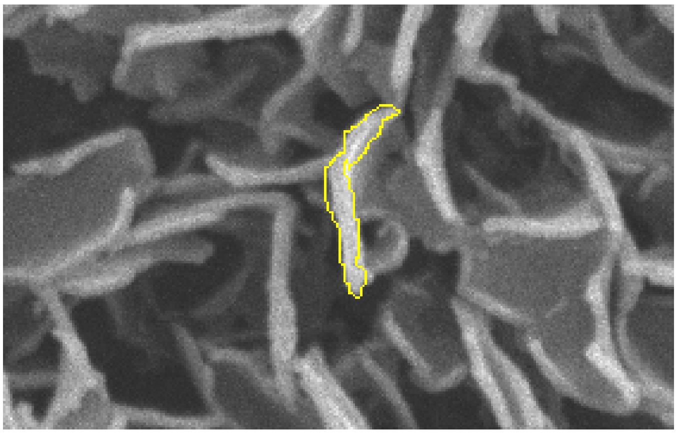

4 Click Analyze-Measure, obtain the area,

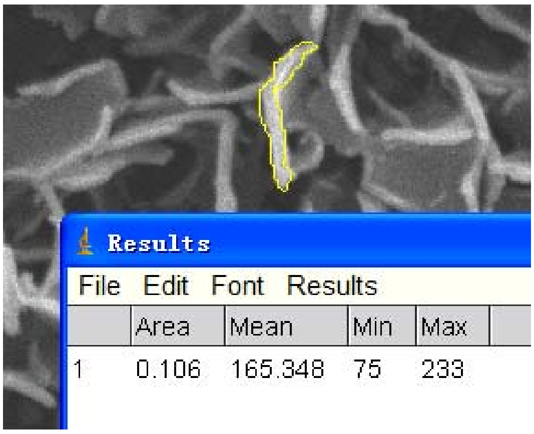

With the same method, we got the area information of the wax platelets in N. alata, N. miranda and *N. khasiana*. For the results, please check the Supplementary Information Datasets-S5.

R_{wpc} **obtaining with ImageJ**

1 Selecting the image obtained from the SEM observation, *Nepenthes alata*,

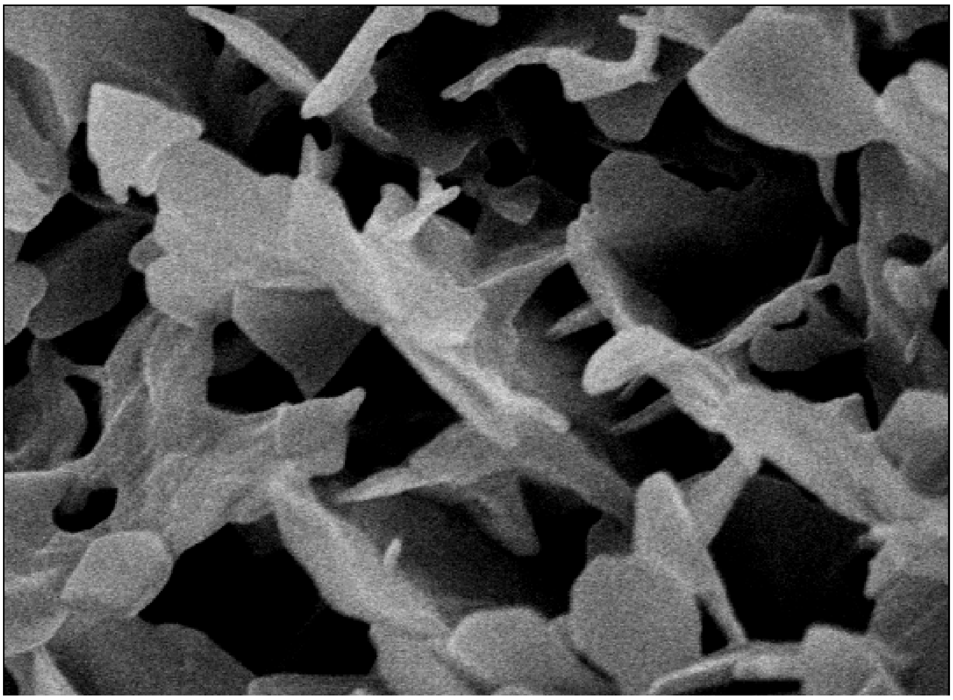

2 Tool bar, click the Image-Adjust-Threshold, the threshold method: Default,

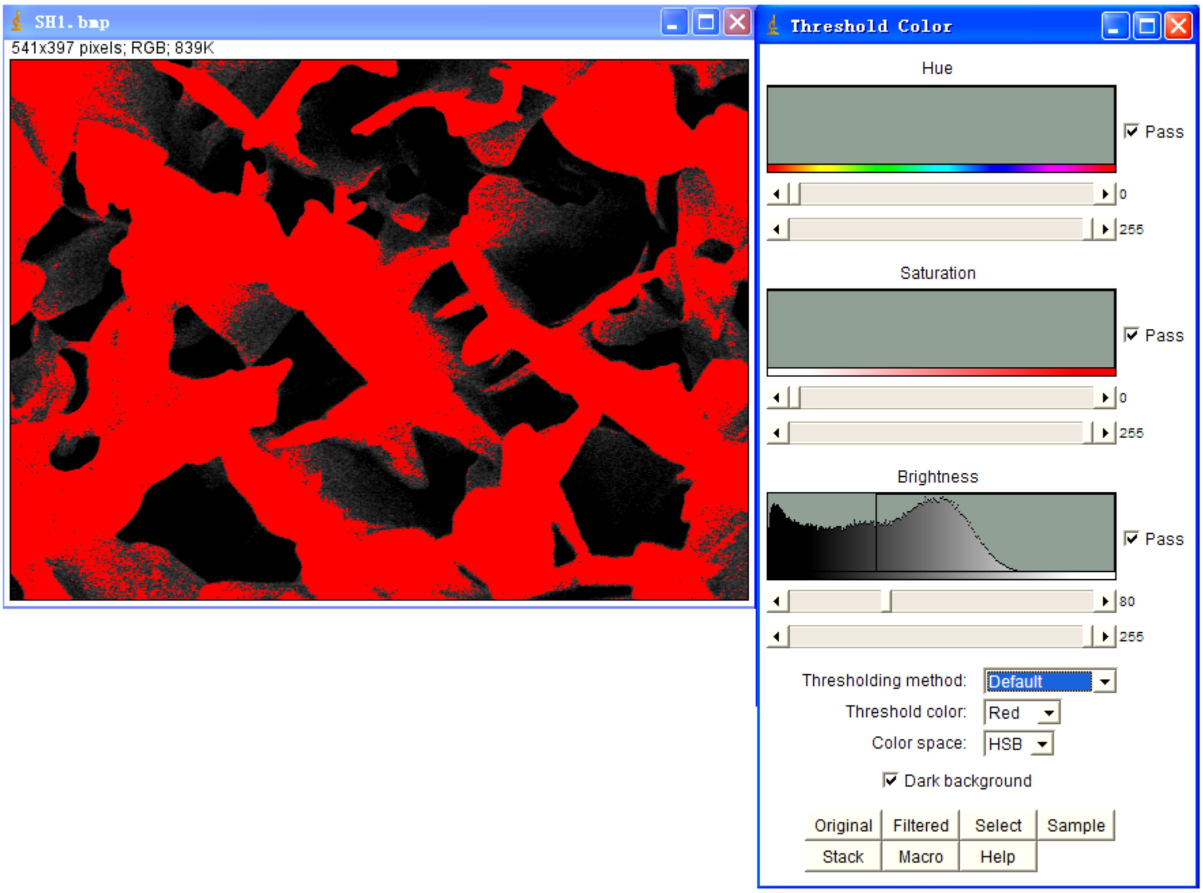

3 Tool bar, click the Analyze-Analyze Particles, select the "Summarize", click OK. We get the result.

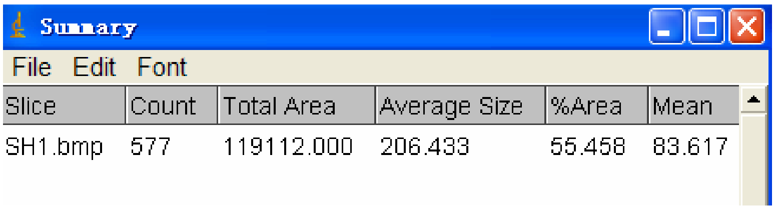

With the same method, we got the R_{wpc} information, for all the results, please check the Supplementary Information Datasets-S5.
